# Megafauna of the UKSRL exploration contract area and eastern Clarion-Clipperton Zone in the Pacific Ocean: Echinodermata

**DOI:** 10.3897/BDJ.5.e11794

**Published:** 2017-05-11

**Authors:** Diva J Amon, Amanda F Ziegler, Antonina Kremenetskaia, Christopher L Mah, Rich Mooi, Tim O'Hara, David L Pawson, Michel Roux, Craig R Smith

**Affiliations:** 1 University of Hawaii at Manoa, Honolulu, United States of America; 2 P.P. Shirshov Institute of Oceanology, Moscow, Russia; 3 Smithsonian Institution National Museum of Natural History, Washington, United States of America; 4 California Academy of Sciences, San Francisco, United States of America; 5 Museum Victoria, Melbourne, Australia; 6 Museum National d’Histoire Naturelle, Paris, France

**Keywords:** deep-sea mining, polymetallic nodule, Clarion-Clipperton Zone, megafauna, echinoderm, atlas

## Abstract

**Background:**

There is growing interest in mining polymetallic nodules from the abyssal Clarion-Clipperton Zone (CCZ) in the tropical Pacific Ocean. Despite being the focus of environmental studies for decades, the benthic megafauna of the CCZ remain poorly known. In order to predict and manage the environmental impacts of mining in the CCZ, baseline knowledge of the megafauna is essential. The ABYSSLINE Project has conducted benthic biological baseline surveys in the UK Seabed Resources Ltd polymetallic-nodule exploration contract area (UK-1). Prior to these research cruises in 2013 and 2015, no biological studies had been done in this area of the eastern CCZ.

**New information:**

Using a Remotely Operated Vehicle and Autonomous Underwater Vehicle, the megafauna within the UKSRL exploration contract area (UK-1) and at a site ~250 km east of the UK-1 area were surveyed, allowing us to make the first estimates of megafaunal morphospecies richness from the imagery collected. Here, we present an atlas of the abyssal echinoderm megafauna observed and collected during the ABYSSLINE cruises to the UK-1 polymetallic-nodule exploration contract area in the CCZ. There appear to be at least 62 distinct morphospecies (13 Asteroidea, 5 Crinoidea, 9 Echinoidea, 29 Holothuroidea and 6 Ophiuroidea) identified mostly by imagery but also using molecular barcoding for a limited number of animals that were collected. This atlas will aid the synthesis of megafaunal presence/absence data collected by contractors, scientists and other stakeholders undertaking work in the CCZ, ultimately helping to decipher the biogeography of the megafauna in this threatened habitat.

## Introduction

**Figure 1.**
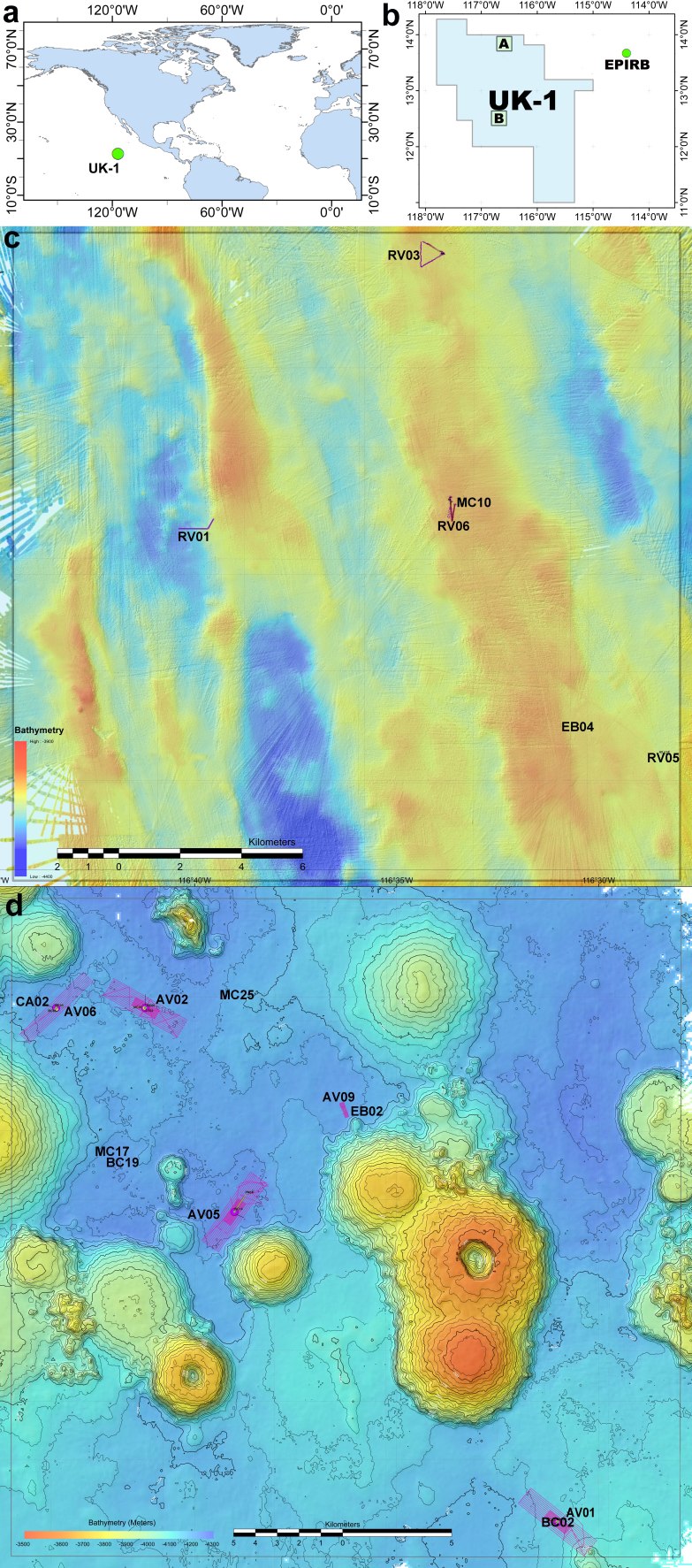
Locations of megafaunal surveys during the ABYSSLINE cruises, AB01 and AB02, in the Clarion-Clipperton Zone. (a) The location of the UKSRL exploration contract area (UK-1) in the eastern Pacific Ocean. (b) The locations of the 30x30-km survey areas, UK-1 Stratum A and UK-1 Stratum B, in relation to the UK-1 exploration contract area and the AB01 ROV dive site, EPIRB, which was approximately 250 km east of the UK-1 contract area. (c) The locations of ROV dives within UK-1 Stratum A, indicated by purple tracklines labelled with the dive number (e.g. RV01). Stations where samples were collected with a Brenke epibenthic sled (EB04) and megacorer (MC10) are also indicated. (d) The locations of AUV dives within UK-1 Stratum B, indicated by purple tracklines labelled with the dive number (e.g. AV01). Stations where samples were collected with a Brenke epibenthic sled (EB02), box corer (BC02, BC19) and megacorer (MC17, MC25) are also indicated, as well as where imagery was collected with a baited camera (CA02). All maps were created by Seafloor Investigations Ltd for the ABYSSLINE Project using ArcGIS software (https://www.arcgis.com/features/).

The Clarion-Clipperton Zone (CCZ) is an abyssal region of the tropical eastern Pacific Ocean where deep-sea mining may take place in the near future (Ramirez-Llodra et al. 2011, Wedding et al. 2013; Fig. 1). High-grade polymetallic nodules, which could provide a commercial source of copper, cobalt, nickel, and manganese (among other metals), are abundant in this six million km^2^ region that lies in Areas Beyond National Jurisdiction (ABNJ), and thus falls under the legal mandate of the International Seabed Authority (ISA) (Wedding et al. 2013). Thus far, there have been 16 exploration leases (each up to 75,000 km^2^ in area) granted by the International Seabed Authority in the CCZ, with those for exploitation expected to soon follow (https://www.isa.org.jm/).

The ABYSSLINE (ABYSSal BaseLINE) Project was designed to undertake benthic biological baseline studies in accordance with ISA environmental guidelines within the UK Seabed Resources Ltd (UKSRL) exploration contract area (UK-1) (Amon et al. 2016b). The UK-1 exploration contract area is one of the easternmost contract areas in the CCZ and encompasses ~58,000 km^2^ of seafloor (Fig. 1). The ABYSSLINE Project was led by scientists from the University of Hawai’i at Mānoa (USA), and included scientists from Hawai’i Pacific University (USA), the Natural History Museum, London (UK), the National Oceanography Centre, Southampton (UK), Senckenberg Gesellschaft für Naturforschung (Germany), Uni Research (Norway), and the International Research Institute of Stavanger (Norway). The ABYSSLINE Project aimed to evaluate baseline conditions of community structure and biodiversity for megafauna, macrofauna, meiofauna and microbes within the UK-1 contract area and across the CCZ (Amon et al. 2016a, Amon et al. 2016b, Dahlgren et al. 2016, Glover et al. 2015, Glover et al. 2016, Shulse et al. 2016). No faunal studies had been undertaken in the UK-1 contract area prior to licensing in 2013.

It is expected that nodule mining will drastically alter this unique deep-sea habitat with recovery expected to be slow (Amon et al. 2016b, Vanreusel et al. 2016, Ramirez-Llodra et al. 2011, Oebius et al. 2001) and yet, despite increases in technology and the number of expeditions to the area, very little is known about the ecology and biogeography of the fauna inhabiting the region (Amon et al. 2016a, Amon et al. 2016b, Dahlgren et al. 2016, Foell and Pawson 1986, Bluhm and Gebruk 1999, Glover et al. 2016, Martinez-Arbizu et al. 2013, Pawson 1983, Pawson and Foell 1986, Roux 2004, Roux and Pawson 1999, Shulse et al. 2016, Tilot 2006, Vanreusel et al. 2016). The megafauna constitute an important component of the biodiversity in the abyssal deep sea and play a significant role in deep-sea ecosystem function (Amon et al. 2016a, Amon et al. 2016b, Smith et al. 2008, Vanreusel et al. 2016). It has also been suggested that echinoderms may act as indicators of physical disturbance of the seabed, such as that caused by deep-sea polymetallic-nodule mining (Bluhm and Gebruk 1999). Glover et al. 2016 reported that a search of OBIS listed 698 echinoderm species recorded at abyssal depths worldwide between 3000m and 6000m, but only 50 species within the CCZ. Amon et al. 2016b was able to confirm that there were no echinoderm or megafauna records for the UK-1 exploration contract area in OBIS. This is likely the result of lack of sampling, taxonomy and/or ensuring data are publicly available, especially as an abundant and diverse echinoderm fauna is already known from the tropical Pacific Ocean from photographic and video survey (Amon et al. 2016a, Amon et al. 2016b, Dahlgren et al. 2016, Foell and Pawson 1986, Glover et al. 2016, Martinez-Arbizu et al. 2013, Pawson 1983, Pawson and Foell 1986, Roux and Pawson 1999, Tilot 2006, Vanreusel et al. 2016). In order to predict and manage the environmental impacts of mining in the CCZ and within the UK-1 exploration contract area, baseline knowledge of the megafauna is essential and allows for a complete taxonomic and biogeographic synthesis of the fauna of the CCZ (Wedding et al. 2015).

Here, we present the first section (Echinodermata) of an image atlas of benthic megafauna that inhabit the UK-1 exploration contract area based on ROV and AUV surveys and samples collected during two cruises of the ABYSSLINE project. This section will be supported by the following sections in the near future: 1) Cnidaria, 2) Porifera, and 3) All Other Phyla (Annelida, Arthropoda, Bryozoa, Chordata, Ctenophora, and Mollusca). This atlas was crucial during the ABYSSLINE quantitative megafaunal analyses (Amon et al. 2016b) and we hope that it will facilitate the standardization of the putative morphospecies and be useful to other scientists and stakeholders undertaking research in the CCZ in the future.

## Materials and methods

The UKSRL exploration contract area (UK-1) is located in the eastern CCZ in the Pacific Ocean (Fig. 1). There have been two ABYSSLINE research cruises to the UK-1 exploration contract area: the AB01 or MV1313 cruise on the R/V *Melville* from 3 to 27 October 2013, and the AB02 or TN319 cruise on the R/V *Thompson* from 12 February to 25 March 2015. The AB01 cruise focused on a 30x30-km stratum (UK-1 Stratum A) centered at 13°49'N, 116°36'W in the northern portion of the UK-1 contract area (Fig. 1). During the AB01 cruise, multibeam bathymetric surveys indicated an abyssal seafloor characterized by ridges and valleys running from NNW to SSE at 3900–4400 m. The commercial Remotely Operated Vehicle (ROV) *Remora III*, operated by Phoenix International Holdings, performed video surveys and sample collections at four randomly-located sites within UK-1 Stratum A in the UK-1 contract area. Additionally, surveys were done ~250 km to the east of the UK-1 contract area, at a site called ‘EPIRB’ centered at 13°40'N, 114°24'W (Fig. 1). Work at the EPIRB site was dictated by an emergency response to an Emergency Position Indicating Radio Beacon (EPIRB) distress signal and, although unplanned, provided a useful broader context for our study. The ROV was equipped with two manipulators, four ROS QLEDIII lights, one 1Cam Alpha Component high-definition downward-looking “science” video camera (1080p video and 24.1 megapixel stills) and one standard-definition forward-looking “pilot” video camera. During surveys, the vehicle had substantial difficulty maintaining constant altitude, direction and velocity over the seabed, thereby limiting the availability of usable imagery and also the collection of specimens.

The AB02 cruise focused on a 30x30-km stratum (UK-1 Stratum B) centered at 12°28'N, 116°36'W in the central portion of the UK-1 exploration contract area (Fig. 1). During the AB02 cruise, multibeam bathymetric surveys indicated an abyssal seafloor dominated by numerous high-relief volcanic seamounts between 3500-4300 m. The Autonomous Underwater Vehicle (AUV) *REMUS 6000*, operated by Woods Hole Oceanographic Institution, performed image surveys at five randomly-located sites within UK-1 Stratum B (Fig. 1). The AUV was equipped with four ROS QLEDIII lights, and one Prosilica GT3400 high-definition downward-looking still camera (9 megapixel stills).

### Sample collection

The ROV was the primary tool used to collect specimens on the AB01 cruise, however due to significant difficulties, a limited number of megafauna was successfully sampled. As a result, megafauna that were collected serendipitously by the box corer, megacorer, and Brenke epibenthic sled were also included in this study (Amon et al. 2016b). As there was no ROV on AB02, we again relied on samples collected by chance with the box corer, megacorer, and Brenke epibenthic sled. Once the respective sampling equipment was on deck, megafauna were quickly transferred to containers of chilled seawater, photographed, and a tissue subsample taken for DNA analyses. DNA samples were preserved in 80% ethanol and the remainder of the animal was preserved in buffered 4% formalin-seawater solution or 95% ethanol, depending on the taxon. On board, all collected specimens were also imaged, with the resulting images included in this manuscript. After the cruise, morphological samples were sent to taxonomic experts for identification and all specimens sequenced for a range of DNA markers at the Natural History Museum, London, with tissue samples subsequently archived and made openly-available for future taxonomic work (Dahlgren et al. 2016, Glover et al. 2015, Glover et al. 2016). All collected specimens were used for taxonomic identifications including ground-truthing identifications based on images.

### Megafaunal image surveys and analyes

All imagery from both “pilot” and “science” cameras on the ROV (covering roughly 8,000 m^2^) collected during AB01 was used during the creation of this atlas (Amon et al. 2016b). All imagery from the AUV (27,178 images covering roughly 500,000 m^2^) collected during AB02 was also used, although the majority of these images (>20,000 images) were at too high an altitude (>6 m) for megafauna to be resolved and identified. All video from both cameras on the ROV, as well as from the AUV, were viewed multiple times and frames archived of each identifiable megafaunal morphotype. The ROV imagery from the AB01 cruise was higher resolution than the imagery collected by the AUV during the AB02 cruise.

The criteria used for selection of megafaunal morphospecies was that individuals were greater than 2 cm in maximum dimension and that there was sufficient detail to identify them to a putative ‘species-level’ morphotype or morphospecies (Amon et al. 2016b). However, this only applied to imagery from AB01 as the AUV imagery collected during AB02 was of poor resolution resulting in only megafauna above 5 cm in the largest dimension being included in this atlas. Morphospecies that could not be identified to species but appeared morphologically distinct were assigned a unique informal species name (e.g. Echinoidea morphospecies 1). These were identified by taxonomic experts or by using the “Atlas of Abyssal Megafauna Morphotypes of the Clarion-Clipperton Fracture Zone” created for the ISA (http://ccfzatlas.com/) (Martinez-Arbizu et al. 2013), as well as Bluhm and Gebruk 1999, Foell and Pawson 1986, Pawson 1983, Pawson and Foell 1986, Roux 2004, Roux and Pawson 1999, Tilot 2006. Morphospecies from this study that matched morphotypes listed in the “Atlas of Abyssal Megafauna Morphotypes of the Clarion-Clipperton Fracture Zone” have had a section titled "Nomenclature" added to their data, in which the identification from the “Atlas of Abyssal Megafauna Morphotypes of the Clarion-Clipperton Fracture Zone” has been included. This is in an effort to provide coherence between these CCZ atlases. For morphospecies that were morphologically similar to a well-defined species name, we use the open nomenclature expression ”cf.", although a precautionary approach was taken. Specimens in this atlas that were collected have undergone (relevant GenBank numbers are included) or are currently undergoing molecular analyses (Glover et al. 2016; Glover et al., unpublished data).

This process provided an estimate of the number of echinoderm morphospecies in the UK-1 contract area and eastern CCZ, and will aid in delimiting species ranges in the CCZ. However, since the majority of the morphospecies were not collected, it is impossible to confirm species identities in most cases or undertake systematic studies on this fauna (Amon et al. 2016b).

## Checklists

### Echinoderms of the UKSRL exploration contract area (UK-1) and the eastern Clarion-Clipperton Zone

#### Echinodermata

#### Asteroidea

de Blainville, 1830

#### Brisingida

Fisher, 1928

#### Freyellidae

Downey, 1986

#### Freyella

Perrier, 1885

#### cf. Freyella morphospecies

##### Materials

- **Type status:** Other material. **Occurrence:** recordedBy: Diva J Amon, Amanda F Ziegler; individualCount: 1; lifeStage: Adult; behavior: Frequently observed on sponge stalks, rocks and seafloor; occurrenceStatus: present; preparations: Imaged only; associatedReferences: 10.1038/srep30492Amon DJ, Ziegler AF, Dahlgren TG, Glover AG, Goineau A, Gooday AJ, Wiklund H, Smith CR. Insights into the abundance and diversity of abyssal megafauna in a polymetallic-nodule region in the eastern Clarion-Clipperton Zone. Scientific Reports. 2016;6. doi: 10.1038/srep30492; **Taxon:** taxonConceptID: *Freyella*cf. *Freyella* morphospecies; scientificName: *FreyellaFreyella* sp.; kingdom: AnimaliaAnimalia; phylum: EchinodermataEchinodermata; class: AsteroideaAsteroidea; order: BrisingidaBrisingida; family: FreyellidaeFreyellidae; genus: FreyellaFreyella; taxonRank: genus; scientificNameAuthorship: Perrier, 1885; **Location:** waterBody: Pacific Ocean; stateProvince: Clarion-Clipperton Zone; locality: UK Seabed Resources Ltd exploration contract area (UK-1); verbatimLocality: UK-1 Stratum A; maximumDepthInMeters: 4020; locationRemarks: RV Melville Cruise MV1313; decimalLatitude: 13.8551; decimalLongitude: -116.5477; geodeticDatum: WGS84; coordinateUncertaintyInMeters: 25; **Identification:** identifiedBy: Christopher Mah, Diva J Amon, Amanda F Ziegler; dateIdentified: 2014; identificationRemarks: Identified only from imagery; identificationQualifier: cf.; **Event:** samplingProtocol: Remotely Operated Vehicle; eventDate: 2013-10-21; eventTime: 3:09; habitat: Abyssal polymetallic-nodule field; fieldNumber: Dive 6 (RV06); **Record Level:** language: en; institutionCode: UHM; datasetName: ABYSSLINE; basisOfRecord: HumanObservation
- **Type status:** Other material. **Occurrence:** recordedBy: Diva J Amon, Amanda F Ziegler; individualCount: 1; lifeStage: Adult; behavior: Frequently observed on sponge stalks, rocks and seafloor; occurrenceStatus: present; preparations: Imaged only; associatedReferences: 10.1038/srep30492Amon DJ, Ziegler AF, Dahlgren TG, Glover AG, Goineau A, Gooday AJ, Wiklund H, Smith CR. Insights into the abundance and diversity of abyssal megafauna in a polymetallic-nodule region in the eastern Clarion-Clipperton Zone. Scientific Reports. 2016;6. doi: 10.1038/srep30492; **Taxon:** taxonConceptID: *Freyella*cf. *Freyella* morphospecies; scientificName: *FreyellaFreyella* sp.; kingdom: AnimaliaAnimalia; phylum: EchinodermataEchinodermata; class: AsteroideaAsteroidea; order: BrisingidaBrisingida; family: FreyellidaeFreyellidae; genus: FreyellaFreyella; taxonRank: genus; scientificNameAuthorship: Perrier, 1885; **Location:** waterBody: Pacific Ocean; stateProvince: Clarion-Clipperton Zone; locality: UK Seabed Resources Ltd exploration contract area (UK-1); verbatimLocality: UK-1 Stratum A; maximumDepthInMeters: 4050; locationRemarks: RV Melville Cruise MV1313; decimalLatitude: 13.9588; decimalLongitude: -116.5605; geodeticDatum: WGS84; coordinateUncertaintyInMeters: 25; **Identification:** identifiedBy: Christopher Mah, Diva J Amon, Amanda F Ziegler; dateIdentified: 2014; identificationRemarks: Identified only from imagery; identificationQualifier: cf.; **Event:** samplingProtocol: Remotely Operated Vehicle; eventDate: 2013-10-16; eventTime: 2:20; habitat: Abyssal polymetallic-nodule field; fieldNumber: Dive 3 (RV03); **Record Level:** language: en; institutionCode: UHM; datasetName: ABYSSLINE; basisOfRecord: HumanObservation
- **Type status:** Other material. **Occurrence:** recordedBy: Diva J Amon, Amanda F Ziegler; individualCount: 1; lifeStage: Adult; behavior: Frequently observed on sponge stalks, rocks and seafloor; occurrenceStatus: present; preparations: Imaged only; associatedReferences: 10.1038/srep30492Amon DJ, Ziegler AF, Dahlgren TG, Glover AG, Goineau A, Gooday AJ, Wiklund H, Smith CR. Insights into the abundance and diversity of abyssal megafauna in a polymetallic-nodule region in the eastern Clarion-Clipperton Zone. Scientific Reports. 2016;6. doi: 10.1038/srep30492; **Taxon:** taxonConceptID: *Freyella*cf. *Freyella* morphospecies; scientificName: *FreyellaFreyella* sp.; kingdom: AnimaliaAnimalia; phylum: EchinodermataEchinodermata; class: AsteroideaAsteroidea; order: BrisingidaBrisingida; family: FreyellidaeFreyellidae; genus: FreyellaFreyella; taxonRank: genus; scientificNameAuthorship: Perrier, 1885; **Location:** waterBody: Pacific Ocean; stateProvince: Clarion-Clipperton Zone; locality: Eastern Clarion-Clipperton Zone; verbatimLocality: Site EPIRB; maximumDepthInMeters: 3943; locationRemarks: RV Melville Cruise MV1313; decimalLatitude: 13.6794; decimalLongitude: -114.4133; geodeticDatum: WGS84; coordinateUncertaintyInMeters: 25; **Identification:** identifiedBy: Christopher Mah, Diva J Amon, Amanda F Ziegler; dateIdentified: 2014; identificationRemarks: Identified only from imagery; identificationQualifier: cf.; **Event:** samplingProtocol: Remotely Operated Vehicle; eventDate: 2013-10-23; eventTime: 10:03; habitat: Abyssal polymetallic-nodule field; fieldNumber: Dive 7 (RV07); **Record Level:** language: en; institutionCode: UHM; datasetName: ABYSSLINE; basisOfRecord: HumanObservation
- **Type status:** Other material. **Occurrence:** recordedBy: Diva J Amon, Amanda F Ziegler; individualCount: 1; lifeStage: Adult; behavior: Frequently observed on sponge stalks, rocks and seafloor; occurrenceStatus: present; preparations: Imaged only; **Taxon:** taxonConceptID: *Freyella*cf. *Freyella* morphospecies; scientificName: *FreyellaFreyella* sp.; kingdom: AnimaliaAnimalia; phylum: EchinodermataEchinodermata; class: AsteroideaAsteroidea; order: BrisingidaBrisingida; family: FreyellidaeFreyellidae; genus: FreyellaFreyella; taxonRank: genus; scientificNameAuthorship: Perrier, 1885; **Location:** waterBody: Pacific Ocean; stateProvince: Clarion-Clipperton Zone; locality: UK Seabed Resources Ltd exploration contract area (UK-1); verbatimLocality: UK-1 Stratum B; maximumDepthInMeters: 4250; locationRemarks: RV Thompson Cruise TN319; decimalLatitude: 12.5011; decimalLongitude: -116.6442; geodeticDatum: WGS84; coordinateUncertaintyInMeters: 25; **Identification:** identifiedBy: Christopher Mah, Diva J Amon, Amanda F Ziegler; dateIdentified: 2015; identificationRemarks: Identified only from imagery; identificationQualifier: cf.; **Event:** samplingProtocol: Autonomous Underwater Vehicle; eventDate: 2015-03-18; eventTime: 11:08; habitat: Abyssal polymetallic-nodule field; fieldNumber: Dive 9 (AV09); **Record Level:** language: en; institutionCode: UHM; datasetName: ABYSSLINE; basisOfRecord: HumanObservation

##### Notes

Fig. 2

#### Freyastera

Sladen, 1889

#### Freyastera cf. benthophila

Sladen, 1889

Freyastera cf. benthophila In the “Atlas of Abyssal Megafauna Morphotypes of the Clarion-Clipperton Fracture Zone” created for the ISA (http://ccfzatlas.com/), this species is listed as "*Freyastera* sp. morphotype".

##### Materials

- **Type status:** Other material. **Occurrence:** recordedBy: Diva J Amon, Amanda F Ziegler; individualCount: 1; lifeStage: Adult; behavior: Frequently observed on sponge stalks, rocks and seafloor; occurrenceStatus: present; preparations: Imaged only; associatedReferences: 10.1038/srep30492Amon DJ, Ziegler AF, Dahlgren TG, Glover AG, Goineau A, Gooday AJ, Wiklund H, Smith CR. Insights into the abundance and diversity of abyssal megafauna in a polymetallic-nodule region in the eastern Clarion-Clipperton Zone. Scientific Reports. 2016;6. doi: 10.1038/srep30492; **Taxon:** taxonConceptID: Freyastera cf. benthophilaFreyastera cf. benthophila; scientificName: Freyastera benthophilaFreyastera benthophila; kingdom: AnimaliaAnimalia; phylum: EchinodermataEchinodermata; class: AsteroideaAsteroidea; order: BrisingidaBrisingida; family: FreyellidaeFreyellidae; genus: FreyasteraFreyastera; taxonRank: species; scientificNameAuthorship: Sladen, 1889; **Location:** waterBody: Pacific Ocean; stateProvince: Clarion-Clipperton Zone; locality: UK Seabed Resources Ltd exploration contract area (UK-1); verbatimLocality: UK-1 Stratum A; maximumDepthInMeters: 4064; locationRemarks: RV Melville Cruise MV1313; decimalLatitude: 13.8635; decimalLongitude: -116.5486; geodeticDatum: WGS84; coordinateUncertaintyInMeters: 25; **Identification:** identifiedBy: Christopher Mah, Diva J Amon, Amanda F Ziegler; dateIdentified: 2014; identificationRemarks: Identified only from imagery; identificationQualifier: cf.; **Event:** samplingProtocol: Remotely Operated Vehicle; eventDate: 2013-10-21; eventTime: 5:07; habitat: Abyssal polymetallic-nodule field; fieldNumber: Dive 6 (RV06); **Record Level:** language: en; institutionCode: UHM; datasetName: ABYSSLINE; basisOfRecord: HumanObservation
- **Type status:** Other material. **Occurrence:** recordedBy: Diva J Amon, Amanda F Ziegler; individualCount: 1; lifeStage: Adult; behavior: Frequently observed on sponge stalks, rocks and seafloor; occurrenceStatus: present; preparations: Imaged only; **Taxon:** taxonConceptID: Freyastera cf. benthophilaFreyastera cf. benthophila; scientificName: Freyastera benthophilaFreyastera benthophila; kingdom: AnimaliaAnimalia; phylum: EchinodermataEchinodermata; class: AsteroideaAsteroidea; order: BrisingidaBrisingida; family: FreyellidaeFreyellidae; genus: FreyasteraFreyastera; taxonRank: species; scientificNameAuthorship: Sladen, 1889; **Location:** waterBody: Pacific Ocean; stateProvince: Clarion-Clipperton Zone; locality: UK Seabed Resources Ltd exploration contract area (UK-1); verbatimLocality: UK-1 Stratum B; maximumDepthInMeters: 4251; locationRemarks: RV Thompson Cruise TN319; decimalLatitude: 12.4979; decimalLongitude: -116.6464; geodeticDatum: WGS84; coordinateUncertaintyInMeters: 25; **Identification:** identifiedBy: Christopher Mah, Diva J Amon, Amanda F Ziegler; dateIdentified: 2015; identificationRemarks: Identified only from imagery; identificationQualifier: cf.; **Event:** samplingProtocol: Autonomous Underwater Vehicle; eventDate: 2015-03-03; eventTime: 22:38; habitat: Abyssal polymetallic-nodule field; fieldNumber: Dive 5 (AV05); **Record Level:** language: en; institutionCode: UHM; datasetName: ABYSSLINE; basisOfRecord: HumanObservation
- **Type status:** Other material. **Occurrence:** recordedBy: Diva J Amon, Amanda F Ziegler; individualCount: 1; lifeStage: Adult; behavior: Frequently observed on sponge stalks, rocks and seafloor; occurrenceStatus: present; preparations: Imaged only; associatedReferences: 10.1038/srep30492Amon DJ, Ziegler AF, Dahlgren TG, Glover AG, Goineau A, Gooday AJ, Wiklund H, Smith CR. Insights into the abundance and diversity of abyssal megafauna in a polymetallic-nodule region in the eastern Clarion-Clipperton Zone. Scientific Reports. 2016;6. doi: 10.1038/srep30492; **Taxon:** taxonConceptID: Freyastera cf. benthophilaFreyastera cf. benthophila; scientificName: Freyastera benthophilaFreyastera benthophila; kingdom: AnimaliaAnimalia; phylum: EchinodermataEchinodermata; class: AsteroideaAsteroidea; order: BrisingidaBrisingida; family: FreyellidaeFreyellidae; genus: FreyasteraFreyastera; taxonRank: species; scientificNameAuthorship: Sladen, 1889; **Location:** waterBody: Pacific Ocean; stateProvince: Clarion-Clipperton Zone; locality: UK Seabed Resources Ltd exploration contract area (UK-1); verbatimLocality: UK-1 Stratum A; maximumDepthInMeters: 4027; locationRemarks: RV Melville Cruise MV1313; decimalLatitude: 13.8609; decimalLongitude: -116.5468; geodeticDatum: WGS84; coordinateUncertaintyInMeters: 25; **Identification:** identifiedBy: Christopher Mah, Diva J Amon, Amanda F Ziegler; dateIdentified: 2014; identificationRemarks: Identified only from imagery; identificationQualifier: cf.; **Event:** samplingProtocol: Remotely Operated Vehicle; eventDate: 2013-10-21; eventTime: 1:55; habitat: Abyssal polymetallic-nodule field; fieldNumber: Dive 6 (RV06); **Record Level:** language: en; institutionCode: UHM; datasetName: ABYSSLINE; basisOfRecord: HumanObservation
- **Type status:** Other material. **Occurrence:** catalogNumber: AB01-RV06-CS-10; recordNumber: AB01-RV06-CS-10; NHM413; recordedBy: Diva J Amon, Amanda F Ziegler; individualCount: 1; lifeStage: Adult; behavior: Frequently observed on sponge stalks, rocks and seafloor; occurrenceStatus: present; preparations: tissue and DNA voucher stored in 80% non-denatured ethanol aqueous solution and remainder of animal preserved in 4% formaldehyde; otherCatalogNumbers: b7ffe7a2-7be1-4d4fb784-7aaecf0ee743; 5023520; associatedReferences: 10.1038/srep30492Echinodermata10.3897/BDJ.4.e7251Amon DJ, Ziegler AF, Dahlgren TG, Glover AG, Goineau A, Gooday AJ, Wiklund H, Smith CR. Insights into the abundance and diversity of abyssal megafauna in a polymetallic-nodule region in the eastern Clarion-Clipperton Zone. Scientific Reports. 2016;6. doi: 10.1038/srep30492 | Glover AG, Wiklund H, Rabone M, Amon DJ, Smith CR, O'Hara T, Mah CL, Dahlgren TG. Abyssal fauna of the UK-1 polymetallic nodule exploration claim, Clarion-Clipperton Zone, central Pacific Ocean: Echinodermata. Biodiversity data journal. 2016(4). doi: 10.3897/BDJ.4.e7251; associatedSequences: http://www.ncbi.nlm.nih.gov/nuccore/KU519550KU519518KU519535http://www.ncbi.nlm.nih.gov/nuccore/KU519550 | KU519518 | KU519535; **Taxon:** taxonConceptID: Freyastera cf. benthophilaFreyastera cf. benthophila; scientificName: Freyastera benthophilaFreyastera benthophila; kingdom: AnimaliaAnimalia; phylum: EchinodermataEchinodermata; class: AsteroideaAsteroidea; order: BrisingidaBrisingida; family: FreyellidaeFreyellidae; genus: FreyasteraFreyastera; taxonRank: species; scientificNameAuthorship: Sladen, 1889; **Location:** waterBody: Pacific Ocean; stateProvince: Clarion-Clipperton Zone; locality: UK Seabed Resources Ltd exploration contract area (UK-1); verbatimLocality: UK-1 Stratum A; maximumDepthInMeters: 4011; locationRemarks: RV Melville Cruise MV1313; decimalLatitude: 13.8622; decimalLongitude: -116.5462; geodeticDatum: WGS84; coordinateUncertaintyInMeters: 25; **Identification:** identifiedBy: Christopher Mah, Diva J Amon, Amanda F Ziegler, Adrian Glover, Helena Wiklund, Thomas Dahlgren; dateIdentified: 2014; identificationRemarks: Identified by morphology and DNA of collected specimen; identificationQualifier: cf.; **Event:** samplingProtocol: Remotely Operated Vehicle; eventDate: 2013-10-21; eventTime: 0:39; habitat: Abyssal polymetallic-nodule field; fieldNumber: Dive 6 (RV06); **Record Level:** language: en; institutionCode: UHM; datasetName: ABYSSLINE; basisOfRecord: PreservedSpecimen

##### Notes

Fig. 3

#### Paxillosida

Perrier, 1884

#### cf. Paxillosida morphospecies 1

##### Materials

- **Type status:** Other material. **Occurrence:** recordedBy: Diva J Amon, Amanda F Ziegler; individualCount: 1; lifeStage: Adult; behavior: On seafloor; occurrenceStatus: present; preparations: Imaged only; associatedReferences: 10.1038/srep30492Amon DJ, Ziegler AF, Dahlgren TG, Glover AG, Goineau A, Gooday AJ, Wiklund H, Smith CR. Insights into the abundance and diversity of abyssal megafauna in a polymetallic-nodule region in the eastern Clarion-Clipperton Zone. Scientific Reports. 2016;6. doi: 10.1038/srep30492; **Taxon:** taxonConceptID: Paxillosidacf. Paxillosida morphospecies 1; scientificName: PaxillosidaPaxillosida sp.; kingdom: AnimaliaAnimalia; phylum: EchinodermataEchinodermata; class: AsteroideaAsteroidea; order: PaxillosidaPaxillosida; taxonRank: order; scientificNameAuthorship: Perrier, 1884; **Location:** waterBody: Pacific Ocean; stateProvince: Clarion-Clipperton Zone; locality: UK Seabed Resources Ltd exploration contract area (UK-1); verbatimLocality: UK-1 Stratum A; maximumDepthInMeters: 4022; locationRemarks: RV Melville Cruise MV1313; decimalLatitude: 13.8593; decimalLongitude: -116.5482; geodeticDatum: WGS84; coordinateUncertaintyInMeters: 25; **Identification:** identifiedBy: Christopher Mah, Diva J Amon, Amanda F Ziegler; dateIdentified: 2014; identificationRemarks: Identified only from imagery; identificationQualifier: cf.; **Event:** samplingProtocol: Remotely Operated Vehicle; eventDate: 2013-10-21; eventTime: 4:12; habitat: Abyssal polymetallic-nodule field; fieldNumber: Dive 6 (RV06); **Record Level:** language: en; institutionCode: UHM; datasetName: ABYSSLINE; basisOfRecord: HumanObservation

##### Notes

Fig. 4

#### cf. Paxillosida morphospecies 2

##### Materials

- **Type status:** Other material. **Occurrence:** recordedBy: Diva J Amon, Amanda F Ziegler; individualCount: 1; lifeStage: Adult; behavior: On seafloor; occurrenceStatus: present; preparations: Imaged only; **Taxon:** taxonConceptID: Paxillosidacf. Paxillosida morphospecies 2; scientificName: PaxillosidaPaxillosida sp.; kingdom: AnimaliaAnimalia; phylum: EchinodermataEchinodermata; class: AsteroideaAsteroidea; order: PaxillosidaPaxillosida; taxonRank: order; scientificNameAuthorship: Perrier, 1884; **Location:** waterBody: Pacific Ocean; stateProvince: Clarion-Clipperton Zone; locality: UK Seabed Resources Ltd exploration contract area (UK-1); verbatimLocality: UK-1 Stratum B; maximumDepthInMeters: 4144; locationRemarks: RV Thompson Cruise TN319; decimalLatitude: 12.3606; decimalLongitude: -116.5133; geodeticDatum: WGS84; coordinateUncertaintyInMeters: 25; **Identification:** identifiedBy: Christopher Mah, Diva J Amon, Amanda F Ziegler; dateIdentified: 2015; identificationRemarks: Identified only from imagery; identificationQualifier: cf.; **Event:** samplingProtocol: Autonomous Underwater Vehicle; eventDate: 2015-02-18; eventTime: 20:09; habitat: Abyssal polymetallic-nodule field; fieldNumber: Dive 1 (AV01); **Record Level:** language: en; institutionCode: UHM; datasetName: ABYSSLINE; basisOfRecord: HumanObservation

##### Notes

Fig. 5

#### cf. Paxillosida morphospecies 3

cf. Paxillosida morphospecies 3 In the “Atlas of Abyssal Megafauna Morphotypes of the Clarion-Clipperton Fracture Zone” created for the ISA (http://ccfzatlas.com/), this morphospecies is listed as "Astropectinidae*Dytaster* morphotype".

##### Materials

- **Type status:** Other material. **Occurrence:** recordedBy: Diva J Amon, Amanda F Ziegler; individualCount: 1; lifeStage: Adult; behavior: On seafloor; occurrenceStatus: present; preparations: Imaged only; **Taxon:** taxonConceptID: Paxillosidacf. Paxillosida morphospecies 3; scientificName: PaxillosidaPaxillosida sp.; kingdom: AnimaliaAnimalia; phylum: EchinodermataEchinodermata; class: AsteroideaAsteroidea; order: PaxillosidaPaxillosida; taxonRank: order; scientificNameAuthorship: Perrier, 1884; **Location:** waterBody: Pacific Ocean; stateProvince: Clarion-Clipperton Zone; locality: UK Seabed Resources Ltd exploration contract area (UK-1); verbatimLocality: UK-1 Stratum B; maximumDepthInMeters: 4159; locationRemarks: RV Thompson Cruise TN319; decimalLatitude: 12.3655; decimalLongitude: -116.5184; geodeticDatum: WGS84; coordinateUncertaintyInMeters: 25; **Identification:** identifiedBy: Christopher Mah, Diva J Amon, Amanda F Ziegler; dateIdentified: 2015; identificationRemarks: Identified only from imagery; identificationQualifier: cf.; **Event:** samplingProtocol: Autonomous Underwater Vehicle; eventDate: 2015-02-18; eventTime: 20:56; habitat: Abyssal polymetallic-nodule field; fieldNumber: Dive 1 (AV01); **Record Level:** language: en; institutionCode: UHM; datasetName: ABYSSLINE; basisOfRecord: HumanObservation

##### Notes

Fig. 6

#### Porcellanasteridae

Sladen, 1883

#### cf. Porcellanasteridae morphospecies

##### Materials

- **Type status:** Other material. **Occurrence:** recordedBy: Diva J Amon, Amanda F Ziegler; individualCount: 1; lifeStage: Adult; behavior: On seafloor; occurrenceStatus: present; preparations: Imaged only; associatedReferences: 10.1038/srep30492Amon DJ, Ziegler AF, Dahlgren TG, Glover AG, Goineau A, Gooday AJ, Wiklund H, Smith CR. Insights into the abundance and diversity of abyssal megafauna in a polymetallic-nodule region in the eastern Clarion-Clipperton Zone. Scientific Reports. 2016;6. doi: 10.1038/srep30492; **Taxon:** taxonConceptID: Porcellanasteridaecf. Porcellanasteridae morphospecies; scientificName: PorcellanasteridaePorcellanasteridae sp.; kingdom: AnimaliaAnimalia; phylum: EchinodermataEchinodermata; class: AsteroideaAsteroidea; order: PaxillosidaPaxillosida; family: PorcellanasteridaePorcellanasteridae; taxonRank: family; scientificNameAuthorship: Sladen, 1883; **Location:** waterBody: Pacific Ocean; stateProvince: Clarion-Clipperton Zone; locality: UK Seabed Resources Ltd exploration contract area (UK-1); verbatimLocality: UK-1 Stratum A; maximumDepthInMeters: 4029; locationRemarks: RV Melville Cruise MV1313; decimalLatitude: 13.8632; decimalLongitude: -116.5464; geodeticDatum: WGS84; coordinateUncertaintyInMeters: 25; **Identification:** identifiedBy: Christopher Mah, Diva J Amon, Amanda F Ziegler; dateIdentified: 2014; identificationRemarks: Identified only from imagery; identificationQualifier: cf.; **Event:** samplingProtocol: Remotely Operated Vehicle; eventDate: 2013-10-21; eventTime: 9:11; habitat: Abyssal polymetallic-nodule field; fieldNumber: Dive 6 (RV06); **Record Level:** language: en; institutionCode: UHM; datasetName: ABYSSLINE; basisOfRecord: HumanObservation
- **Type status:** Other material. **Occurrence:** recordedBy: Diva J Amon, Amanda F Ziegler; individualCount: 1; lifeStage: Adult; behavior: On seafloor; occurrenceStatus: present; preparations: Imaged only; associatedReferences: 10.1038/srep30492Amon DJ, Ziegler AF, Dahlgren TG, Glover AG, Goineau A, Gooday AJ, Wiklund H, Smith CR. Insights into the abundance and diversity of abyssal megafauna in a polymetallic-nodule region in the eastern Clarion-Clipperton Zone. Scientific Reports. 2016;6. doi: 10.1038/srep30492; **Taxon:** taxonConceptID: Porcellanasteridaecf. Porcellanasteridae morphospecies; scientificName: PorcellanasteridaePorcellanasteridae sp.; kingdom: AnimaliaAnimalia; phylum: EchinodermataEchinodermata; class: AsteroideaAsteroidea; order: PaxillosidaPaxillosida; family: PorcellanasteridaePorcellanasteridae; taxonRank: family; scientificNameAuthorship: Sladen, 1883; **Location:** waterBody: Pacific Ocean; stateProvince: Clarion-Clipperton Zone; locality: Eastern Clarion-Clipperton Zone; verbatimLocality: Site EPIRB; maximumDepthInMeters: 3950; locationRemarks: RV Melville Cruise MV1313; decimalLatitude: 13.6798; decimalLongitude: -114.4144; geodeticDatum: WGS84; coordinateUncertaintyInMeters: 25; **Identification:** identifiedBy: Christopher Mah, Diva J Amon, Amanda F Ziegler; dateIdentified: 2014; identificationRemarks: Identified only from imagery; identificationQualifier: cf.; **Event:** samplingProtocol: Remotely Operated Vehicle; eventDate: 2013-10-23; eventTime: 13:27; habitat: Abyssal polymetallic-nodule field; fieldNumber: Dive 7 (RV07); **Record Level:** language: en; institutionCode: UHM; datasetName: ABYSSLINE; basisOfRecord: HumanObservation

##### Notes

Fig. 7

#### Porcellanaster

Wyville Thomson, 1877

#### cf. Porcellanaster morphospecies

##### Materials

- **Type status:** Other material. **Occurrence:** recordedBy: Diva J Amon, Amanda F Ziegler; individualCount: 1; lifeStage: Adult; behavior: On seafloor; occurrenceStatus: present; preparations: Imaged only; associatedReferences: 10.1038/srep30492Amon DJ, Ziegler AF, Dahlgren TG, Glover AG, Goineau A, Gooday AJ, Wiklund H, Smith CR. Insights into the abundance and diversity of abyssal megafauna in a polymetallic-nodule region in the eastern Clarion-Clipperton Zone. Scientific Reports. 2016;6. doi: 10.1038/srep30492; **Taxon:** taxonConceptID: *Porcellanaster*cf. *Porcellanaster* morphospecies; scientificName: *PorcellanasterPorcellanaster* sp.; kingdom: AnimaliaAnimalia; phylum: EchinodermataEchinodermata; class: AsteroideaAsteroidea; order: PaxillosidaPaxillosida; family: PorcellanasteridaePorcellanasteridae; genus: PorcellanasterPorcellanaster; taxonRank: genus; scientificNameAuthorship: Wyville Thomson, 1877; **Location:** waterBody: Pacific Ocean; stateProvince: Clarion-Clipperton Zone; locality: UK Seabed Resources Ltd exploration contract area (UK-1); verbatimLocality: UK-1 Stratum A; maximumDepthInMeters: 4110; locationRemarks: RV Melville Cruise MV1313; decimalLatitude: 13.8501; decimalLongitude: -116.6456; geodeticDatum: WGS84; coordinateUncertaintyInMeters: 25; **Identification:** identifiedBy: Christopher Mah, Diva J Amon, Amanda F Ziegler; dateIdentified: 2014; identificationRemarks: Identified only from imagery; identificationQualifier: cf.; **Event:** samplingProtocol: Remotely Operated Vehicle; eventDate: 2013-10-10; eventTime: 16:24; habitat: Abyssal polymetallic-nodule field; fieldNumber: Dive 1 (RV01); **Record Level:** language: en; institutionCode: UHM; datasetName: ABYSSLINE; basisOfRecord: HumanObservation
- **Type status:** Other material. **Occurrence:** recordedBy: Diva J Amon, Amanda F Ziegler; individualCount: 1; lifeStage: Adult; behavior: On seafloor; occurrenceStatus: present; preparations: Imaged only; associatedReferences: 10.1038/srep30492Amon DJ, Ziegler AF, Dahlgren TG, Glover AG, Goineau A, Gooday AJ, Wiklund H, Smith CR. Insights into the abundance and diversity of abyssal megafauna in a polymetallic-nodule region in the eastern Clarion-Clipperton Zone. Scientific Reports. 2016;6. doi: 10.1038/srep30492; **Taxon:** taxonConceptID: *Porcellanaster*cf. *Porcellanaster* morphospecies; scientificName: *PorcellanasterPorcellanaster* sp.; kingdom: AnimaliaAnimalia; phylum: EchinodermataEchinodermata; class: AsteroideaAsteroidea; order: PaxillosidaPaxillosida; family: PorcellanasteridaePorcellanasteridae; genus: PorcellanasterPorcellanaster; taxonRank: genus; scientificNameAuthorship: Wyville Thomson, 1877; **Location:** waterBody: Pacific Ocean; stateProvince: Clarion-Clipperton Zone; locality: Eastern Clarion-Clipperton Zone; verbatimLocality: Site EPIRB; maximumDepthInMeters: 3947; locationRemarks: RV Melville Cruise MV1313; decimalLatitude: 13.6794; decimalLongitude: -114.4137; geodeticDatum: WGS84; coordinateUncertaintyInMeters: 25; **Identification:** identifiedBy: Christopher Mah, Diva J Amon, Amanda F Ziegler; dateIdentified: 2014; identificationRemarks: Identified only from imagery; identificationQualifier: cf.; **Event:** samplingProtocol: Remotely Operated Vehicle; eventDate: 2013-10-23; eventTime: 9:58; habitat: Abyssal polymetallic-nodule field; fieldNumber: Dive 7 (RV07); **Record Level:** language: en; institutionCode: UHM; datasetName: ABYSSLINE; basisOfRecord: HumanObservation

##### Notes

Fig. 8

#### Porcellanaster ceruleus

Wyville Thomson, 1877

##### Materials

- **Type status:** Other material. **Occurrence:** catalogNumber: AB01-EB04-NHM253; recordNumber: AB01-EB04-NHM253; recordedBy: Diva J Amon, Amanda F Ziegler; individualCount: 2; lifeStage: Adult; behavior: On seafloor; occurrenceStatus: present; preparations: tissue and DNA voucher stored in 80% non-denatured ethanol aqueous solution and remainder of animal preserved in 4% formaldehyde; otherCatalogNumbers: 95d0bd7f-0df9-47e4-8003-cd12007d54b4; associatedReferences: 10.1038/srep30492Echinodermata10.3897/BDJ.4.e7251Amon DJ, Ziegler AF, Dahlgren TG, Glover AG, Goineau A, Gooday AJ, Wiklund H, Smith CR. Insights into the abundance and diversity of abyssal megafauna in a polymetallic-nodule region in the eastern Clarion-Clipperton Zone. Scientific Reports. 2016;6. doi: 10.1038/srep30492 | Glover AG, Wiklund H, Rabone M, Amon DJ, Smith CR, O'Hara T, Mah CL, Dahlgren TG. Abyssal fauna of the UK-1 polymetallic nodule exploration claim, Clarion-Clipperton Zone, central Pacific Ocean: Echinodermata. Biodiversity data journal. 2016(4). doi: 10.3897/BDJ.4.e7251; associatedSequences: http://www.ncbi.nlm.nih.gov/nuccore/KU519570KU519525KU519542http://www.ncbi.nlm.nih.gov/nuccore/KU519570 | KU519525 | KU519542; **Taxon:** taxonConceptID: Porcellanaster ceruleusPorcellanaster ceruleus; scientificName: Porcellanaster ceruleusPorcellanaster ceruleus; kingdom: AnimaliaAnimalia; phylum: EchinodermataEchinodermata; class: AsteroideaAsteroidea; order: PaxillosidaPaxillosida; family: PorcellanasteridaePorcellanasteridae; genus: PorcellanasterPorcellanaster; taxonRank: species; scientificNameAuthorship: Wyville Thomson, 1877; **Location:** waterBody: Pacific Ocean; stateProvince: Clarion-Clipperton Zone; locality: UK Seabed Resources Ltd exploration contract area (UK-1); verbatimLocality: UK-1 Stratum A; maximumDepthInMeters: 4076; locationRemarks: RV Melville Cruise MV1313; decimalLatitude: 13.7558; decimalLongitude: -116.4867; geodeticDatum: WGS84; coordinateUncertaintyInMeters: 500; **Identification:** identifiedBy: Christopher Mah, Diva J Amon, Amanda F Ziegler, Adrian Glover, Helena Wiklund, Thomas Dahlgren; dateIdentified: 2014; identificationRemarks: Identified by morphology and DNA of collected specimen; identificationQualifier: cf.; **Event:** samplingProtocol: Brenke Epibenthic Sled; eventDate: 2013-10-17; eventTime: 1:50; habitat: Abyssal polymetallic-nodule field; fieldNumber: Brenke Epibenthic Sled (EB04); **Record Level:** language: en; institutionCode: NHMUK; collectionCode: ZOO; datasetName: ABYSSLINE; basisOfRecord: PreservedSpecimen

##### Notes

Fig. 9

#### Styracaster

Sladen, 1883

#### Styracaster paucispinus

Ludwig, 1907

##### Materials

- **Type status:** Other material. **Occurrence:** catalogNumber: AB02-MC17-CS-31; recordNumber: AB02-MC17-CS-31; NHM1608; recordedBy: Diva J Amon, Amanda F Ziegler; individualCount: 1; lifeStage: Adult; behavior: On seafloor; occurrenceStatus: present; preparations: tissue and DNA voucher stored in 80% non-denatured ethanol aqueous solution and remainder of animal preserved in 4% formaldehyde; associatedReferences: 10.1038/srep30492Echinodermata10.3897/BDJ.4.e7251Amon DJ, Ziegler AF, Dahlgren TG, Glover AG, Goineau A, Gooday AJ, Wiklund H, Smith CR. Insights into the abundance and diversity of abyssal megafauna in a polymetallic-nodule region in the eastern Clarion-Clipperton Zone. Scientific Reports. 2016;6. doi: 10.1038/srep30492 | Glover AG, Wiklund H, Rabone M, Amon DJ, Smith CR, O'Hara T, Mah CL, Dahlgren TG. Abyssal fauna of the UK-1 polymetallic nodule exploration claim, Clarion-Clipperton Zone, central Pacific Ocean: Echinodermata. Biodiversity data journal. 2016(4). doi: 10.3897/BDJ.4.e7251; **Taxon:** taxonConceptID: Syracaster paucispinusSyracaster paucispinus; scientificName: Syracaster paucispinusSyracaster paucispinus; kingdom: AnimaliaAnimalia; phylum: EchinodermataEchinodermata; class: AsteroideaAsteroidea; order: PaxillosidaPaxillosida; family: PorcellanasteridaePorcellanasteridae; genus: StyracasterStyracaster; taxonRank: species; scientificNameAuthorship: Ludwig, 1907; **Location:** waterBody: Pacific Ocean; stateProvince: Clarion-Clipperton Zone; locality: UK Seabed Resources Ltd exploration contract area (UK-1); verbatimLocality: UK-1 Stratum B; maximumDepthInMeters: 4237; locationRemarks: RV Thompson Cruise TN319; decimalLatitude: 12.5212; decimalLongitude: -116.698; geodeticDatum: WGS84; coordinateUncertaintyInMeters: 50; **Identification:** identifiedBy: Christopher Mah, Diva J Amon, Amanda F Ziegler, Adrian Glover, Helena Wiklund, Thomas Dahlgren; dateIdentified: 2015; identificationRemarks: Identified by morphology and DNA of collected specimen; identificationQualifier: cf.; **Event:** samplingProtocol: Megacorer; eventDate: 2015-03-09; eventTime: 10:56; habitat: Abyssal polymetallic-nodule field; fieldNumber: Megacorer 17 (MC17); **Record Level:** language: en; institutionCode: UHM; datasetName: ABYSSLINE; basisOfRecord: PreservedSpecimen

##### Notes

Fig. 10

#### Velatida

Perrier, 1884

#### Pterasteridae

Perrier, 1875

#### cf. Pterasteridae morphospecies 1

cf. Pterasteridae morphospecies 1 In the “Atlas of Abyssal Megafauna Morphotypes of the Clarion-Clipperton Fracture Zone” created for the ISA (http://ccfzatlas.com/), this morphospecies is listed as "*Pteraster* morphotype".

##### Materials

- **Type status:** Other material. **Occurrence:** recordedBy: Diva J Amon, Amanda F Ziegler; individualCount: 1; lifeStage: Adult; behavior: On seafloor; occurrenceStatus: present; preparations: Imaged only; associatedReferences: 10.1038/srep30492Amon DJ, Ziegler AF, Dahlgren TG, Glover AG, Goineau A, Gooday AJ, Wiklund H, Smith CR. Insights into the abundance and diversity of abyssal megafauna in a polymetallic-nodule region in the eastern Clarion-Clipperton Zone. Scientific Reports. 2016;6. doi: 10.1038/srep30492; **Taxon:** taxonConceptID: Pterasteridaecf. Pterasteridae morphospecies 1; scientificName: PterasteridaePterasteridae sp.; kingdom: AnimaliaAnimalia; phylum: EchinodermataEchinodermata; class: AsteroideaAsteroidea; order: VelatidaVelatida; family: PterasteridaePterasteridae; taxonRank: family; scientificNameAuthorship: Perrier, 1875; **Location:** waterBody: Pacific Ocean; stateProvince: Clarion-Clipperton Zone; locality: UK Seabed Resources Ltd exploration contract area (UK-1); verbatimLocality: UK-1 Stratum A; maximumDepthInMeters: 4059; locationRemarks: RV Melville Cruise MV1313; decimalLatitude: 13.9669; decimalLongitude: -116.55796; geodeticDatum: WGS84; coordinateUncertaintyInMeters: 25; **Identification:** identifiedBy: Christopher Mah, Diva J Amon, Amanda F Ziegler; dateIdentified: 2014; identificationRemarks: Identified only from imagery; identificationQualifier: cf.; **Event:** samplingProtocol: Remotely Operated Vehicle; eventDate: 2013-10-16; eventTime: 0:06; habitat: Abyssal polymetallic-nodule field; fieldNumber: Dive 3 (RV03); **Record Level:** language: en; institutionCode: UHM; datasetName: ABYSSLINE; basisOfRecord: HumanObservation
- **Type status:** Other material. **Occurrence:** recordedBy: Diva J Amon, Amanda F Ziegler; individualCount: 1; lifeStage: Adult; behavior: On seafloor; occurrenceStatus: present; preparations: Imaged only; associatedReferences: 10.1038/srep30492Amon DJ, Ziegler AF, Dahlgren TG, Glover AG, Goineau A, Gooday AJ, Wiklund H, Smith CR. Insights into the abundance and diversity of abyssal megafauna in a polymetallic-nodule region in the eastern Clarion-Clipperton Zone. Scientific Reports. 2016;6. doi: 10.1038/srep30492; **Taxon:** taxonConceptID: Pterasteridaecf. Pterasteridae morphospecies 1; scientificName: PterasteridaePterasteridae sp.; kingdom: AnimaliaAnimalia; phylum: EchinodermataEchinodermata; class: AsteroideaAsteroidea; order: VelatidaVelatida; family: PterasteridaePterasteridae; taxonRank: family; scientificNameAuthorship: Perrier, 1875; **Location:** waterBody: Pacific Ocean; stateProvince: Clarion-Clipperton Zone; locality: UK Seabed Resources Ltd exploration contract area (UK-1); verbatimLocality: UK-1 Stratum B; maximumDepthInMeters: 4226; locationRemarks: RV Thompson Cruise TN319; decimalLatitude: 12.5783; decimalLongitude: -116.6872; geodeticDatum: WGS84; coordinateUncertaintyInMeters: 25; **Identification:** identifiedBy: Christopher Mah, Diva J Amon, Amanda F Ziegler; dateIdentified: 2015; identificationRemarks: Identified only from imagery; identificationQualifier: cf.; **Event:** samplingProtocol: Autonomous Underwater Vehicle; eventDate: 2015-03-09; eventTime: 3:12; habitat: Abyssal polymetallic-nodule field; fieldNumber: Dive 6 (AV06); **Record Level:** language: en; institutionCode: UHM; datasetName: ABYSSLINE; basisOfRecord: HumanObservation

##### Notes

Fig. 11

#### cf. Pterasteridae morphospecies 2

##### Materials

- **Type status:** Other material. **Occurrence:** recordedBy: Diva J Amon, Amanda F Ziegler; individualCount: 1; lifeStage: Adult; behavior: On seafloor; occurrenceStatus: present; preparations: Imaged only; associatedReferences: 10.1038/srep30492Amon DJ, Ziegler AF, Dahlgren TG, Glover AG, Goineau A, Gooday AJ, Wiklund H, Smith CR. Insights into the abundance and diversity of abyssal megafauna in a polymetallic-nodule region in the eastern Clarion-Clipperton Zone. Scientific Reports. 2016;6. doi: 10.1038/srep30492; **Taxon:** taxonConceptID: Pterasteridaecf. Pterasteridae morphospecies 2; scientificName: PterasteridaePterasteridae sp.; kingdom: AnimaliaAnimalia; phylum: EchinodermataEchinodermata; class: AsteroideaAsteroidea; order: VelatidaVelatida; family: PterasteridaePterasteridae; taxonRank: family; scientificNameAuthorship: Perrier, 1875; **Location:** waterBody: Pacific Ocean; stateProvince: Clarion-Clipperton Zone; locality: UK Seabed Resources Ltd exploration contract area (UK-1); verbatimLocality: UK-1 Stratum A; maximumDepthInMeters: 4107; locationRemarks: RV Melville Cruise MV1313; decimalLatitude: 13.85096; decimalLongitude: -116.6453; geodeticDatum: WGS84; coordinateUncertaintyInMeters: 25; **Identification:** identifiedBy: Christopher Mah, Diva J Amon, Amanda F Ziegler; dateIdentified: 2014; identificationRemarks: Identified only from imagery; identificationQualifier: cf.; **Event:** samplingProtocol: Remotely Operated Vehicle; eventDate: 2013-10-10; eventTime: 16:48; habitat: Abyssal polymetallic-nodule field; fieldNumber: Dive 1 (RV01); **Record Level:** language: en; institutionCode: UHM; datasetName: ABYSSLINE; basisOfRecord: HumanObservation

##### Notes

Fig. 12

#### Hymenaster

Thomson, 1873

#### cf. Hymenaster morphospecies 1

##### Materials

- **Type status:** Other material. **Occurrence:** recordedBy: Diva J Amon, Amanda F Ziegler; individualCount: 1; lifeStage: Adult; behavior: On seafloor; occurrenceStatus: present; preparations: Imaged only; associatedReferences: 10.1038/srep30492Amon DJ, Ziegler AF, Dahlgren TG, Glover AG, Goineau A, Gooday AJ, Wiklund H, Smith CR. Insights into the abundance and diversity of abyssal megafauna in a polymetallic-nodule region in the eastern Clarion-Clipperton Zone. Scientific Reports. 2016;6. doi: 10.1038/srep30492; **Taxon:** taxonConceptID: *Hymenaster*cf. *Hymenaster* morphospecies 1; scientificName: *HymenasterHymenaster* sp.; kingdom: AnimaliaAnimalia; phylum: EchinodermataEchinodermata; class: AsteroideaAsteroidea; order: VelatidaVelatida; family: PterasteridaePterasteridae; genus: HymenasterHymenaster; taxonRank: genus; scientificNameAuthorship: Thomson, 1873; **Location:** waterBody: Pacific Ocean; stateProvince: Clarion-Clipperton Zone; locality: UK Seabed Resources Ltd exploration contract area (UK-1); verbatimLocality: UK-1 Stratum A; maximumDepthInMeters: 4027; locationRemarks: RV Melville Cruise MV1313; decimalLatitude: 13.86097; decimalLongitude: -116.5468; geodeticDatum: WGS84; coordinateUncertaintyInMeters: 25; **Identification:** identifiedBy: Christopher Mah, Diva J Amon, Amanda F Ziegler; dateIdentified: 2014; identificationRemarks: Identified only from imagery; identificationQualifier: cf.; **Event:** samplingProtocol: Remotely Operated Vehicle; eventDate: 2013-10-21; eventTime: 1:54; habitat: Abyssal polymetallic-nodule field; fieldNumber: Dive 6 (RV06); **Record Level:** language: en; institutionCode: UHM; datasetName: ABYSSLINE; basisOfRecord: HumanObservation

##### Notes

Fig. 13

#### cf. Hymenaster morphospecies 2

##### Materials

- **Type status:** Other material. **Occurrence:** recordedBy: Diva J Amon, Amanda F Ziegler; individualCount: 1; lifeStage: Adult; behavior: On seafloor; occurrenceStatus: present; preparations: Imaged only; associatedReferences: 10.1038/srep30492Amon DJ, Ziegler AF, Dahlgren TG, Glover AG, Goineau A, Gooday AJ, Wiklund H, Smith CR. Insights into the abundance and diversity of abyssal megafauna in a polymetallic-nodule region in the eastern Clarion-Clipperton Zone. Scientific Reports. 2016;6. doi: 10.1038/srep30492; **Taxon:** taxonConceptID: *Hymenaster*cf. *Hymenaster* morphospecies 2; scientificName: *HymenasterHymenaster* sp.; kingdom: AnimaliaAnimalia; phylum: EchinodermataEchinodermata; class: AsteroideaAsteroidea; order: VelatidaVelatida; family: PterasteridaePterasteridae; genus: HymenasterHymenaster; taxonRank: genus; scientificNameAuthorship: Thomson, 1873; **Location:** waterBody: Pacific Ocean; stateProvince: Clarion-Clipperton Zone; locality: UK Seabed Resources Ltd exploration contract area (UK-1); verbatimLocality: UK-1 Stratum A; maximumDepthInMeters: 4022; locationRemarks: RV Melville Cruise MV1313; decimalLatitude: 13.8577; decimalLongitude: -116.5479; geodeticDatum: WGS84; coordinateUncertaintyInMeters: 25; **Identification:** identifiedBy: Christopher Mah, Diva J Amon, Amanda F Ziegler; dateIdentified: 2014; identificationRemarks: Identified only from imagery; identificationQualifier: cf.; **Event:** samplingProtocol: Remotely Operated Vehicle; eventDate: 2013-10-21; eventTime: 3:46; habitat: Abyssal polymetallic-nodule field; fieldNumber: Dive 6 (RV06); **Record Level:** language: en; institutionCode: UHM; datasetName: ABYSSLINE; basisOfRecord: HumanObservation

##### Notes

Fig. 14

#### Crinoidea

#### Comatulida

Sieverts-Doreck, 1953

#### cf. Comatulida morphospecies 1

cf. Comatulida morphospecies 1 In the “Atlas of Abyssal Megafauna Morphotypes of the Clarion-Clipperton Fracture Zone” created for the ISA (http://ccfzatlas.com/), this morphospecies is listed as "Featherstar sp. 1".

##### Materials

- **Type status:** Other material. **Occurrence:** recordedBy: Diva J Amon, Amanda F Ziegler; individualCount: 1; lifeStage: Adult; behavior: On nodule; occurrenceStatus: present; preparations: Imaged only; associatedReferences: 10.1038/srep30492Amon DJ, Ziegler AF, Dahlgren TG, Glover AG, Goineau A, Gooday AJ, Wiklund H, Smith CR. Insights into the abundance and diversity of abyssal megafauna in a polymetallic-nodule region in the eastern Clarion-Clipperton Zone. Scientific Reports. 2016;6. doi: 10.1038/srep30492; **Taxon:** taxonConceptID: Comatulidacf. Comatulida morphospecies 1; scientificName: ComatulidaComatulida sp.; kingdom: AnimaliaAnimalia; phylum: EchinodermataEchinodermata; class: CrinoideaCrinoidea; order: ComatulidaComatulida; taxonRank: order; **Location:** waterBody: Pacific Ocean; stateProvince: Clarion-Clipperton Zone; locality: Eastern Clarion-Clipperton Zone; verbatimLocality: Site EPIRB; maximumDepthInMeters: 3914; locationRemarks: RV Melville Cruise MV1313; decimalLatitude: 13.6793; decimalLongitude: -114.4072; geodeticDatum: WGS84; coordinateUncertaintyInMeters: 25; **Identification:** identifiedBy: Michel Roux, Diva J Amon, Amanda F Ziegler; dateIdentified: 2014; identificationRemarks: Identified only from imagery; identificationQualifier: cf.; **Event:** samplingProtocol: Remotely Operated Vehicle; eventDate: 2013-10-23; eventTime: 11:52; habitat: Abyssal polymetallic-nodule field; fieldNumber: Dive 7 (RV07); **Record Level:** language: en; institutionCode: UHM; datasetName: ABYSSLINE; basisOfRecord: HumanObservation
- **Type status:** Other material. **Occurrence:** recordedBy: Diva J Amon, Amanda F Ziegler; individualCount: 1; lifeStage: Adult; behavior: On nodule; occurrenceStatus: present; preparations: Imaged only; associatedReferences: 10.1038/srep30492Amon DJ, Ziegler AF, Dahlgren TG, Glover AG, Goineau A, Gooday AJ, Wiklund H, Smith CR. Insights into the abundance and diversity of abyssal megafauna in a polymetallic-nodule region in the eastern Clarion-Clipperton Zone. Scientific Reports. 2016;6. doi: 10.1038/srep30492; **Taxon:** taxonConceptID: Comatulidacf. Comatulida morphospecies 1; scientificName: ComatulidaComatulida sp.; kingdom: AnimaliaAnimalia; phylum: EchinodermataEchinodermata; class: CrinoideaCrinoidea; order: ComatulidaComatulida; taxonRank: order; **Location:** waterBody: Pacific Ocean; stateProvince: Clarion-Clipperton Zone; locality: UK Seabed Resources Ltd exploration contract area (UK-1); verbatimLocality: UK-1 Stratum A; maximumDepthInMeters: 4025; locationRemarks: RV Melville Cruise MV1313; decimalLatitude: 13.8639; decimalLongitude: -116.5487; geodeticDatum: WGS84; coordinateUncertaintyInMeters: 25; **Identification:** identifiedBy: Michel Roux, Diva J Amon, Amanda F Ziegler; dateIdentified: 2014; identificationRemarks: Identified only from imagery; identificationQualifier: cf.; **Event:** samplingProtocol: Remotely Operated Vehicle; eventDate: 2013-10-21; eventTime: 5:14; habitat: Abyssal polymetallic-nodule field; fieldNumber: Dive 6 (RV06); **Record Level:** language: en; institutionCode: UHM; datasetName: ABYSSLINE; basisOfRecord: HumanObservation

##### Notes

Fig. 15

#### cf. Comatulida morphospecies 2

cf. Comatulida morphospecies 2 In the “Atlas of Abyssal Megafauna Morphotypes of the Clarion-Clipperton Fracture Zone” created for the ISA (http://ccfzatlas.com/), this morphospecies is listed as "*Pentametrocrinus* sp. 1".

##### Materials

- **Type status:** Other material. **Occurrence:** recordedBy: Diva J Amon, Amanda F Ziegler; individualCount: 1; lifeStage: Adult; behavior: On nodule; occurrenceStatus: present; preparations: Imaged only; associatedReferences: 10.1038/srep30492Amon DJ, Ziegler AF, Dahlgren TG, Glover AG, Goineau A, Gooday AJ, Wiklund H, Smith CR. Insights into the abundance and diversity of abyssal megafauna in a polymetallic-nodule region in the eastern Clarion-Clipperton Zone. Scientific Reports. 2016;6. doi: 10.1038/srep30492; **Taxon:** taxonConceptID: Comatulidacf. Comatulida morphospecies 2; scientificName: ComatulidaComatulida sp.; kingdom: AnimaliaAnimalia; phylum: EchinodermataEchinodermata; class: CrinoideaCrinoidea; order: ComatulidaComatulida; taxonRank: order; **Location:** waterBody: Pacific Ocean; stateProvince: Clarion-Clipperton Zone; locality: UK Seabed Resources Ltd exploration contract area (UK-1); verbatimLocality: UK-1 Stratum A; maximumDepthInMeters: 4110; locationRemarks: RV Melville Cruise MV1313; decimalLatitude: 13.8498; decimalLongitude: -116.6458; geodeticDatum: WGS84; coordinateUncertaintyInMeters: 25; **Identification:** identifiedBy: Michel Roux, Diva J Amon, Amanda F Ziegler; dateIdentified: 2014; identificationRemarks: Identified only from imagery; identificationQualifier: cf.; **Event:** samplingProtocol: Remotely Operated Vehicle; eventDate: 2013-10-10; eventTime: 13:48; habitat: Abyssal polymetallic-nodule field; fieldNumber: Dive 1 (RV01); **Record Level:** language: en; institutionCode: UHM; datasetName: ABYSSLINE; basisOfRecord: HumanObservation

##### Notes

Fig. 16

#### Bathycrinidae

Bather, 1899

#### Bathycrinus

Thomson, 1872

#### Bathycrinus cf. equatorialis

A.H. Clark, 1908

Bathycrinus cf. equatorialis In the “Atlas of Abyssal Megafauna Morphotypes of the Clarion-Clipperton Fracture Zone” created for the ISA (http://ccfzatlas.com/), this morphospecies is listed as "Bourgueticrinina sp. 1".

##### Materials

- **Type status:** Other material. **Occurrence:** recordedBy: Diva J Amon, Amanda F Ziegler; individualCount: 1; lifeStage: Adult; behavior: On nodule; occurrenceStatus: present; preparations: Imaged only; associatedReferences: 10.1038/srep30492.Amon DJ, Ziegler AF, Dahlgren TG, Glover AG, Goineau A, Gooday AJ, Wiklund H, Smith CR. Insights into the abundance and diversity of abyssal megafauna in a polymetallic-nodule region in the eastern Clarion-Clipperton Zone. Scientific Reports. 2016;6. doi: 10.1038/srep30492.; **Taxon:** taxonConceptID: Bathycrinus cf. equatorialisBathycrinus cf. equatorialis; scientificName: Bathycrinus equatorialisBathycrinus equatorialis; kingdom: AnimaliaAnimalia; phylum: EchinodermataEchinodermata; class: CrinoideaCrinoidea; order: ComatulidaComatulida; family: BathycrinidaeBathycrinidae; genus: BathycrinusBathycrinus; taxonRank: species; scientificNameAuthorship: AH Clark, 1908; **Location:** waterBody: Pacific Ocean; stateProvince: Clarion-Clipperton Zone; locality: UK Seabed Resources Ltd exploration contract area (UK-1); verbatimLocality: UK-1 Stratum A; maximumDepthInMeters: 4071; locationRemarks: RV Melville Cruise MV1313; decimalLatitude: 13.7605; decimalLongitude: -116.468; geodeticDatum: WGS84; coordinateUncertaintyInMeters: 25; **Identification:** identifiedBy: Michel Roux, Diva J Amon, Amanda F Ziegler; dateIdentified: 2014; identificationRemarks: Identified only from imagery; identificationQualifier: cf.; **Event:** samplingProtocol: Remotely Operated Vehicle; eventDate: 2013-10-18; eventTime: 2:14; habitat: Abyssal polymetallic-nodule field; fieldNumber: Dive 5 (RV05); **Record Level:** language: en; institutionCode: UHM; datasetName: ABYSSLINE; basisOfRecord: HumanObservation
- **Type status:** Other material. **Occurrence:** recordedBy: Diva J Amon, Amanda F Ziegler; individualCount: 1; lifeStage: Adult; behavior: On nodule; occurrenceStatus: present; preparations: Imaged only; associatedReferences: 10.1038/srep30492.Amon DJ, Ziegler AF, Dahlgren TG, Glover AG, Goineau A, Gooday AJ, Wiklund H, Smith CR. Insights into the abundance and diversity of abyssal megafauna in a polymetallic-nodule region in the eastern Clarion-Clipperton Zone. Scientific Reports. 2016;6. doi: 10.1038/srep30492.; **Taxon:** taxonConceptID: Bathycrinus cf. equatorialisBathycrinus cf. equatorialis; scientificName: Bathycrinus equatorialisBathycrinus equatorialis; kingdom: AnimaliaAnimalia; phylum: EchinodermataEchinodermata; class: CrinoideaCrinoidea; order: Comatulida Comatulida ; family: BathycrinidaeBathycrinidae; genus: BathycrinusBathycrinus; taxonRank: species; scientificNameAuthorship: AH Clark, 1908; **Location:** waterBody: Pacific Ocean; stateProvince: Clarion-Clipperton Zone; locality: Eastern Clarion-Clipperton Zone; verbatimLocality: Site EPIRB; maximumDepthInMeters: 3909; locationRemarks: RV Melville Cruise MV1313; decimalLatitude: 13.6785; decimalLongitude: -114.406; geodeticDatum: WGS84; coordinateUncertaintyInMeters: 25; **Identification:** identifiedBy: Michel Roux, Diva J Amon, Amanda F Ziegler; dateIdentified: 2014; identificationRemarks: Identified only from imagery; identificationQualifier: cf.; **Event:** samplingProtocol: Remotely Operated Vehicle; eventDate: 2013-10-23; eventTime: 11:21; habitat: Abyssal polymetallic-nodule field; fieldNumber: Dive 7 (RV07); **Record Level:** language: en; institutionCode: UHM; datasetName: ABYSSLINE; basisOfRecord: HumanObservation

##### Notes

Fig. 17

#### Hyocrinida

Rasmussen, 1978

#### Hyocrinidae

Carpenter, 1884

#### cf. Hyocrinidae morphospecies

cf. Hyocrinidae morphospecies In the “Atlas of Abyssal Megafauna Morphotypes of the Clarion-Clipperton Fracture Zone” created for the ISA (http://ccfzatlas.com/), this morphospecies is listed as "Bourgueticrinina sp. 1".

##### Materials

- **Type status:** Other material. **Occurrence:** recordedBy: Diva J Amon, Amanda F Ziegler; individualCount: 1; lifeStage: Adult; behavior: On nodule; occurrenceStatus: present; preparations: Imaged only; associatedReferences: 10.1038/srep30492Amon DJ, Ziegler AF, Dahlgren TG, Glover AG, Goineau A, Gooday AJ, Wiklund H, Smith CR. Insights into the abundance and diversity of abyssal megafauna in a polymetallic-nodule region in the eastern Clarion-Clipperton Zone. Scientific Reports. 2016;6. doi: 10.1038/srep30492; **Taxon:** taxonConceptID: Hyocrinidaecf. Hyocrinidae morphospecies; scientificName: HyocrinidaeHyocrinidae sp.; kingdom: AnimaliaAnimalia; phylum: EchinodermataEchinodermata; class: CrinoideaCrinoidea; order: HyocrinidaHyocrinida; family: HyocrinidaeHyocrinidae; taxonRank: family; scientificNameAuthorship: Carpenter, 1884; **Location:** waterBody: Pacific Ocean; stateProvince: Clarion-Clipperton Zone; locality: UK Seabed Resources Ltd exploration contract area (UK-1); verbatimLocality: UK-1 Stratum A; maximumDepthInMeters: 3919; locationRemarks: RV Melville Cruise MV1313; decimalLatitude: 13.6787; decimalLongitude: -114.4072; geodeticDatum: WGS84; coordinateUncertaintyInMeters: 25; **Identification:** identifiedBy: Michel Roux, Diva J Amon, Amanda F Ziegler; dateIdentified: 2014; identificationRemarks: Identified only from imagery; identificationQualifier: cf.; **Event:** samplingProtocol: Remotely Operated Vehicle; eventDate: 2013-10-23; eventTime: 11:09; habitat: Abyssal polymetallic-nodule field; fieldNumber: Dive 7 (RV07); **Record Level:** language: en; institutionCode: UHM; datasetName: ABYSSLINE; basisOfRecord: HumanObservation

##### Notes

Fig. 18

#### Hyocrinus

Thomson, 1876

#### Hyocrinus cf. foelli

Roux & Pawson, 1999

Hyocrinus cf. foelli In the “Atlas of Abyssal Megafauna Morphotypes of the Clarion-Clipperton Fracture Zone” created for the ISA (http://ccfzatlas.com/), this morphospecies is listed as "Hyocrinidae sp. 1".

##### Materials

- **Type status:** Other material. **Occurrence:** recordedBy: Diva J Amon, Amanda F Ziegler; individualCount: 1; lifeStage: Adult; behavior: On nodule; occurrenceStatus: present; preparations: Imaged only; associatedReferences: 10.1038/srep30492.Amon DJ, Ziegler AF, Dahlgren TG, Glover AG, Goineau A, Gooday AJ, Wiklund H, Smith CR. Insights into the abundance and diversity of abyssal megafauna in a polymetallic-nodule region in the eastern Clarion-Clipperton Zone. Scientific Reports. 2016, 6. doi: 10.1038/srep30492.; **Taxon:** taxonConceptID: Hyocrinus cf. foelliHyocrinus cf. foelli; scientificName: Hyocrinus foelliHyocrinus foelli; kingdom: AnimaliaAnimalia; phylum: EchinodermataEchinodermata; class: CrinoideaCrinoidea; order: HyocrinidaHyocrinida; family: HyocrinidaeHyocrinidae; genus: HyocrinusHyocrinus; taxonRank: species; scientificNameAuthorship: Roux & Pawson, 1999; **Location:** waterBody: Pacific Ocean; stateProvince: Clarion-Clipperton Zone; locality: Eastern Clarion-Clipperton Zone; verbatimLocality: Site EPIRB; maximumDepthInMeters: 3944; locationRemarks: RV Melville Cruise MV1313; decimalLatitude: 13.6794; decimalLongitude: -114.41297; geodeticDatum: WGS84; coordinateUncertaintyInMeters: 25; **Identification:** identifiedBy: Michel Roux, Diva J Amon, Amanda F Ziegler; dateIdentified: 2014; identificationReferences: EchinodermataEchinodermataRoux M & Pawson DL. Two New Pacific Ocean Species of Hyocrinid Crinoids (Echinodermata),with Comments on Presumed Giant-Dwarf Gradients Related to Seamountsand Abyssal Plains. Pacific Science. 1999, 53(3), 289-298; Roux M (2004). New hyocrinid crinoids (Echinodermata) from submersible investigations in the Pacific Ocean. Pacific Science, 58:597-613.; identificationRemarks: Identified only from imagery; identificationQualifier: cf.; **Event:** samplingProtocol: Remotely Operated Vehicle; eventDate: 2013-10-23; eventTime: 10:06; habitat: Abyssal polymetallic-nodule field; fieldNumber: Dive 7 (RV07); **Record Level:** language: en; institutionCode: UHM; datasetName: ABYSSLINE; basisOfRecord: HumanObservation
- **Type status:** Other material. **Occurrence:** recordedBy: Diva J Amon, Amanda F Ziegler; individualCount: 1; lifeStage: Adult; behavior: On nodule; occurrenceStatus: present; preparations: Imaged only; associatedReferences: 10.1038/srep30492Amon DJ, Ziegler AF, Dahlgren TG, Glover AG, Goineau A, Gooday AJ, Wiklund H, Smith CR. Insights into the abundance and diversity of abyssal megafauna in a polymetallic-nodule region in the eastern Clarion-Clipperton Zone. Scientific Reports. 2016, 6. doi: 10.1038/srep30492; **Taxon:** taxonConceptID: Hyocrinus cf. foelliHyocrinus cf. foelli; scientificName: Hyocrinus foelliHyocrinus foelli; kingdom: AnimaliaAnimalia; phylum: EchinodermataEchinodermata; class: CrinoideaCrinoidea; order: HyocrinidaHyocrinida; family: HyocrinidaeHyocrinidae; genus: HyocrinusHyocrinus; taxonRank: species; scientificNameAuthorship: Roux & Pawson, 1999; **Location:** waterBody: Pacific Ocean; stateProvince: Clarion-Clipperton Zone; locality: Eastern Clarion-Clipperton Zone; verbatimLocality: Site EPIRB; maximumDepthInMeters: 3919; locationRemarks: RV Melville Cruise MV1313; decimalLatitude: 13.6792; decimalLongitude: -114.4079; geodeticDatum: WGS84; coordinateUncertaintyInMeters: 25; **Identification:** identifiedBy: Michel Roux, Diva J Amon, Amanda F Ziegler; dateIdentified: 2014; identificationReferences: EchinodermataEchinodermataRoux M & Pawson DL. Two New Pacific Ocean Species of Hyocrinid Crinoids (Echinodermata),with Comments on Presumed Giant-Dwarf Gradients Related to Seamountsand Abyssal Plains. Pacific Science. 1999, 53(3), 289-298; Roux M (2004). New hyocrinid crinoids (Echinodermata) from submersible investigations in the Pacific Ocean. Pacific Science, 58:597-613.; identificationRemarks: Identified only from imagery; identificationQualifier: cf.; **Event:** samplingProtocol: Remotely Operated Vehicle; eventDate: 2013-10-23; eventTime: 11:59; habitat: Abyssal polymetallic-nodule field; fieldNumber: Dive 7 (RV07); **Record Level:** language: en; institutionCode: UHM; datasetName: ABYSSLINE; basisOfRecord: HumanObservation

##### Notes

Fig. 19

#### Echinoidea

Leske, 1778

#### cf. Echinoidea morphospecies 1

##### Materials

- **Type status:** Other material. **Occurrence:** recordedBy: Diva J Amon, Amanda F Ziegler; individualCount: 1; lifeStage: Adult; behavior: On seafloor; occurrenceStatus: present; preparations: Imaged only; **Taxon:** taxonConceptID: Echinoideacf. Echinoidea morphospecies 1; scientificName: EchinoideaEchinoidea sp.; kingdom: AnimaliaAnimalia; phylum: EchinodermataEchinodermata; class: EchinoideaEchinoidea; taxonRank: class; **Location:** waterBody: Pacific Ocean; stateProvince: Clarion-Clipperton Zone; locality: UK Seabed Resources Ltd exploration contract area (UK-1); verbatimLocality: UK-1 Stratum B; maximumDepthInMeters: 4163; locationRemarks: RV Thompson Cruise TN319; decimalLatitude: 12.369004; decimalLongitude: -116.5195; geodeticDatum: WGS84; coordinateUncertaintyInMeters: 25; **Identification:** identifiedBy: Richard Mooi, Diva J Amon, Amanda F Ziegler; dateIdentified: 2015; identificationRemarks: Identified only from imagery; identificationQualifier: cf.; **Event:** samplingProtocol: Autonomous Underwater Vehicle; eventDate: 2015-02-18; eventTime: 16:29; habitat: Abyssal polymetallic-nodule field; fieldNumber: Dive 1 (AV01); **Record Level:** language: en; institutionCode: UHM; datasetName: ABYSSLINE; basisOfRecord: HumanObservation
- **Type status:** Other material. **Occurrence:** recordedBy: Diva J Amon, Amanda F Ziegler; individualCount: 1; lifeStage: Adult; behavior: On seafloor; occurrenceStatus: present; preparations: Imaged only; **Taxon:** taxonConceptID: Echinoideacf. Echinoidea morphospecies 1; scientificName: EchinoideaEchinoidea sp.; kingdom: AnimaliaAnimalia; phylum: EchinodermataEchinodermata; class: EchinoideaEchinoidea; taxonRank: class; **Location:** waterBody: Pacific Ocean; stateProvince: Clarion-Clipperton Zone; locality: UK Seabed Resources Ltd exploration contract area (UK-1); verbatimLocality: UK-1 Stratum B; maximumDepthInMeters: 4253; locationRemarks: RV Thompson Cruise TN319; decimalLatitude: 12.4963; decimalLongitude: -116.64899; geodeticDatum: WGS84; coordinateUncertaintyInMeters: 25; **Identification:** identifiedBy: Richard Mooi, Diva J Amon, Amanda F Ziegler; dateIdentified: 2015; identificationRemarks: Identified only from imagery; identificationQualifier: cf.; **Event:** samplingProtocol: Autonomous Underwater Vehicle; eventDate: 2015-03-04; eventTime: 0:04; habitat: Abyssal polymetallic-nodule field; fieldNumber: Dive 5 (AV05); **Record Level:** language: en; institutionCode: UHM; datasetName: ABYSSLINE; basisOfRecord: HumanObservation

##### Notes

Fig. 20

#### cf. Echinoidea morphospecies 2

##### Materials

- **Type status:** Other material. **Occurrence:** recordedBy: Diva J Amon, Amanda F Ziegler; individualCount: 1; lifeStage: Adult; behavior: On seafloor; occurrenceStatus: present; preparations: Imaged only; **Taxon:** taxonConceptID: Echinoideacf. Echinoidea morphospecies 2; scientificName: EchinoideaEchinoidea sp.; kingdom: AnimaliaAnimalia; phylum: EchinodermataEchinodermata; class: EchinoideaEchinoidea; taxonRank: class; **Location:** waterBody: Pacific Ocean; stateProvince: Clarion-Clipperton Zone; locality: UK Seabed Resources Ltd exploration contract area (UK-1); verbatimLocality: UK-1 Stratum B; maximumDepthInMeters: 4251; locationRemarks: RV Thompson Cruise TN319; decimalLatitude: 12.5014; decimalLongitude: -116.64696; geodeticDatum: WGS84; coordinateUncertaintyInMeters: 25; **Identification:** identifiedBy: Richard Mooi, Diva J Amon, Amanda F Ziegler; dateIdentified: 2015; identificationRemarks: Identified only from imagery; identificationQualifier: cf.; **Event:** samplingProtocol: Autonomous Underwater Vehicle; eventDate: 2015-03-03; eventTime: 21:44; habitat: Abyssal polymetallic-nodule field; fieldNumber: Dive 5 (AV05); **Record Level:** language: en; institutionCode: UHM; datasetName: ABYSSLINE; basisOfRecord: HumanObservation

##### Notes

Fig. 21

#### Aspidodiadematoida

Kroh & Smith, 2010

#### Aspidodiadematidae

Duncan 1889

#### cf. Aspidodiadematidae morphospecies

##### Materials

- **Type status:** Other material. **Occurrence:** recordedBy: Diva J Amon, Amanda F Ziegler; individualCount: 1; lifeStage: Adult; behavior: On seafloor; occurrenceStatus: present; preparations: Imaged only; **Taxon:** taxonConceptID: Aspidodiadematidaecf. Aspidodiadematidae morphospecies; scientificName: AspidodiadematidaeAspidodiadematidae sp.; kingdom: AnimaliaAnimalia; phylum: EchinodermataEchinodermata; class: EchinoideaEchinoidea; order: AspidodiadematoidaAspidodiadematoida; family: AspidodiadematidaeAspidodiadematidae; taxonRank: family; scientificNameAuthorship: Duncan 1889; **Location:** waterBody: Pacific Ocean; stateProvince: Clarion-Clipperton Zone; locality: UK Seabed Resources Ltd exploration contract area (UK-1); verbatimLocality: UK-1 Stratum B; maximumDepthInMeters: 4255; locationRemarks: RV Thompson Cruise TN319; decimalLatitude: 12.5025; decimalLongitude: -116.6489; geodeticDatum: WGS84; coordinateUncertaintyInMeters: 25; **Identification:** identifiedBy: Richard Mooi, Diva J Amon, Amanda F Ziegler; dateIdentified: 2015; identificationRemarks: Identified only from imagery; identificationQualifier: cf.; **Event:** samplingProtocol: Autonomous Underwater Vehicle; eventDate: 2015-03-18; eventTime: 8:35; habitat: Abyssal polymetallic-nodule field; fieldNumber: Dive 9 (AV09); **Record Level:** language: en; institutionCode: UHM; datasetName: ABYSSLINE; basisOfRecord: HumanObservation
- **Type status:** Other material. **Occurrence:** recordedBy: Diva J Amon, Amanda F Ziegler; individualCount: 1; lifeStage: Adult; behavior: On seafloor; occurrenceStatus: present; preparations: Imaged only; associatedReferences: 10.1038/srep30492Amon DJ, Ziegler AF, Dahlgren TG, Glover AG, Goineau A, Gooday AJ, Wiklund H, Smith CR. Insights into the abundance and diversity of abyssal megafauna in a polymetallic-nodule region in the eastern Clarion-Clipperton Zone. Scientific Reports. 2016;6. doi: 10.1038/srep30492; **Taxon:** taxonConceptID: Aspidodiadematidaecf. Aspidodiadematidae morphospecies; scientificName: AspidodiadematidaeAspidodiadematidae sp.; kingdom: AnimaliaAnimalia; phylum: EchinodermataEchinodermata; class: EchinoideaEchinoidea; order: AspidodiadematoidaAspidodiadematoida; family: AspidodiadematidaeAspidodiadematidae; taxonRank: family; scientificNameAuthorship: Duncan 1889; **Location:** waterBody: Pacific Ocean; stateProvince: Clarion-Clipperton Zone; locality: UK Seabed Resources Ltd exploration contract area (UK-1); verbatimLocality: UK-1 Stratum A; maximumDepthInMeters: 4108; locationRemarks: RV Melville Cruise MV1313; decimalLatitude: 13.8502; decimalLongitude: -116.6457; geodeticDatum: WGS84; coordinateUncertaintyInMeters: 25; **Identification:** identifiedBy: Richard Mooi, Diva J Amon, Amanda F Ziegler; dateIdentified: 2014; identificationRemarks: Identified only from imagery; identificationQualifier: cf.; **Event:** samplingProtocol: Remotely Operated Vehicle; eventDate: 2013-10-10; eventTime: 12:05; habitat: Abyssal polymetallic-nodule field; fieldNumber: Dive 1 (RV01); **Record Level:** language: en; institutionCode: UHM; datasetName: ABYSSLINE; basisOfRecord: HumanObservation

##### Notes

Fig. 22

#### Holasteroida

Durham & Melville, 1957

#### cf. Holasteroida morphospecies 1

##### Materials

- **Type status:** Other material. **Occurrence:** recordedBy: Diva J Amon, Amanda F Ziegler; individualCount: 1; lifeStage: Adult; behavior: On seafloor; occurrenceStatus: present; preparations: Imaged only; **Taxon:** taxonConceptID: Holasteroidacf. Holasteroida morphospecies 1; scientificName: HolasteroidaHolasteroida sp.; kingdom: AnimaliaAnimalia; phylum: EchinodermataEchinodermata; class: EchinoideaEchinoidea; order: HolasteroidaHolasteroida; taxonRank: order; scientificNameAuthorship: Durham & Melville, 1957; **Location:** waterBody: Pacific Ocean; stateProvince: Clarion-Clipperton Zone; locality: UK Seabed Resources Ltd exploration contract area (UK-1); verbatimLocality: UK-1 Stratum B; maximumDepthInMeters: 4224; locationRemarks: RV Thompson Cruise TN319; decimalLatitude: 12.5797; decimalLongitude: -116.7271; geodeticDatum: WGS84; coordinateUncertaintyInMeters: 25; **Identification:** identifiedBy: Richard Mooi, Diva J Amon, Amanda F Ziegler; dateIdentified: 2015; identificationRemarks: Identified only from imagery; identificationQualifier: cf.; **Event:** samplingProtocol: Autonomous Underwater Vehicle; eventDate: 2015-03-09; eventTime: 7:56; habitat: Abyssal polymetallic-nodule field; fieldNumber: Dive 6 (AV06); **Record Level:** language: en; institutionCode: UHM; datasetName: ABYSSLINE; basisOfRecord: HumanObservation

##### Notes

Fig. 23

#### cf. Holasteroida morphospecies 2

##### Materials

- **Type status:** Other material. **Occurrence:** recordedBy: Diva J Amon, Amanda F Ziegler; individualCount: 1; lifeStage: Adult; behavior: On seafloor; occurrenceStatus: present; preparations: Imaged only; **Taxon:** taxonConceptID: Holasteroidacf. Holasteroida morphospecies 2; scientificName: HolasteroidaHolasteroida sp.; kingdom: AnimaliaAnimalia; phylum: EchinodermataEchinodermata; class: EchinoideaEchinoidea; order: HolasteroidaHolasteroida; taxonRank: order; scientificNameAuthorship: Durham & Melville, 1957; **Location:** waterBody: Pacific Ocean; stateProvince: Clarion-Clipperton Zone; locality: UK Seabed Resources Ltd exploration contract area (UK-1); verbatimLocality: UK-1 Stratum B; maximumDepthInMeters: 4253; locationRemarks: RV Thompson Cruise TN319; decimalLatitude: 12.4965; decimalLongitude: -116.64997; geodeticDatum: WGS84; coordinateUncertaintyInMeters: 25; **Identification:** identifiedBy: Richard Mooi, Diva J Amon, Amanda F Ziegler; dateIdentified: 2015; identificationRemarks: Identified only from imagery; identificationQualifier: cf.; **Event:** samplingProtocol: Autonomous Underwater Vehicle; eventDate: 2015-03-04; eventTime: 6:59; habitat: Abyssal polymetallic-nodule field; fieldNumber: Dive 5 (AV05); **Record Level:** language: en; institutionCode: UHM; datasetName: ABYSSLINE; basisOfRecord: HumanObservation
- **Type status:** Other material. **Occurrence:** recordedBy: Diva J Amon, Amanda F Ziegler; individualCount: 1; lifeStage: Adult; behavior: On seafloor; occurrenceStatus: present; preparations: Imaged only; **Taxon:** taxonConceptID: Holasteroidacf. Holasteroida morphospecies 2; scientificName: HolasteroidaHolasteroida sp.; kingdom: AnimaliaAnimalia; phylum: EchinodermataEchinodermata; class: EchinoideaEchinoidea; order: HolasteroidaHolasteroida; taxonRank: order; scientificNameAuthorship: Durham & Melville, 1957; **Location:** waterBody: Pacific Ocean; stateProvince: Clarion-Clipperton Zone; locality: UK Seabed Resources Ltd exploration contract area (UK-1); verbatimLocality: UK-1 Stratum B; maximumDepthInMeters: 4227; locationRemarks: RV Thompson Cruise TN319; decimalLatitude: 12.5796; decimalLongitude: -116.7233; geodeticDatum: WGS84; coordinateUncertaintyInMeters: 25; **Identification:** identifiedBy: Richard Mooi, Diva J Amon, Amanda F Ziegler; dateIdentified: 2015; identificationRemarks: Identified only from imagery; identificationQualifier: cf.; **Event:** samplingProtocol: Autonomous Underwater Vehicle; eventDate: 2015-03-09; eventTime: 11:03; habitat: Abyssal polymetallic-nodule field; fieldNumber: Dive 6 (AV06); **Record Level:** language: en; institutionCode: UHM; datasetName: ABYSSLINE; basisOfRecord: HumanObservation

##### Notes

Fig. 24

#### Pourtalesiidae

A. Agassiz, 1881

#### Cystocrepis

Mortensen, 1907

#### cf. Cystocrepis morphospecies

##### Materials

- **Type status:** Other material. **Occurrence:** recordedBy: Diva J Amon, Amanda F Ziegler; individualCount: 1; lifeStage: Adult; behavior: On seafloor; occurrenceStatus: present; preparations: Imaged only; associatedReferences: 10.1038/srep30492Amon DJ, Ziegler AF, Dahlgren TG, Glover AG, Goineau A, Gooday AJ, Wiklund H, Smith CR. Insights into the abundance and diversity of abyssal megafauna in a polymetallic-nodule region in the eastern Clarion-Clipperton Zone. Scientific Reports. 2016;6. doi: 10.1038/srep30492; **Taxon:** taxonConceptID: *Cystocrepis*cf. *Cystocrepis* morphospecies; scientificName: *CystocrepisCystocrepis* sp.; kingdom: AnimaliaAnimalia; phylum: EchinodermataEchinodermata; class: EchinoideaEchinoidea; order: HolasteroidaHolasteroida; family: PourtalesiidaePourtalesiidae; genus: CystocrepisCystocrepis; taxonRank: genus; scientificNameAuthorship: Mortensen, 1907; **Location:** waterBody: Pacific Ocean; stateProvince: Clarion-Clipperton Zone; locality: UK Seabed Resources Ltd exploration contract area (UK-1); verbatimLocality: UK-1 Stratum A; maximumDepthInMeters: 4022; locationRemarks: RV Melville Cruise MV1313; decimalLatitude: 13.85801; decimalLongitude: -116.5473; geodeticDatum: WGS84; coordinateUncertaintyInMeters: 25; **Identification:** identifiedBy: Richard Mooi, Diva J Amon, Amanda F Ziegler; dateIdentified: 2014; identificationRemarks: Identified only from imagery; identificationQualifier: cf.; **Event:** samplingProtocol: Remotely Operated Vehicle; eventDate: 2013-10-21; eventTime: 2:33; habitat: Abyssal polymetallic-nodule field; fieldNumber: Dive 6 (RV06); **Record Level:** language: en; institutionCode: UHM; datasetName: ABYSSLINE; basisOfRecord: HumanObservation

##### Notes

Fig. 25

#### Echinocrepis

A. Agassiz, 1879

#### cf. Echinocrepis morphospecies

##### Materials

- **Type status:** Other material. **Occurrence:** recordedBy: Diva J Amon, Amanda F Ziegler; individualCount: 1; lifeStage: Adult; behavior: On seafloor; occurrenceStatus: present; preparations: Imaged only; associatedReferences: 10.1038/srep30492Amon DJ, Ziegler AF, Dahlgren TG, Glover AG, Goineau A, Gooday AJ, Wiklund H, Smith CR. Insights into the abundance and diversity of abyssal megafauna in a polymetallic-nodule region in the eastern Clarion-Clipperton Zone. Scientific Reports. 2016;6. doi: 10.1038/srep30492; **Taxon:** taxonConceptID: *Echinocrepis*cf. *Echinocrepis* morphospecies; scientificName: *EchinocrepisEchinocrepis* sp.; kingdom: AnimaliaAnimalia; phylum: EchinodermataEchinodermata; class: EchinoideaEchinoidea; order: HolasteroidaHolasteroida; family: PourtalesiidaePourtalesiidae; genus: EchinocrepisEchinocrepis; taxonRank: genus; scientificNameAuthorship: A Agassiz, 1879; **Location:** waterBody: Pacific Ocean; stateProvince: Clarion-Clipperton Zone; locality: UK Seabed Resources Ltd exploration contract area (UK-1); verbatimLocality: UK-1 Stratum A; maximumDepthInMeters: 4026; locationRemarks: RV Melville Cruise MV1313; decimalLatitude: 13.8601; decimalLongitude: -116.5484; geodeticDatum: WGS84; coordinateUncertaintyInMeters: 25; **Identification:** identifiedBy: Richard Mooi, Diva J Amon, Amanda F Ziegler; dateIdentified: 2014; identificationRemarks: Identified only from imagery; identificationQualifier: cf.; **Event:** samplingProtocol: Remotely Operated Vehicle; eventDate: 2013-10-21; eventTime: 4:22; habitat: Abyssal polymetallic-nodule field; fieldNumber: Dive 6 (RV06); **Record Level:** language: en; institutionCode: UHM; datasetName: ABYSSLINE; basisOfRecord: HumanObservation
- **Type status:** Other material. **Occurrence:** recordedBy: Diva J Amon, Amanda F Ziegler; individualCount: 1; lifeStage: Adult; behavior: On seafloor; occurrenceStatus: present; preparations: Imaged only; **Taxon:** taxonConceptID: *Echinocrepis*cf. *Echinocrepis* morphospecies; scientificName: *EchinocrepisEchinocrepis* sp.; kingdom: AnimaliaAnimalia; phylum: EchinodermataEchinodermata; class: EchinoideaEchinoidea; order: HolasteroidaHolasteroida; family: PourtalesiidaePourtalesiidae; genus: EchinocrepisEchinocrepis; taxonRank: genus; scientificNameAuthorship: A. Agassiz, 1879; **Location:** waterBody: Pacific Ocean; stateProvince: Clarion-Clipperton Zone; locality: UK Seabed Resources Ltd exploration contract area (UK-1); verbatimLocality: UK-1 Stratum B; maximumDepthInMeters: 4200; locationRemarks: RV Thompson Cruise TN319; decimalLatitude: 12.56795; decimalLongitude: -116.7361; geodeticDatum: WGS84; coordinateUncertaintyInMeters: 25; **Identification:** identifiedBy: Richard Mooi, Diva J Amon, Amanda F Ziegler; dateIdentified: 2015; identificationRemarks: Identified only from imagery; identificationQualifier: cf.; **Event:** samplingProtocol: Autonomous Underwater Vehicle; eventDate: 2015-03-09; eventTime: 10:36; habitat: Abyssal polymetallic-nodule field; fieldNumber: Dive 6 (AV06); **Record Level:** language: en; institutionCode: UHM; datasetName: ABYSSLINE; basisOfRecord: HumanObservation
- **Type status:** Other material. **Occurrence:** recordedBy: Diva J Amon, Amanda F Ziegler; individualCount: 1; lifeStage: Adult; behavior: On seafloor; occurrenceStatus: present; preparations: Imaged only; **Taxon:** taxonConceptID: *Echinocrepis*cf. *Echinocrepis* morphospecies; scientificName: *EchinocrepisEchinocrepis* sp.; kingdom: AnimaliaAnimalia; phylum: EchinodermataEchinodermata; class: EchinoideaEchinoidea; order: HolasteroidaHolasteroida; family: PourtalesiidaePourtalesiidae; genus: EchinocrepisEchinocrepis; taxonRank: genus; scientificNameAuthorship: A Agassiz, 1879; **Location:** waterBody: Pacific Ocean; stateProvince: Clarion-Clipperton Zone; locality: UK Seabed Resources Ltd exploration contract area (UK-1); verbatimLocality: UK-1 Stratum B; maximumDepthInMeters: 4213; locationRemarks: RV Thompson Cruise TN319; decimalLatitude: 12.5707; decimalLongitude: -116.7072; geodeticDatum: WGS84; coordinateUncertaintyInMeters: 25; **Identification:** identifiedBy: Richard Mooi, Diva J Amon, Amanda F Ziegler; dateIdentified: 2015; identificationRemarks: Identified only from imagery; identificationQualifier: cf.; **Event:** samplingProtocol: Autonomous Underwater Vehicle; eventDate: 2015-03-09; eventTime: 15:06; habitat: Abyssal polymetallic-nodule field; fieldNumber: Dive 6 (AV06); **Record Level:** language: en; institutionCode: UHM; datasetName: ABYSSLINE; basisOfRecord: HumanObservation

##### Notes

Fig. 26

#### Urechinidae

Duncan, 1889

#### cf. Urechinidae morphospecies

##### Materials

- **Type status:** Other material. **Occurrence:** recordedBy: Diva J Amon, Amanda F Ziegler; individualCount: 1; lifeStage: Adult; behavior: On seafloor; occurrenceStatus: present; preparations: Imaged only; associatedReferences: 10.1038/srep30492Amon DJ, Ziegler AF, Dahlgren TG, Glover AG, Goineau A, Gooday AJ, Wiklund H, Smith CR. Insights into the abundance and diversity of abyssal megafauna in a polymetallic-nodule region in the eastern Clarion-Clipperton Zone. Scientific Reports. 2016;6. doi: 10.1038/srep30492; **Taxon:** taxonConceptID: Urechinidaecf. Urechinidae morphospecies; scientificName: UrechinidaeUrechinidae sp.; kingdom: AnimaliaAnimalia; phylum: EchinodermataEchinodermata; class: EchinoideaEchinoidea; order: HolasteroidaHolasteroida; family: UrechinidaeUrechinidae; taxonRank: family; scientificNameAuthorship: Duncan, 1889; **Location:** waterBody: Pacific Ocean; stateProvince: Clarion-Clipperton Zone; locality: UK Seabed Resources Ltd exploration contract area (UK-1); verbatimLocality: UK-1 Stratum A; maximumDepthInMeters: 4020; locationRemarks: RV Melville Cruise MV1313; decimalLatitude: 13.8579; decimalLongitude: -116.54799; geodeticDatum: WGS84; coordinateUncertaintyInMeters: 25; **Identification:** identifiedBy: Richard Mooi, Diva J Amon, Amanda F Ziegler; dateIdentified: 2014; identificationRemarks: Identified only from imagery; identificationQualifier: cf.; **Event:** samplingProtocol: Remotely Operated Vehicle; eventDate: 2013-10-21; eventTime: 3:50; habitat: Abyssal polymetallic-nodule field; fieldNumber: Dive 6 (RV06); **Record Level:** language: en; institutionCode: UHM; datasetName: ABYSSLINE; basisOfRecord: HumanObservation

##### Notes

Fig. 27

#### Spatangoida

L. Agassiz, 1840

#### Schizasteridae

Lambert, 1905

#### Aceste

Thomson, 1877

#### Aceste cf. ovata

A. Agassiz, 1840

##### Materials

- **Type status:** Other material. **Occurrence:** recordedBy: Diva J Amon, Amanda F Ziegler; individualCount: 1; lifeStage: Adult; behavior: On seafloor; occurrenceStatus: present; preparations: Imaged only; associatedReferences: 10.1038/srep30492Amon DJ, Ziegler AF, Dahlgren TG, Glover AG, Goineau A, Gooday AJ, Wiklund H, Smith CR. Insights into the abundance and diversity of abyssal megafauna in a polymetallic-nodule region in the eastern Clarion-Clipperton Zone. Scientific Reports. 2016;6. doi: 10.1038/srep30492; **Taxon:** taxonConceptID: Aceste cf. ovataAceste cf. ovata; scientificName: Aceste ovataAceste ovata; kingdom: AnimaliaAnimalia; phylum: EchinodermataEchinodermata; class: EchinoideaEchinoidea; order: SpatangoidaSpatangoida; family: SchizasteridaeSchizasteridae; genus: AcesteAceste; taxonRank: species; scientificNameAuthorship: A Agassiz & HL Clark, 1907; **Location:** waterBody: Pacific Ocean; stateProvince: Clarion-Clipperton Zone; locality: UK Seabed Resources Ltd exploration contract area (UK-1); verbatimLocality: UK-1 Stratum A; maximumDepthInMeters: 4028; locationRemarks: RV Melville Cruise MV1313; decimalLatitude: 13.8619; decimalLongitude: -116.5484; geodeticDatum: WGS84; coordinateUncertaintyInMeters: 25; **Identification:** identifiedBy: Richard Mooi, Diva J Amon, Amanda F Ziegler; dateIdentified: 2014; identificationRemarks: Identified only from imagery; identificationQualifier: cf.; **Event:** samplingProtocol: Remotely Operated Vehicle; eventDate: 2013-10-21; eventTime: 4:47; habitat: Abyssal polymetallic-nodule field; fieldNumber: Dive 6 (RV06); **Record Level:** language: en; institutionCode: UHM; datasetName: ABYSSLINE; basisOfRecord: HumanObservation
- **Type status:** Other material. **Occurrence:** catalogNumber: AB02-EB02-CS-18; recordNumber: AB02-EB02-CS-18; NHM1372; recordedBy: Diva J Amon, Amanda F Ziegler; individualCount: 1; lifeStage: Adult; behavior: On seafloor; occurrenceStatus: present; preparations: tissue and DNA voucher stored in 80% non-denatured ethanol aqueous solution and remainder of animal preserved in 4% formaldehyde; associatedReferences: 10.1038/srep30492Amon DJ, Ziegler AF, Dahlgren TG, Glover AG, Goineau A, Gooday AJ, Wiklund H, Smith CR. Insights into the abundance and diversity of abyssal megafauna in a polymetallic-nodule region in the eastern Clarion-Clipperton Zone. Scientific Reports. 2016;6. doi: 10.1038/srep30492; **Taxon:** taxonConceptID: Aceste ovataAceste ovata; scientificName: Aceste ovataAceste ovata; kingdom: AnimaliaAnimalia; phylum: EchinodermataEchinodermata; class: EchinoideaEchinoidea; order: SpatangoidaSpatangoida; family: SchizasteridaeSchizasteridae; genus: AcesteAceste; taxonRank: species; scientificNameAuthorship: A Agassiz & HL Clark, 1907; **Location:** waterBody: Pacific Ocean; stateProvince: Clarion-Clipperton Zone; locality: UK Seabed Resources Ltd exploration contract area (UK-1); verbatimLocality: UK-1 Stratum B; maximumDepthInMeters: 4647; locationRemarks: RV Thompson Cruise TN319; decimalLatitude: 12.531; decimalLongitude: -116.6233; geodeticDatum: WGS85; coordinateUncertaintyInMeters: 500; **Identification:** identifiedBy: Richard Mooi, Diva J Amon, Amanda F Ziegler; dateIdentified: 2015; identificationRemarks: Identified by morphology and DNA of collected specimen; **Event:** samplingProtocol: Brenke Epibenthic Sled; eventDate: 2015-02-20; eventTime: 22:09; habitat: Abyssal polymetallic-nodule field; fieldNumber: Brenke Epibenthic Sled (EB02); **Record Level:** language: en; institutionCode: UHM; datasetName: ABYSSLINE; basisOfRecord: PreservedSpecimen

##### Notes

Fig. 28

#### Holothuroidea

#### Aspidochirotida

Grube, 1840

#### Mesothuriidae

Smirnov, 2012

#### Mesothuria

Ludwig, 1894

#### cf. Mesothuria morphospecies

cf. Mesothuria morphospecies In the “Atlas of Abyssal Megafauna Morphotypes of the Clarion-Clipperton Fracture Zone” created for the ISA (http://ccfzatlas.com/), this morphospecies is listed as "*Mesothuria* morphotype".

##### Materials

- **Type status:** Other material. **Occurrence:** recordedBy: Diva J Amon, Amanda F Ziegler; individualCount: 1; lifeStage: Adult; behavior: On seafloor; occurrenceStatus: present; preparations: Imaged only; associatedReferences: 10.1038/srep30492Amon DJ, Ziegler AF, Dahlgren TG, Glover AG, Goineau A, Gooday AJ, Wiklund H, Smith CR. Insights into the abundance and diversity of abyssal megafauna in a polymetallic-nodule region in the eastern Clarion-Clipperton Zone. Scientific Reports. 2016;6. doi: 10.1038/srep30492; **Taxon:** taxonConceptID: *Mesothuria*cf. *Mesothuria* morphospecies; scientificName: *MesothuriaMesothuria* sp.; kingdom: AnimaliaAnimalia; phylum: EchinodermataEchinodermata; class: HolothuroideaHolothuroidea; order: AspidochirotidaAspidochirotida; family: MesothuriidaeMesothuriidae; genus: MesothuriaMesothuria; taxonRank: genus; scientificNameAuthorship: Ludwig, 1894; **Location:** waterBody: Pacific Ocean; stateProvince: Clarion-Clipperton Zone; locality: Eastern Clarion-Clipperton Zone; verbatimLocality: Site EPIRB; maximumDepthInMeters: 3918; locationRemarks: RV Melville Cruise MV1313; decimalLatitude: 13.6793; decimalLongitude: -114.4074; geodeticDatum: WGS84; coordinateUncertaintyInMeters: 25; **Identification:** identifiedBy: Antonina Kremenetskaia, David L Pawson, Diva J Amon, Amanda F Ziegler; dateIdentified: 2014; identificationRemarks: Identified only from imagery; identificationQualifier: cf.; **Event:** samplingProtocol: Remotely Operated Vehicle; eventDate: 2013-10-23; eventTime: 11:55; habitat: Abyssal polymetallic-nodule field; fieldNumber: Dive 7 (RV07); **Record Level:** language: en; institutionCode: UHM; datasetName: ABYSSLINE; basisOfRecord: HumanObservation
- **Type status:** Other material. **Occurrence:** recordedBy: Diva J Amon, Amanda F Ziegler; individualCount: 1; lifeStage: Adult; behavior: On seafloor; occurrenceStatus: present; preparations: Imaged only; **Taxon:** taxonConceptID: *Mesothuria*cf. *Mesothuria* morphospecies; scientificName: *MesothuriaMesothuria* sp.; kingdom: AnimaliaAnimalia; phylum: EchinodermataEchinodermata; class: HolothuroideaHolothuroidea; order: AspidochirotidaAspidochirotida; family: MesothuriidaeMesothuriidae; genus: MesothuriaMesothuria; taxonRank: genus; scientificNameAuthorship: Ludwig, 1894; **Location:** waterBody: Pacific Ocean; stateProvince: Clarion-Clipperton Zone; locality: UK Seabed Resources Ltd exploration contract area (UK-1); verbatimLocality: UK-1 Stratum B; maximumDepthInMeters: 4221; locationRemarks: RV Thompson Cruise TN319; decimalLatitude: 12.5767; decimalLongitude: -116.6767; geodeticDatum: WGS84; coordinateUncertaintyInMeters: 25; **Identification:** identifiedBy: Antonina Kremenetskaia, David L Pawson, Diva J Amon, Amanda F Ziegler; dateIdentified: 2015; identificationRemarks: Identified only from imagery; identificationQualifier: cf.; **Event:** samplingProtocol: Autonomous Underwater Vehicle; eventDate: 2015-03-09; eventTime: 4:12; habitat: Abyssal polymetallic-nodule field; fieldNumber: Dive 6 (AV06); **Record Level:** language: en; institutionCode: UHM; datasetName: ABYSSLINE; basisOfRecord: HumanObservation

##### Notes

Fig. 29

#### Synallactidae

Ludwig, 1894

#### cf. Synallactidae morphospecies 1

##### Materials

- **Type status:** Other material. **Occurrence:** recordedBy: Diva J Amon, Amanda F Ziegler; individualCount: 1; lifeStage: Adult; behavior: On seafloor; occurrenceStatus: present; preparations: Imaged only; associatedReferences: 10.1038/srep30492Amon DJ, Ziegler AF, Dahlgren TG, Glover AG, Goineau A, Gooday AJ, Wiklund H, Smith CR. Insights into the abundance and diversity of abyssal megafauna in a polymetallic-nodule region in the eastern Clarion-Clipperton Zone. Scientific Reports. 2016;6. doi: 10.1038/srep30492; **Taxon:** taxonConceptID: Synallactidaecf. Synallactidae morphospecies 1; scientificName: SynallactidaeSynallactidae sp.; kingdom: AnimaliaAnimalia; phylum: EchinodermataEchinodermata; class: HolothuroideaHolothuroidea; order: AspidochirotidaAspidochirotida; family: SynallactidaeSynallactidae; taxonRank: family; scientificNameAuthorship: Ludwig, 1894; **Location:** waterBody: Pacific Ocean; stateProvince: Clarion-Clipperton Zone; locality: Eastern Clarion-Clipperton Zone; verbatimLocality: Site EPIRB; maximumDepthInMeters: 3922; locationRemarks: RV Melville Cruise MV1313; decimalLatitude: 13.6793; decimalLongitude: -114.4096; geodeticDatum: WGS84; coordinateUncertaintyInMeters: 25; **Identification:** identifiedBy: Antonina Kremenetskaia, David L Pawson, Diva J Amon, Amanda F Ziegler; dateIdentified: 2014; identificationRemarks: Identified only from imagery; identificationQualifier: cf.; **Event:** samplingProtocol: Remotely Operated Vehicle; eventDate: 2013-10-23; eventTime: 12:17; habitat: Abyssal polymetallic-nodule field; fieldNumber: Dive 7 (RV07); **Record Level:** language: en; institutionCode: UHM; datasetName: ABYSSLINE; basisOfRecord: HumanObservation
- **Type status:** Other material. **Occurrence:** recordedBy: Diva J Amon, Amanda F Ziegler; individualCount: 1; lifeStage: Adult; behavior: On seafloor; occurrenceStatus: present; preparations: Imaged only; **Taxon:** taxonConceptID: Synallactidaecf. Synallactidae morphospecies 1; scientificName: SynallactidaeSynallactidae sp.; kingdom: AnimaliaAnimalia; phylum: EchinodermataEchinodermata; class: HolothuroideaHolothuroidea; order: AspidochirotidaAspidochirotida; family: SynallactidaeSynallactidae; taxonRank: family; scientificNameAuthorship: Ludwig, 1894; **Location:** waterBody: Pacific Ocean; stateProvince: Clarion-Clipperton Zone; locality: UK Seabed Resources Ltd exploration contract area (UK-1); verbatimLocality: UK-1 Stratum B; maximumDepthInMeters: 4219; locationRemarks: RV Thompson Cruise TN319; decimalLatitude: 12.57901; decimalLongitude: -116.6978; geodeticDatum: WGS84; coordinateUncertaintyInMeters: 25; **Identification:** identifiedBy: Antonina Kremenetskaia, David L Pawson, Diva J Amon, Amanda F Ziegler; dateIdentified: 2015; identificationRemarks: Identified only from imagery; identificationQualifier: cf.; **Event:** samplingProtocol: Autonomous Underwater Vehicle; eventDate: 2015-03-09; eventTime: 15:36; habitat: Abyssal polymetallic-nodule field; fieldNumber: Dive 6 (AV06); **Record Level:** language: en; institutionCode: UHM; datasetName: ABYSSLINE; basisOfRecord: HumanObservation
- **Type status:** Other material. **Occurrence:** recordedBy: Diva J Amon, Amanda F Ziegler; individualCount: 1; lifeStage: Adult; behavior: On seafloor; occurrenceStatus: present; preparations: Imaged only; **Taxon:** taxonConceptID: Synallactidaecf. Synallactidae morphospecies 1; scientificName: SynallactidaeSynallactidae sp.; kingdom: AnimaliaAnimalia; phylum: EchinodermataEchinodermata; class: HolothuroideaHolothuroidea; order: AspidochirotidaAspidochirotida; family: SynallactidaeSynallactidae; taxonRank: family; scientificNameAuthorship: Ludwig, 1894; **Location:** waterBody: Pacific Ocean; stateProvince: Clarion-Clipperton Zone; locality: UK Seabed Resources Ltd exploration contract area (UK-1); verbatimLocality: UK-1 Stratum B; maximumDepthInMeters: 4225; locationRemarks: RV Thompson Cruise TN319; decimalLatitude: 12.5797; decimalLongitude: -116.7276; geodeticDatum: WGS84; coordinateUncertaintyInMeters: 25; **Identification:** identifiedBy: Antonina Kremenetskaia, David L Pawson, Diva J Amon, Amanda F Ziegler; dateIdentified: 2015; identificationRemarks: Identified only from imagery; identificationQualifier: cf.; **Event:** samplingProtocol: Autonomous Underwater Vehicle; eventDate: 2015-03-09; eventTime: 7:55; habitat: Abyssal polymetallic-nodule field; fieldNumber: Dive 6 (AV06); **Record Level:** language: en; institutionCode: UHM; datasetName: ABYSSLINE; basisOfRecord: HumanObservation

##### Notes

Fig. 30

#### cf. Synallactidae morphospecies 2

##### Materials

- **Type status:** Other material. **Occurrence:** recordedBy: Diva J Amon, Amanda F Ziegler; individualCount: 1; lifeStage: Adult; behavior: On seafloor; occurrenceStatus: present; preparations: Imaged only; **Taxon:** taxonConceptID: Synallactidaecf. Synallactidae morphospecies 2; scientificName: SynallactidaeSynallactidae sp.; kingdom: AnimaliaAnimalia; phylum: EchinodermataEchinodermata; class: HolothuroideaHolothuroidea; order: AspidochirotidaAspidochirotida; family: SynallactidaeSynallactidae; taxonRank: family; scientificNameAuthorship: Ludwig, 1894; **Location:** waterBody: Pacific Ocean; stateProvince: Clarion-Clipperton Zone; locality: UK Seabed Resources Ltd exploration contract area (UK-1); verbatimLocality: UK-1 Stratum B; maximumDepthInMeters: 4216; locationRemarks: RV Thompson Cruise TN319; decimalLatitude: 12.5894; decimalLongitude: -116.7152; geodeticDatum: WGS84; coordinateUncertaintyInMeters: 25; **Identification:** identifiedBy: Antonina Kremenetskaia, David L Pawson, Diva J Amon, Amanda F Ziegler; dateIdentified: 2015; identificationRemarks: Identified only from imagery; identificationQualifier: cf.; **Event:** samplingProtocol: Autonomous Underwater Vehicle; eventDate: 2015-03-09; eventTime: 9:47; habitat: Abyssal polymetallic-nodule field; fieldNumber: Dive 6 (AV06); **Record Level:** language: en; institutionCode: UHM; datasetName: ABYSSLINE; basisOfRecord: HumanObservation

##### Notes

Fig. 31

#### Molpadiodemas

Heding, 1935

#### cf. Molpadiodemas morphospecies

cf. Molpadiodemas morphospecies In the “Atlas of Abyssal Megafauna Morphotypes of the Clarion-Clipperton Fracture Zone” created for the ISA (http://ccfzatlas.com/), this morphospecies is listed as "*Molpadiodemas*​ morphotype".

##### Materials

- **Type status:** Other material. **Occurrence:** recordedBy: Diva J Amon, Amanda F Ziegler; individualCount: 1; lifeStage: Adult; behavior: On seafloor; occurrenceStatus: present; preparations: Imaged only; associatedReferences: 10.1038/srep30492Amon DJ, Ziegler AF, Dahlgren TG, Glover AG, Goineau A, Gooday AJ, Wiklund H, Smith CR. Insights into the abundance and diversity of abyssal megafauna in a polymetallic-nodule region in the eastern Clarion-Clipperton Zone. Scientific Reports. 2016;6. doi: 10.1038/srep30492; **Taxon:** taxonConceptID: *Molpadiodemas*cf. *Molpadiodemas* morphospecies; scientificName: *MolpadiodemasMolpadiodemas* sp.; kingdom: AnimaliaAnimalia; phylum: EchinodermataEchinodermata; class: HolothuroideaHolothuroidea; order: AspidochirotidaAspidochirotida; family: SynallactidaeSynallactidae; genus: MolpadiodemasMolpadiodemas; taxonRank: genus; scientificNameAuthorship: Heding, 1935; **Location:** waterBody: Pacific Ocean; stateProvince: Clarion-Clipperton Zone; locality: UK Seabed Resources Ltd exploration contract area (UK-1); verbatimLocality: UK-1 Stratum A; maximumDepthInMeters: 4053; locationRemarks: RV Melville Cruise MV1313; decimalLatitude: 13.9608; decimalLongitude: -116.5548; geodeticDatum: WGS84; coordinateUncertaintyInMeters: 25; **Identification:** identifiedBy: Antonina Kremenetskaia, David L Pawson, Diva J Amon, Amanda F Ziegler; dateIdentified: 2014; identificationRemarks: Identified only from imagery; identificationQualifier: cf.; **Event:** samplingProtocol: Remotely Operated Vehicle; eventDate: 2013-10-16; eventTime: 3:43; habitat: Abyssal polymetallic-nodule field; fieldNumber: Dive 3 (RV03); **Record Level:** language: en; institutionCode: UHM; datasetName: ABYSSLINE; basisOfRecord: HumanObservation
- **Type status:** Other material. **Occurrence:** recordedBy: Diva J Amon, Amanda F Ziegler; individualCount: 1; lifeStage: Adult; behavior: On seafloor; occurrenceStatus: present; preparations: Imaged only; associatedReferences: 10.1038/srep30492Amon DJ, Ziegler AF, Dahlgren TG, Glover AG, Goineau A, Gooday AJ, Wiklund H, Smith CR. Insights into the abundance and diversity of abyssal megafauna in a polymetallic-nodule region in the eastern Clarion-Clipperton Zone. Scientific Reports. 2016;6. doi: 10.1038/srep30492; **Taxon:** taxonConceptID: *Molpadiodemas*cf. *Molpadiodemas* morphospecies; scientificName: *MolpadiodemasMolpadiodemas* sp.; kingdom: AnimaliaAnimalia; phylum: EchinodermataEchinodermata; class: HolothuroideaHolothuroidea; order: AspidochirotidaAspidochirotida; family: SynallactidaeSynallactidae; genus: MolpadiodemasMolpadiodemas; taxonRank: genus; scientificNameAuthorship: Heding, 1935; **Location:** waterBody: Pacific Ocean; stateProvince: Clarion-Clipperton Zone; locality: UK Seabed Resources Ltd exploration contract area (UK-1); verbatimLocality: UK-1 Stratum A; maximumDepthInMeters: 4055; locationRemarks: RV Melville Cruise MV1313; decimalLatitude: 13.9611; decimalLongitude: -116.5541; geodeticDatum: WGS84; coordinateUncertaintyInMeters: 25; **Identification:** identifiedBy: Antonina Kremenetskaia, David L Pawson, Diva J Amon, Amanda F Ziegler; dateIdentified: 2014; identificationRemarks: Identified only from imagery; identificationQualifier: cf.; **Event:** samplingProtocol: Remotely Operated Vehicle; eventDate: 2013-10-16; eventTime: 3:52; habitat: Abyssal polymetallic-nodule field; fieldNumber: Dive 3 (RV03); **Record Level:** language: en; institutionCode: UHM; datasetName: ABYSSLINE; basisOfRecord: HumanObservation
- **Type status:** Other material. **Occurrence:** recordedBy: Diva J Amon, Amanda F Ziegler; individualCount: 1; lifeStage: Adult; behavior: On seafloor; occurrenceStatus: present; preparations: Imaged only; **Taxon:** taxonConceptID: *Molpadiodemas*cf. *Molpadiodemas* morphospecies; scientificName: *MolpadiodemasMolpadiodemas* sp.; kingdom: AnimaliaAnimalia; phylum: EchinodermataEchinodermata; class: HolothuroideaHolothuroidea; order: AspidochirotidaAspidochirotida; family: SynallactidaeSynallactidae; genus: MolpadiodemasMolpadiodemas; taxonRank: genus; scientificNameAuthorship: Heding, 1935; **Location:** waterBody: Pacific Ocean; stateProvince: Clarion-Clipperton Zone; locality: UK Seabed Resources Ltd exploration contract area (UK-1); verbatimLocality: UK-1 Stratum B; maximumDepthInMeters: 4228; locationRemarks: RV Thompson Cruise TN319; decimalLatitude: 12.5801; decimalLongitude: -116.7228; geodeticDatum: WGS84; coordinateUncertaintyInMeters: 25; **Identification:** identifiedBy: Antonina Kremenetskaia, David L Pawson, Diva J Amon, Amanda F Ziegler; dateIdentified: 2015; identificationRemarks: Identified only from imagery; identificationQualifier: cf.; **Event:** samplingProtocol: Autonomous Underwater Vehicle; eventDate: 2015-03-09; eventTime: 11:04; habitat: Abyssal polymetallic-nodule field; fieldNumber: Dive 6 (AV06); **Record Level:** language: en; institutionCode: UHM; datasetName: ABYSSLINE; basisOfRecord: HumanObservation
- **Type status:** Other material. **Occurrence:** recordedBy: Diva J Amon, Amanda F Ziegler; individualCount: 1; lifeStage: Adult; behavior: On seafloor; occurrenceStatus: present; preparations: Imaged only; **Taxon:** taxonConceptID: *Molpadiodemas*cf. *Molpadiodemas* morphospecies; scientificName: *MolpadiodemasMolpadiodemas* sp.; kingdom: AnimaliaAnimalia; phylum: EchinodermataEchinodermata; class: HolothuroideaHolothuroidea; order: AspidochirotidaAspidochirotida; family: SynallactidaeSynallactidae; genus: MolpadiodemasMolpadiodemas; taxonRank: genus; scientificNameAuthorship: Heding, 1935; **Location:** waterBody: Pacific Ocean; stateProvince: Clarion-Clipperton Zone; locality: UK Seabed Resources Ltd exploration contract area (UK-1); verbatimLocality: UK-1 Stratum B; maximumDepthInMeters: 4213; locationRemarks: RV Thompson Cruise TN319; decimalLatitude: 12.5708; decimalLongitude: -116.7066; geodeticDatum: WGS84; coordinateUncertaintyInMeters: 25; **Identification:** identifiedBy: Antonina Kremenetskaia, David L Pawson, Diva J Amon, Amanda F Ziegler; dateIdentified: 2015; identificationRemarks: Identified only from imagery; identificationQualifier: cf.; **Event:** samplingProtocol: Autonomous Underwater Vehicle; eventDate: 2015-03-09; eventTime: 15:07; habitat: Abyssal polymetallic-nodule field; fieldNumber: Dive 6 (AV06); **Record Level:** language: en; institutionCode: UHM; datasetName: ABYSSLINE; basisOfRecord: HumanObservation

##### Notes

Fig. 32

#### Paroriza

Herouard, 1902

#### cf. Paroriza morphospecies

cf. Paroriza morphospecies In the “Atlas of Abyssal Megafauna Morphotypes of the Clarion-Clipperton Fracture Zone” created for the ISA (http://ccfzatlas.com/), this morphospecies is listed as "*Paroriza* morphotype".

##### Materials

- **Type status:** Other material. **Occurrence:** recordedBy: Diva J Amon, Amanda F Ziegler; individualCount: 2; lifeStage: Adult; behavior: On seafloor; occurrenceStatus: present; preparations: Imaged only; **Taxon:** taxonConceptID: *Paroriza*cf. *Paroriza* morphospecies; scientificName: *ParorizaParoriza* sp.; kingdom: AnimaliaAnimalia; phylum: EchinodermataEchinodermata; class: HolothuroideaHolothuroidea; order: AspidochirotidaAspidochirotida; family: SynallactidaeSynallactidae; genus: ParorizaParoriza; taxonRank: genus; scientificNameAuthorship: Herouard, 1902; **Location:** waterBody: Pacific Ocean; stateProvince: Clarion-Clipperton Zone; locality: UK Seabed Resources Ltd exploration contract area (UK-1); verbatimLocality: UK-1 Stratum B; maximumDepthInMeters: 4217; locationRemarks: RV Thompson Cruise TN319; decimalLatitude: 12.57799; decimalLongitude: -116.7028; geodeticDatum: WGS84; coordinateUncertaintyInMeters: 25; **Identification:** identifiedBy: Antonina Kremenetskaia, David L Pawson, Diva J Amon, Amanda F Ziegler; dateIdentified: 2015; identificationRemarks: Identified only from imagery; identificationQualifier: cf.; **Event:** samplingProtocol: Autonomous Underwater Vehicle; eventDate: 2015-03-09; eventTime: 15:24; habitat: Abyssal polymetallic-nodule field; fieldNumber: Dive 6 (AV06); **Record Level:** language: en; institutionCode: UHM; datasetName: ABYSSLINE; basisOfRecord: HumanObservation

##### Notes

Fig. 33

#### Pseudostichopus

Théel, 1886

#### cf. Pseudostichopus morphospecies

cf. Pseudostichopus morphospecies In the “Atlas of Abyssal Megafauna Morphotypes of the Clarion-Clipperton Fracture Zone” created for the ISA (http://ccfzatlas.com/), this morphospecies is listed as "*Pseudostichopus* morphotype".

##### Materials

- **Type status:** Other material. **Occurrence:** recordedBy: Diva J Amon, Amanda F Ziegler; individualCount: 1; lifeStage: Adult; behavior: On seafloor; occurrenceStatus: present; preparations: Imaged only; associatedReferences: 10.1038/srep30492Amon DJ, Ziegler AF, Dahlgren TG, Glover AG, Goineau A, Gooday AJ, Wiklund H, Smith CR. Insights into the abundance and diversity of abyssal megafauna in a polymetallic-nodule region in the eastern Clarion-Clipperton Zone. Scientific Reports. 2016;6. doi: 10.1038/srep30492; **Taxon:** taxonConceptID: *Pseudostichopus*cf. *Pseudostichopus* morphospecies; scientificName: *PseudostichopusPseudostichopus* sp.; kingdom: AnimaliaAnimalia; phylum: EchinodermataEchinodermata; class: HolothuroideaHolothuroidea; order: AspidochirotidaAspidochirotida; family: SynallactidaeSynallactidae; genus: PseudostichopusPseudostichopus; taxonRank: genus; scientificNameAuthorship: Théel, 1886; **Location:** waterBody: Pacific Ocean; stateProvince: Clarion-Clipperton Zone; locality: UK Seabed Resources Ltd exploration contract area (UK-1); verbatimLocality: UK-1 Stratum A; maximumDepthInMeters: 4032; locationRemarks: RV Melville Cruise MV1313; decimalLatitude: 13.8628; decimalLongitude: -116.5485; geodeticDatum: WGS84; coordinateUncertaintyInMeters: 25; **Identification:** identifiedBy: Antonina Kremenetskaia, David L Pawson, Diva J Amon, Amanda F Ziegler; dateIdentified: 2014; identificationRemarks: Identified only from imagery; identificationQualifier: cf.; **Event:** samplingProtocol: Remotely Operated Vehicle; eventDate: 2013-10-21; eventTime: 4:59; habitat: Abyssal polymetallic-nodule field; fieldNumber: Dive 6 (RV06); **Record Level:** language: en; institutionCode: UHM; datasetName: ABYSSLINE; basisOfRecord: HumanObservation
- **Type status:** Other material. **Occurrence:** recordedBy: Diva J Amon, Amanda F Ziegler; individualCount: 1; lifeStage: Adult; behavior: On seafloor; occurrenceStatus: present; preparations: Imaged only; associatedReferences: 10.1038/srep30492Amon DJ, Ziegler AF, Dahlgren TG, Glover AG, Goineau A, Gooday AJ, Wiklund H, Smith CR. Insights into the abundance and diversity of abyssal megafauna in a polymetallic-nodule region in the eastern Clarion-Clipperton Zone. Scientific Reports. 2016;6. doi: 10.1038/srep30492; **Taxon:** taxonConceptID: *Pseudostichopus*cf. *Pseudostichopus* morphospecies; scientificName: *PseudostichopusPseudostichopus* sp.; kingdom: AnimaliaAnimalia; phylum: EchinodermataEchinodermata; class: HolothuroideaHolothuroidea; order: AspidochirotidaAspidochirotida; family: SynallactidaeSynallactidae; genus: PseudostichopusPseudostichopus; taxonRank: genus; scientificNameAuthorship: Théel, 1886; **Location:** waterBody: Pacific Ocean; stateProvince: Clarion-Clipperton Zone; locality: UK Seabed Resources Ltd exploration contract area (UK-1); verbatimLocality: UK-1 Stratum A; maximumDepthInMeters: 4028; locationRemarks: RV Melville Cruise MV1313; decimalLatitude: 13.8616; decimalLongitude: -116.5483; geodeticDatum: WGS84; coordinateUncertaintyInMeters: 25; **Identification:** identifiedBy: Antonina Kremenetskaia, David L Pawson, Diva J Amon, Amanda F Ziegler; dateIdentified: 2014; identificationRemarks: Identified only from imagery; identificationQualifier: cf.; **Event:** samplingProtocol: Remotely Operated Vehicle; eventDate: 2013-10-21; eventTime: 4:43; habitat: Abyssal polymetallic-nodule field; fieldNumber: Dive 6 (RV06); **Record Level:** language: en; institutionCode: UHM; datasetName: ABYSSLINE; basisOfRecord: HumanObservation

##### Notes

Fig. 34

#### Synallactes

Ludwig, 1894

#### cf. Synallactes morphospecies 1

cf. Synallactes morphospecies 1 In the “Atlas of Abyssal Megafauna Morphotypes of the Clarion-Clipperton Fracture Zone” created for the ISA (http://ccfzatlas.com/), this morphospecies is listed as "*Synallactes* morphotype "white"".

##### Materials

- **Type status:** Other material. **Occurrence:** recordedBy: Diva J Amon, Amanda F Ziegler; individualCount: 1; lifeStage: Adult; behavior: On seafloor; occurrenceStatus: present; preparations: Imaged only; associatedReferences: 10.1038/srep30492Amon DJ, Ziegler AF, Dahlgren TG, Glover AG, Goineau A, Gooday AJ, Wiklund H, Smith CR. Insights into the abundance and diversity of abyssal megafauna in a polymetallic-nodule region in the eastern Clarion-Clipperton Zone. Scientific Reports. 2016;6. doi: 10.1038/srep30492; **Taxon:** taxonConceptID: *Synallactes*cf. *Synallactes* morphospecies 1; scientificName: *SynallactesSynallactes* sp.; kingdom: AnimaliaAnimalia; phylum: EchinodermataEchinodermata; class: HolothuroideaHolothuroidea; order: AspidochirotidaAspidochirotida; family: SynallactidaeSynallactidae; genus: SynallactesSynallactes; taxonRank: genus; scientificNameAuthorship: Ludwig, 1894; **Location:** waterBody: Pacific Ocean; stateProvince: Clarion-Clipperton Zone; locality: UK Seabed Resources Ltd exploration contract area (UK-1); verbatimLocality: UK-1 Stratum A; maximumDepthInMeters: 4021; locationRemarks: RV Melville Cruise MV1313; decimalLatitude: 13.8578; decimalLongitude: -116.5481; geodeticDatum: WGS84; coordinateUncertaintyInMeters: 25; **Identification:** identifiedBy: Antonina Kremenetskaia, David L Pawson, Diva J Amon, Amanda F Ziegler; dateIdentified: 2014; identificationRemarks: Identified only from imagery; identificationQualifier: cf.; **Event:** samplingProtocol: Remotely Operated Vehicle; eventDate: 2013-10-21; eventTime: 3:48; habitat: Abyssal polymetallic-nodule field; fieldNumber: Dive 6 (RV06); **Record Level:** language: en; institutionCode: UHM; datasetName: ABYSSLINE; basisOfRecord: HumanObservation
- **Type status:** Other material. **Occurrence:** recordedBy: Diva J Amon, Amanda F Ziegler; individualCount: 1; lifeStage: Adult; behavior: On seafloor; occurrenceStatus: present; preparations: Imaged only; associatedReferences: 10.1038/srep30492Amon DJ, Ziegler AF, Dahlgren TG, Glover AG, Goineau A, Gooday AJ, Wiklund H, Smith CR. Insights into the abundance and diversity of abyssal megafauna in a polymetallic-nodule region in the eastern Clarion-Clipperton Zone. Scientific Reports. 2016;6. doi: 10.1038/srep30492; **Taxon:** taxonConceptID: *Synallactes*cf. *Synallactes* morphospecies 1; scientificName: *SynallactesSynallactes* sp.; kingdom: AnimaliaAnimalia; phylum: EchinodermataEchinodermata; class: HolothuroideaHolothuroidea; order: AspidochirotidaAspidochirotida; family: SynallactidaeSynallactidae; genus: SynallactesSynallactes; taxonRank: genus; scientificNameAuthorship: Ludwig, 1894; **Location:** waterBody: Pacific Ocean; stateProvince: Clarion-Clipperton Zone; locality: UK Seabed Resources Ltd exploration contract area (UK-1); verbatimLocality: UK-1 Stratum A; maximumDepthInMeters: 4079; locationRemarks: RV Melville Cruise MV1313; decimalLatitude: 13.8615; decimalLongitude: -116.5483; geodeticDatum: WGS84; coordinateUncertaintyInMeters: 25; **Identification:** identifiedBy: Antonina Kremenetskaia, David L Pawson, Diva J Amon, Amanda F Ziegler; dateIdentified: 2014; identificationRemarks: Identified only from imagery; identificationQualifier: cf.; **Event:** samplingProtocol: Remotely Operated Vehicle; eventDate: 2013-10-21; eventTime: 4:40; habitat: Abyssal polymetallic-nodule field; fieldNumber: Dive 6 (RV06); **Record Level:** language: en; institutionCode: UHM; datasetName: ABYSSLINE; basisOfRecord: HumanObservation
- **Type status:** Other material. **Occurrence:** recordedBy: Diva J Amon, Amanda F Ziegler; individualCount: 1; lifeStage: Adult; behavior: On seafloor; occurrenceStatus: present; preparations: Imaged only; associatedReferences: 10.1038/srep30492Amon DJ, Ziegler AF, Dahlgren TG, Glover AG, Goineau A, Gooday AJ, Wiklund H, Smith CR. Insights into the abundance and diversity of abyssal megafauna in a polymetallic-nodule region in the eastern Clarion-Clipperton Zone. Scientific Reports. 2016;6. doi: 10.1038/srep30492; **Taxon:** taxonConceptID: *Synallactes*cf. *Synallactes* morphospecies 1; scientificName: *SynallactesSynallactes* sp.; kingdom: AnimaliaAnimalia; phylum: EchinodermataEchinodermata; class: HolothuroideaHolothuroidea; order: AspidochirotidaAspidochirotida; family: SynallactidaeSynallactidae; genus: SynallactesSynallactes; taxonRank: genus; scientificNameAuthorship: Ludwig, 1894; **Location:** waterBody: Pacific Ocean; stateProvince: Clarion-Clipperton Zone; locality: Eastern Clarion-Clipperton Zone; verbatimLocality: Site EPIRB; maximumDepthInMeters: 3949; locationRemarks: RV Melville Cruise MV1313; decimalLatitude: 13.6794; decimalLongitude: -114.4138; geodeticDatum: WGS84; coordinateUncertaintyInMeters: 25; **Identification:** identifiedBy: Antonina Kremenetskaia, David L Pawson, Diva J Amon, Amanda F Ziegler; dateIdentified: 2014; identificationRemarks: Identified only from imagery; identificationQualifier: cf.; **Event:** samplingProtocol: Remotely Operated Vehicle; eventDate: 2013-10-23; eventTime: 9:57; habitat: Abyssal polymetallic-nodule field; fieldNumber: Dive 7 (RV07); **Record Level:** language: en; institutionCode: UHM; datasetName: ABYSSLINE; basisOfRecord: HumanObservation

##### Notes

Fig. 35

#### cf. Synallactes morphospecies 2

cf. Synallactes morphospecies 2 In the “Atlas of Abyssal Megafauna Morphotypes of the Clarion-Clipperton Fracture Zone” created for the ISA (http://ccfzatlas.com/), this morphospecies is listed as "*Synallactes* morphotype "pink"".

##### Materials

- **Type status:** Other material. **Occurrence:** recordedBy: Diva J Amon, Amanda F Ziegler; individualCount: 1; lifeStage: Adult; behavior: On seafloor; occurrenceStatus: present; preparations: Imaged only; **Taxon:** taxonConceptID: *Synallactes*cf. *Synallactes* morphospecies 2; scientificName: *SynallactesSynallactes* sp.; kingdom: AnimaliaAnimalia; phylum: EchinodermataEchinodermata; class: HolothuroideaHolothuroidea; order: AspidochirotidaAspidochirotida; family: SynallactidaeSynallactidae; genus: SynallactesSynallactes; taxonRank: genus; scientificNameAuthorship: Ludwig, 1894; **Location:** waterBody: Pacific Ocean; stateProvince: Clarion-Clipperton Zone; locality: UK Seabed Resources Ltd exploration contract area (UK-1); verbatimLocality: UK-1 Stratum B; maximumDepthInMeters: 4254; locationRemarks: RV Thompson Cruise TN319; decimalLatitude: 12.4955; decimalLongitude: -116.6505; geodeticDatum: WGS84; coordinateUncertaintyInMeters: 25; **Identification:** identifiedBy: Antonina Kremenetskaia, David L Pawson, Diva J Amon, Amanda F Ziegler; dateIdentified: 2015; identificationRemarks: Identified only from imagery; identificationQualifier: cf.; **Event:** samplingProtocol: Autonomous Underwater Vehicle; eventDate: 2015-03-18; eventTime: 8:46; habitat: Abyssal polymetallic-nodule field; fieldNumber: Dive 9 (AV09); **Record Level:** language: en; institutionCode: UHM; datasetName: ABYSSLINE; basisOfRecord: HumanObservation
- **Type status:** Other material. **Occurrence:** recordedBy: Diva J Amon, Amanda F Ziegler; individualCount: 1; lifeStage: Adult; behavior: On seafloor; occurrenceStatus: present; preparations: Imaged only; **Taxon:** taxonConceptID: *Synallactes*cf. *Synallactes* morphospecies 2; scientificName: *SynallactesSynallactes* sp.; kingdom: AnimaliaAnimalia; phylum: EchinodermataEchinodermata; class: HolothuroideaHolothuroidea; order: AspidochirotidaAspidochirotida; family: SynallactidaeSynallactidae; genus: SynallactesSynallactes; taxonRank: genus; scientificNameAuthorship: Ludwig, 1894; **Location:** waterBody: Pacific Ocean; stateProvince: Clarion-Clipperton Zone; locality: UK Seabed Resources Ltd exploration contract area (UK-1); verbatimLocality: UK-1 Stratum B; maximumDepthInMeters: 4224; locationRemarks: RV Thompson Cruise TN319; decimalLatitude: 12.4947; decimalLongitude: -116.6308; geodeticDatum: WGS84; coordinateUncertaintyInMeters: 25; **Identification:** identifiedBy: Antonina Kremenetskaia, David L Pawson, Diva J Amon, Amanda F Ziegler; dateIdentified: 2015; identificationRemarks: Identified only from imagery; identificationQualifier: cf.; **Event:** samplingProtocol: Autonomous Underwater Vehicle; eventDate: 2015-03-18; eventTime: 9:48; habitat: Abyssal polymetallic-nodule field; fieldNumber: Dive 9 (AV09); **Record Level:** language: en; institutionCode: UHM; datasetName: ABYSSLINE; basisOfRecord: HumanObservation

##### Notes

Fig. 36

#### cf. Synallactes morphospecies 3

cf. Synallactes morphospecies 3 In the “Atlas of Abyssal Megafauna Morphotypes of the Clarion-Clipperton Fracture Zone” created for the ISA (http://ccfzatlas.com/), this morphospecies is listed as "Synallactidae morphotype".

##### Materials

- **Type status:** Other material. **Occurrence:** recordedBy: Diva J Amon, Amanda F Ziegler; individualCount: 1; lifeStage: Adult; behavior: On seafloor; occurrenceStatus: present; preparations: Imaged only; **Taxon:** taxonConceptID: *Synallactes*cf. *Synallactes* morphospecies 3; scientificName: *SynallactesSynallactes* sp.; kingdom: AnimaliaAnimalia; phylum: EchinodermataEchinodermata; class: HolothuroideaHolothuroidea; order: AspidochirotidaAspidochirotida; family: SynallactidaeSynallactidae; genus: SynallactesSynallactes; taxonRank: genus; scientificNameAuthorship: Ludwig, 1894; **Location:** waterBody: Pacific Ocean; stateProvince: Clarion-Clipperton Zone; locality: UK Seabed Resources Ltd exploration contract area (UK-1); verbatimLocality: UK-1 Stratum B; maximumDepthInMeters: 4249; locationRemarks: RV Thompson Cruise TN319; decimalLatitude: 12.4991; decimalLongitude: -116.6409; geodeticDatum: WGS84; coordinateUncertaintyInMeters: 25; **Identification:** identifiedBy: Antonina Kremenetskaia, David L Pawson, Diva J Amon, Amanda F Ziegler; dateIdentified: 2015; identificationRemarks: Identified only from imagery; identificationQualifier: cf.; **Event:** samplingProtocol: Autonomous Underwater Vehicle; eventDate: 2015-03-03; eventTime: 21:52; habitat: Abyssal polymetallic-nodule field; fieldNumber: Dive 5 (AV05); **Record Level:** language: en; institutionCode: UHM; datasetName: ABYSSLINE; basisOfRecord: HumanObservation
- **Type status:** Other material. **Occurrence:** recordedBy: Diva J Amon, Amanda F Ziegler; individualCount: 1; lifeStage: Adult; behavior: On seafloor; occurrenceStatus: present; preparations: Imaged only; **Taxon:** taxonConceptID: *Synallactes*cf. *Synallactes* morphospecies 3; scientificName: *SynallactesSynallactes* sp.; kingdom: AnimaliaAnimalia; phylum: EchinodermataEchinodermata; class: HolothuroideaHolothuroidea; order: AspidochirotidaAspidochirotida; family: SynallactidaeSynallactidae; genus: SynallactesSynallactes; taxonRank: genus; scientificNameAuthorship: Ludwig, 1894; **Location:** waterBody: Pacific Ocean; stateProvince: Clarion-Clipperton Zone; locality: UK Seabed Resources Ltd exploration contract area (UK-1); verbatimLocality: UK-1 Stratum B; maximumDepthInMeters: 4253; locationRemarks: RV Thompson Cruise TN319; decimalLatitude: 12.4936; decimalLongitude: -116.6506; geodeticDatum: WGS84; coordinateUncertaintyInMeters: 25; **Identification:** identifiedBy: Antonina Kremenetskaia, David L Pawson, Diva J Amon, Amanda F Ziegler; dateIdentified: 2015; identificationRemarks: Identified only from imagery; identificationQualifier: cf.; **Event:** samplingProtocol: Autonomous Underwater Vehicle; eventDate: 2015-03-03; eventTime: 23:47; habitat: Abyssal polymetallic-nodule field; fieldNumber: Dive 5 (RV05); **Record Level:** language: en; institutionCode: UHM; datasetName: ABYSSLINE; basisOfRecord: HumanObservation

##### Notes

Fig. 37

#### Elasipodida

Théel, 1882

#### Deimatidae

Théel, 1882

#### cf. Deimatidae morphospecies 1

cf. Deimatidae morphospecies 1 In the “Atlas of Abyssal Megafauna Morphotypes of the Clarion-Clipperton Fracture Zone” created for the ISA (http://ccfzatlas.com/), this morphospecies is listed as "Deimatidae gen. sp. morphotype".

##### Materials

- **Type status:** Other material. **Occurrence:** recordedBy: Diva J Amon, Amanda F Ziegler; individualCount: 1; lifeStage: Adult; behavior: On seafloor; occurrenceStatus: present; preparations: Imaged only; **Taxon:** taxonConceptID: Deimatidaecf. Deimatidae morphospecies 1; scientificName: DeimatidaeDeimatidae sp.; kingdom: AnimaliaAnimalia; phylum: EchinodermataEchinodermata; class: HolothuroideaHolothuroidea; order: ElasipodidaElasipodida; family: DeimatidaeDeimatidae; taxonRank: family; scientificNameAuthorship: Théel, 1882; **Location:** waterBody: Pacific Ocean; stateProvince: Clarion-Clipperton Zone; locality: UK Seabed Resources Ltd exploration contract area (UK-1); verbatimLocality: UK-1 Stratum B; maximumDepthInMeters: 4162; locationRemarks: RV Thompson Cruise TN319; decimalLatitude: 12.3703; decimalLongitude: -116.5194; geodeticDatum: WGS84; coordinateUncertaintyInMeters: 25; **Identification:** identifiedBy: Antonina Kremenetskaia, David L Pawson, Diva J Amon, Amanda F Ziegler; dateIdentified: 2015; identificationRemarks: Identified only from imagery; identificationQualifier: cf.; **Event:** samplingProtocol: Autonomous Underwater Vehicle; eventDate: 2015-02-18; eventTime: 17:39; habitat: Abyssal polymetallic-nodule field; fieldNumber: Dive 1 (AV01); **Record Level:** language: en; institutionCode: UHM; datasetName: ABYSSLINE; basisOfRecord: HumanObservation
- **Type status:** Other material. **Occurrence:** recordedBy: Diva J Amon, Amanda F Ziegler; individualCount: 1; lifeStage: Adult; behavior: On seafloor; occurrenceStatus: present; preparations: Imaged only; **Taxon:** taxonConceptID: Deimatidaecf. Deimatidae morphospecies; scientificName: DeimatidaeDeimatidae sp.; kingdom: AnimaliaAnimalia; phylum: EchinodermataEchinodermata; class: HolothuroideaHolothuroidea; order: ElasipodidaElasipodida; family: DeimatidaeDeimatidae; taxonRank: family; scientificNameAuthorship: Théel, 1882; **Location:** waterBody: Pacific Ocean; stateProvince: Clarion-Clipperton Zone; locality: UK Seabed Resources Ltd exploration contract area (UK-1); verbatimLocality: UK-1 Stratum B; maximumDepthInMeters: 4254; locationRemarks: RV Thompson Cruise TN319; decimalLatitude: 12.4939; decimalLongitude: -116.6504; geodeticDatum: WGS84; coordinateUncertaintyInMeters: 25; **Identification:** identifiedBy: Antonina Kremenetskaia, David L Pawson, Diva J Amon, Amanda F Ziegler; dateIdentified: 2015; identificationRemarks: Identified only from imagery; identificationQualifier: cf.; **Event:** samplingProtocol: Autonomous Underwater Vehicle; eventDate: 2015-03-03; eventTime: 23:47; habitat: Abyssal polymetallic-nodule field; fieldNumber: Dive 5 (AV05); **Record Level:** language: en; institutionCode: UHM; datasetName: ABYSSLINE; basisOfRecord: HumanObservation

##### Notes

Fig. 38

#### cf. Deimatidae morphospecies 2

cf. Deimatidae morphospecies 2 In the “Atlas of Abyssal Megafauna Morphotypes of the Clarion-Clipperton Fracture Zone” created for the ISA (http://ccfzatlas.com/), this morphospecies is listed as "*Orphnurgus* morphotype".

##### Materials

- **Type status:** Other material. **Occurrence:** recordedBy: Diva J Amon, Amanda F Ziegler; individualCount: 1; lifeStage: Adult; behavior: On seafloor; occurrenceStatus: present; preparations: Imaged only; **Taxon:** taxonConceptID: *Deimatidae*cf. *Deimatidae* morphospecies 2; scientificName: DeimatidaeDeimatidae sp.; kingdom: AnimaliaAnimalia; phylum: EchinodermataEchinodermata; class: HolothuroideaHolothuroidea; order: ElasipodidaElasipodida; family: DeimatidaeDeimatidae; taxonRank: family; scientificNameAuthorship: Théel, 1882; **Location:** waterBody: Pacific Ocean; stateProvince: Clarion-Clipperton Zone; locality: UK Seabed Resources Ltd exploration contract area (UK-1); verbatimLocality: UK-1 Stratum B; maximumDepthInMeters: 4256; locationRemarks: RV Thompson Cruise TN319; decimalLatitude: 12.49096; decimalLongitude: -116.6511; geodeticDatum: WGS84; coordinateUncertaintyInMeters: 25; **Identification:** identifiedBy: Antonina Kremenetskaia, David L Pawson, Diva J Amon, Amanda F Ziegler; dateIdentified: 2015; identificationRemarks: Identified only from imagery; identificationQualifier: cf.; **Event:** samplingProtocol: Autonomous Underwater Vehicle; eventDate: 2015-03-03; eventTime: 22:23; habitat: Abyssal polymetallic-nodule field; fieldNumber: Dive 5 (AV05); **Record Level:** language: en; institutionCode: UHM; datasetName: ABYSSLINE; basisOfRecord: HumanObservation

##### Notes

Fig. 39

#### Deima

Théel, 1879

#### Deima cf. validum

Théel, 1879

Deima cf. validum In the “Atlas of Abyssal Megafauna Morphotypes of the Clarion-Clipperton Fracture Zone” created for the ISA (http://ccfzatlas.com/), this morphospecies is listed as "*Deima* morphotype".

##### Materials

- **Type status:** Other material. **Occurrence:** recordedBy: Diva J Amon, Amanda F Ziegler; individualCount: 1; lifeStage: Adult; behavior: On seafloor; occurrenceStatus: present; preparations: Imaged only; **Taxon:** taxonConceptID: Deima cf. validumDeima cf. validum; scientificName: Deima validumDeima validum; kingdom: AnimaliaAnimalia; phylum: EchinodermataEchinodermata; class: HolothuroideaHolothuroidea; order: ElasipodidaElasipodida; family: DeimatidaeDeimatidae; genus: DeimaDeima; taxonRank: species; scientificNameAuthorship: Théel, 1879; **Location:** waterBody: Pacific Ocean; stateProvince: Clarion-Clipperton Zone; locality: UK Seabed Resources Ltd exploration contract area (UK-1); verbatimLocality: UK-1 Stratum B; maximumDepthInMeters: 4254; locationRemarks: RV Thompson Cruise TN319; decimalLatitude: 12.4977; decimalLongitude: -116.6525; geodeticDatum: WGS84; coordinateUncertaintyInMeters: 25; **Identification:** identifiedBy: Antonina Kremenetskaia, David L Pawson, Diva J Amon, Amanda F Ziegler; dateIdentified: 2015; identificationRemarks: Identified only from imagery; identificationQualifier: cf.; **Event:** samplingProtocol: Autonomous Underwater Vehicle; eventDate: 2015-03-04; eventTime: 4:01; habitat: Abyssal polymetallic-nodule field; fieldNumber: Dive 5 (AV05); **Record Level:** language: en; institutionCode: UHM; datasetName: ABYSSLINE; basisOfRecord: HumanObservation
- **Type status:** Other material. **Occurrence:** recordedBy: Diva J Amon, Amanda F Ziegler; individualCount: 1; lifeStage: Adult; behavior: On seafloor; occurrenceStatus: present; preparations: Imaged only; **Taxon:** taxonConceptID: Deima cf. validumDeima cf. validum; scientificName: Deima validumDeima validum; kingdom: AnimaliaAnimalia; phylum: EchinodermataEchinodermata; class: HolothuroideaHolothuroidea; order: ElasipodidaElasipodida; family: DeimatidaeDeimatidae; genus: DeimaDeima; taxonRank: species; scientificNameAuthorship: Théel, 1879; **Location:** waterBody: Pacific Ocean; stateProvince: Clarion-Clipperton Zone; locality: UK Seabed Resources Ltd exploration contract area (UK-1); verbatimLocality: UK-1 Stratum B; maximumDepthInMeters: 4226; locationRemarks: RV Thompson Cruise TN319; decimalLatitude: 12.5814; decimalLongitude: -116.7244; geodeticDatum: WGS84; coordinateUncertaintyInMeters: 25; **Identification:** identifiedBy: Antonina Kremenetskaia, David L Pawson, Diva J Amon, Amanda F Ziegler; dateIdentified: 2015; identificationRemarks: Identified only from imagery; identificationQualifier: cf.; **Event:** samplingProtocol: Autonomous Underwater Vehicle; eventDate: 2015-03-09; eventTime: 8:42; habitat: Abyssal polymetallic-nodule field; fieldNumber: Dive 6 (AV06); **Record Level:** language: en; institutionCode: UHM; datasetName: ABYSSLINE; basisOfRecord: HumanObservation

##### Notes

Fig. 40

#### Oneirophanta

Théel, 1879

#### cf. Oneirophanta morphospecies

cf. Oneirophanta morphospecies In the “Atlas of Abyssal Megafauna Morphotypes of the Clarion-Clipperton Fracture Zone” created for the ISA (http://ccfzatlas.com/), this morphospecies is listed as "*Oneirophanta setigera* Ludwig, 1893".

##### Materials

- **Type status:** Other material. **Occurrence:** recordedBy: Diva J Amon, Amanda F Ziegler; individualCount: 1; lifeStage: Adult; behavior: On seafloor; occurrenceStatus: present; preparations: Imaged only; associatedReferences: 10.1038/srep30492Amon DJ, Ziegler AF, Dahlgren TG, Glover AG, Goineau A, Gooday AJ, Wiklund H, Smith CR. Insights into the abundance and diversity of abyssal megafauna in a polymetallic-nodule region in the eastern Clarion-Clipperton Zone. Scientific Reports. 2016;6. doi: 10.1038/srep30492; **Taxon:** taxonConceptID: *Oneirophanta*cf. *Oneirophanta* morphospecies; scientificName: *OneirophantaOneirophanta* sp.; kingdom: AnimaliaAnimalia; phylum: EchinodermataEchinodermata; class: HolothuroideaHolothuroidea; order: ElasipodidaElasipodida; family: DeimatidaeDeimatidae; genus: OneirophantaOneirophanta; taxonRank: genus; scientificNameAuthorship: Théel, 1879; **Location:** waterBody: Pacific Ocean; stateProvince: Clarion-Clipperton Zone; locality: UK Seabed Resources Ltd exploration contract area (UK-1); verbatimLocality: UK-1 Stratum A; maximumDepthInMeters: 4033; locationRemarks: RV Melville Cruise MV1313; decimalLatitude: 13.8627; decimalLongitude: -116.5484; geodeticDatum: WGS84; coordinateUncertaintyInMeters: 25; **Identification:** identifiedBy: Antonina Kremenetskaia, David L Pawson, Diva J Amon, Amanda F Ziegler; dateIdentified: 2014; identificationRemarks: Identified only from imagery; identificationQualifier: cf.; **Event:** samplingProtocol: Remotely Operated Vehicle; eventDate: 2013-10-21; eventTime: 4:57; habitat: Abyssal polymetallic-nodule field; fieldNumber: Dive 6 (RV06); **Record Level:** language: en; institutionCode: UHM; datasetName: ABYSSLINE; basisOfRecord: HumanObservation

##### Notes

Fig. 41

#### Elpidiidae

Théel, 1882

#### cf. Elpidiidae morphospecies 1

##### Materials

- **Type status:** Other material. **Occurrence:** recordedBy: Diva J Amon, Amanda F Ziegler; individualCount: 1; lifeStage: Young specimen; behavior: On seafloor; occurrenceStatus: present; preparations: Imaged only; associatedReferences: 10.1038/srep30492Amon DJ, Ziegler AF, Dahlgren TG, Glover AG, Goineau A, Gooday AJ, Wiklund H, Smith CR. Insights into the abundance and diversity of abyssal megafauna in a polymetallic-nodule region in the eastern Clarion-Clipperton Zone. Scientific Reports. 2016;6. doi: 10.1038/srep30492; **Taxon:** taxonConceptID: Elpidiidaecf. Elpidiidae morphospecies 1; scientificName: ElpidiidaeElpidiidae sp.; kingdom: AnimaliaAnimalia; phylum: EchinodermataEchinodermata; class: HolothuroideaHolothuroidea; order: ElasipodidaElasipodida; family: ElpidiidaeElpidiidae; taxonRank: family; scientificNameAuthorship: Théel, 1882; **Location:** waterBody: Pacific Ocean; stateProvince: Clarion-Clipperton Zone; locality: UK Seabed Resources Ltd exploration contract area (UK-1); verbatimLocality: UK-1 Stratum A; maximumDepthInMeters: 4020; locationRemarks: RV Melville Cruise MV1313; decimalLatitude: 13.8554; decimalLongitude: -116.5477; geodeticDatum: WGS84; coordinateUncertaintyInMeters: 25; **Identification:** identifiedBy: Antonina Kremenetskaia, David L Pawson, Diva J Amon, Amanda F Ziegler; dateIdentified: 2014; identificationRemarks: Identified only from imagery; identificationQualifier: cf.; **Event:** samplingProtocol: Remotely Operated Vehicle; eventDate: 2013-10-21; eventTime: 3:05; habitat: Abyssal polymetallic-nodule field; fieldNumber: Dive 6 (RV06); **Record Level:** language: en; institutionCode: UHM; datasetName: ABYSSLINE; basisOfRecord: HumanObservation

##### Notes

Fig. 42

#### cf. Elpidiidae morphospecies 2

cf. Elpidiidae morphospecies 2 In the “Atlas of Abyssal Megafauna Morphotypes of the Clarion-Clipperton Fracture Zone” created for the ISA (http://ccfzatlas.com/), this morphospecies is listed as "Elpidiidae morphotype "double velum"".

##### Materials

- **Type status:** Other material. **Occurrence:** recordedBy: Diva J Amon, Amanda F Ziegler; individualCount: 1; lifeStage: Adult; behavior: On seafloor; occurrenceStatus: present; preparations: Imaged only; **Taxon:** taxonConceptID: Elpidiidaecf. Elpidiidae morphospecies 2; scientificName: ElpidiidaeElpidiidae sp.; kingdom: AnimaliaAnimalia; phylum: EchinodermataEchinodermata; class: HolothuroideaHolothuroidea; order: ElasipodidaElasipodida; family: ElpidiidaeElpidiidae; taxonRank: family; scientificNameAuthorship: Théel, 1882; **Location:** waterBody: Pacific Ocean; stateProvince: Clarion-Clipperton Zone; locality: UK Seabed Resources Ltd exploration contract area (UK-1); verbatimLocality: UK-1 Stratum B; maximumDepthInMeters: 4254; locationRemarks: RV Thompson Cruise TN319; decimalLatitude: 12.4988; decimalLongitude: -116.6496; geodeticDatum: WGS84; coordinateUncertaintyInMeters: 25; **Identification:** identifiedBy: Antonina Kremenetskaia, David L Pawson, Diva J Amon, Amanda F Ziegler; dateIdentified: 2015; identificationRemarks: Identified only from imagery; identificationQualifier: cf.; **Event:** samplingProtocol: Autonomous Underwater Vehicle; eventDate: 2015-03-18; eventTime: 8:40; habitat: Abyssal polymetallic-nodule field; fieldNumber: Dive 9 (AV09); **Record Level:** language: en; institutionCode: UHM; datasetName: ABYSSLINE; basisOfRecord: HumanObservation

##### Notes

Fig. 43

#### Amperima

Pawson, 1965

#### cf. Amperima morphospecies

cf. Amperima morphospecies In the “Atlas of Abyssal Megafauna Morphotypes of the Clarion-Clipperton Fracture Zone” created for the ISA (http://ccfzatlas.com/), this morphospecies is listed as "*Amperima* morphotype".

##### Materials

- **Type status:** Other material. **Occurrence:** recordedBy: Diva J Amon, Amanda F Ziegler; individualCount: 1; lifeStage: Adult; behavior: On seafloor; occurrenceStatus: present; preparations: Imaged only; associatedReferences: 10.1038/srep30492Amon DJ, Ziegler AF, Dahlgren TG, Glover AG, Goineau A, Gooday AJ, Wiklund H, Smith CR. Insights into the abundance and diversity of abyssal megafauna in a polymetallic-nodule region in the eastern Clarion-Clipperton Zone. Scientific Reports. 2016;6. doi: 10.1038/srep30492; **Taxon:** taxonConceptID: *Amperima*cf. *Amperima* morphospecies; scientificName: *AmperimaAmperima* sp.; kingdom: AnimaliaAnimalia; phylum: EchinodermataEchinodermata; class: HolothuroideaHolothuroidea; order: ElasipodidaElasipodida; family: ElpidiidaeElpidiidae; genus: AmperimaAmperima; taxonRank: genus; scientificNameAuthorship: Pawson, 1965; **Location:** waterBody: Pacific Ocean; stateProvince: Clarion-Clipperton Zone; locality: Eastern Clarion-Clipperton Zone; verbatimLocality: Site EPIRB; maximumDepthInMeters: 3938; locationRemarks: RV Melville Cruise MV1313; decimalLatitude: 13.6794; decimalLongitude: -114.4113; geodeticDatum: WGS84; coordinateUncertaintyInMeters: 25; **Identification:** identifiedBy: Antonina Kremenetskaia, David L Pawson, Diva J Amon, Amanda F Ziegler; dateIdentified: 2014; identificationRemarks: Identified only from imagery; identificationQualifier: cf.; **Event:** samplingProtocol: Remotely Operated Vehicle; eventDate: 2013-10-23; eventTime: 12:36; habitat: Abyssal polymetallic-nodule field; fieldNumber: Dive 7 (RV07); **Record Level:** language: en; institutionCode: UHM; datasetName: ABYSSLINE; basisOfRecord: HumanObservation

##### Notes

Fig. 44

#### Peniagone

Théel, 1882

#### cf. Peniagone morphospecies 1

cf. Peniagone morphospecies 1 In the “Atlas of Abyssal Megafauna Morphotypes of the Clarion-Clipperton Fracture Zone” created for the ISA (http://ccfzatlas.com/), this morphospecies is listed as "*Peniagone* morphotype “pink, large velum"".

##### Materials

- **Type status:** Other material. **Occurrence:** recordedBy: Diva J Amon, Amanda F Ziegler; individualCount: 1; lifeStage: Adult; behavior: On seafloor; occurrenceStatus: present; preparations: Imaged only; **Taxon:** taxonConceptID: *Peniagone*cf. *Peniagone* morphospecies 1; scientificName: *PeniagonePeniagone* sp.; kingdom: AnimaliaAnimalia; phylum: EchinodermataEchinodermata; class: HolothuroideaHolothuroidea; order: ElasipodidaElasipodida; family: ElpidiidaeElpidiidae; genus: PeniagonePeniagone; taxonRank: genus; scientificNameAuthorship: Théel, 1882; **Location:** waterBody: Pacific Ocean; stateProvince: Clarion-Clipperton Zone; locality: UK Seabed Resources Ltd exploration contract area (UK-1); verbatimLocality: UK-1 Stratum B; maximumDepthInMeters: 4211; locationRemarks: RV Thompson Cruise TN319; decimalLatitude: 12.5887; decimalLongitude: -116.712; geodeticDatum: WGS84; coordinateUncertaintyInMeters: 25; **Identification:** identifiedBy: Antonina Kremenetskaia, David L Pawson, Diva J Amon, Amanda F Ziegler; dateIdentified: 2015; identificationRemarks: Identified only from imagery; identificationQualifier: cf.; **Event:** samplingProtocol: Autonomous Underwater Vehicle; eventDate: 2015-03-09; eventTime: 12:53; habitat: Abyssal polymetallic-nodule field; fieldNumber: Dive 6 (AV06); **Record Level:** language: en; institutionCode: UHM; datasetName: ABYSSLINE; basisOfRecord: HumanObservation

##### Notes

Fig. 45

#### cf. Peniagone morphospecies 2

cf. Peniagone morphospecies 2 In the “Atlas of Abyssal Megafauna Morphotypes of the Clarion-Clipperton Fracture Zone” created for the ISA (http://ccfzatlas.com/), this morphospecies is listed as "*Peniagone* morphotype "tulip"".

##### Materials

- **Type status:** Other material. **Occurrence:** recordedBy: Diva J Amon, Amanda F Ziegler; individualCount: 1; lifeStage: Adult; behavior: On seafloor; occurrenceStatus: present; preparations: Imaged only; **Taxon:** taxonConceptID: *Peniagone*cf. *Peniagone* morphospecies 2; scientificName: *PeniagonePeniagone* sp.; kingdom: AnimaliaAnimalia; phylum: EchinodermataEchinodermata; class: HolothuroideaHolothuroidea; order: ElasipodidaElasipodida; family: ElpidiidaeElpidiidae; genus: PeniagonePeniagone; taxonRank: genus; scientificNameAuthorship: Théel, 1882; **Location:** waterBody: Pacific Ocean; stateProvince: Clarion-Clipperton Zone; locality: UK Seabed Resources Ltd exploration contract area (UK-1); verbatimLocality: UK-1 Stratum B; maximumDepthInMeters: 4150; locationRemarks: RV Thompson Cruise TN319; decimalLatitude: 12.3712; decimalLongitude: -116.5117; geodeticDatum: WGS84; coordinateUncertaintyInMeters: 25; **Identification:** identifiedBy: Antonina Kremenetskaia, David L Pawson, Diva J Amon, Amanda F Ziegler; dateIdentified: 2015; identificationRemarks: Identified only from imagery; identificationQualifier: cf.; **Event:** samplingProtocol: Autonomous Underwater Vehicle; eventDate: 2015-02-18; eventTime: 13:33; habitat: Abyssal polymetallic-nodule field; fieldNumber: Dive 1 (AV01); **Record Level:** language: en; institutionCode: UHM; datasetName: ABYSSLINE; basisOfRecord: HumanObservation
- **Type status:** Other material. **Occurrence:** recordedBy: Diva J Amon, Amanda F Ziegler; individualCount: 1; lifeStage: Adult; behavior: On seafloor; occurrenceStatus: present; preparations: Imaged only; **Taxon:** taxonConceptID: *Peniagone*cf. *Peniagone* morphospecies 2; scientificName: *PeniagonePeniagone* sp.; kingdom: AnimaliaAnimalia; phylum: EchinodermataEchinodermata; class: HolothuroideaHolothuroidea; order: ElasipodidaElasipodida; family: ElpidiidaeElpidiidae; genus: PeniagonePeniagone; taxonRank: genus; scientificNameAuthorship: Théel, 1882; **Location:** waterBody: Pacific Ocean; stateProvince: Clarion-Clipperton Zone; locality: UK Seabed Resources Ltd exploration contract area (UK-1); verbatimLocality: UK-1 Stratum B; maximumDepthInMeters: 4227; locationRemarks: RV Thompson Cruise TN319; decimalLatitude: 12.5844; decimalLongitude: -116.7164; geodeticDatum: WGS84; coordinateUncertaintyInMeters: 25; **Identification:** identifiedBy: Antonina Kremenetskaia, David L Pawson, Diva J Amon, Amanda F Ziegler; dateIdentified: 2015; identificationRemarks: Identified only from imagery; identificationQualifier: cf.; **Event:** samplingProtocol: Autonomous Underwater Vehicle; eventDate: 2015-03-09; eventTime: 12:46; habitat: Abyssal polymetallic-nodule field; fieldNumber: Dive 6 (AV06); **Record Level:** language: en; institutionCode: UHM; datasetName: ABYSSLINE; basisOfRecord: HumanObservation

##### Notes

Fig. 46

#### Peniagone cf. leander

Pawson & Foell, 1986

Peniagone cf. leander In the “Atlas of Abyssal Megafauna Morphotypes of the Clarion-Clipperton Fracture Zone” created for the ISA (http://ccfzatlas.com/), this morphospecies is listed as "*Peniagone leander* Pawson & Foell, 1986".

##### Materials

- **Type status:** Other material. **Occurrence:** recordedBy: Diva J Amon, Amanda F Ziegler; individualCount: 1; lifeStage: Adult; behavior: Swimming; occurrenceStatus: present; preparations: Imaged only; associatedReferences: 10.1038/srep30492;*Peniagone leander*EchinodermataHolothuroideaAmon DJ, Ziegler AF, Dahlgren TG, Glover AG, Goineau A, Gooday AJ, Wiklund H, Smith CR. Insights into the abundance and diversity of abyssal megafauna in a polymetallic-nodule region in the eastern Clarion-Clipperton Zone. Scientific Reports. 2016, 6. doi: 10.1038/srep30492; Pawson DL & Foell EJ. *Peniagone leander* new species, an abyssal benthopelagic sea cucumber (Echinodermata: Holothuroidea) from the eastern Pacific Ocean. Bulletin of Marine Science. 1986, 38(2), 293-299.; **Taxon:** taxonConceptID: Peniagone cf. leanderPeniagone cf. leander; scientificName: Peniagone leanderPeniagone leander; kingdom: AnimaliaAnimalia; phylum: EchinodermataEchinodermata; class: HolothuroideaHolothuroidea; order: ElasipodidaElasipodida; family: ElpidiidaeElpidiidae; genus: PeniagonePeniagone; taxonRank: species; scientificNameAuthorship: Pawson & Foell, 1986; **Location:** waterBody: Pacific Ocean; stateProvince: Clarion-Clipperton Zone; locality: UK Seabed Resources Ltd exploration contract area (UK-1); verbatimLocality: UK-1 Stratum A; maximumDepthInMeters: 4060; locationRemarks: RV Melville Cruise MV1313; decimalLatitude: 13.9625; decimalLongitude: -116.5524; geodeticDatum: WGS84; coordinateUncertaintyInMeters: 25; **Identification:** identifiedBy: Antonina Kremenetskaia, David L Pawson, Diva J Amon, Amanda F Ziegler; dateIdentified: 2014; identificationRemarks: Identified only from imagery; identificationQualifier: cf.; **Event:** samplingProtocol: Remotely Operated Vehicle; eventDate: 2013-10-16; eventTime: 6:33; habitat: Abyssal polymetallic-nodule field; fieldNumber: Dive 3 (RV03); **Record Level:** language: en; institutionCode: UHM; datasetName: ABYSSLINE; basisOfRecord: HumanObservation

##### Notes

Fig. 47

#### Laetmogonidae

Ekman, 1926

#### cf. Laetmogonidae morphospecies

cf. Laetmogonidae morphospecies In the “Atlas of Abyssal Megafauna Morphotypes of the Clarion-Clipperton Fracture Zone” created for the ISA (http://ccfzatlas.com/), this morphospecies is listed as "Laetmogonidae gen. sp.".

##### Materials

- **Type status:** Other material. **Occurrence:** recordedBy: Diva J Amon, Amanda F Ziegler; individualCount: 1; lifeStage: Adult; behavior: On seafloor; occurrenceStatus: present; preparations: Imaged only; associatedReferences: 10.1038/srep30492Amon DJ, Ziegler AF, Dahlgren TG, Glover AG, Goineau A, Gooday AJ, Wiklund H, Smith CR. Insights into the abundance and diversity of abyssal megafauna in a polymetallic-nodule region in the eastern Clarion-Clipperton Zone. Scientific Reports. 2016;6. doi: 10.1038/srep30492; **Taxon:** taxonConceptID: Laetmogonidaecf. Laetmogonidae morphospecies; scientificName: LaetmogonidaeLaetmogonidae sp.; kingdom: AnimaliaAnimalia; phylum: EchinodermataEchinodermata; class: HolothuroideaHolothuroidea; order: ElasipodidaElasipodida; family: LaetmogonidaeLaetmogonidae; taxonRank: family; scientificNameAuthorship: Ekman, 1926; **Location:** waterBody: Pacific Ocean; stateProvince: Clarion-Clipperton Zone; locality: UK Seabed Resources Ltd exploration contract area (UK-1); verbatimLocality: UK-1 Stratum A; maximumDepthInMeters: 4107; locationRemarks: RV Melville Cruise MV1313; decimalLatitude: 13.8498; decimalLongitude: -116.6456; geodeticDatum: WGS84; coordinateUncertaintyInMeters: 25; **Identification:** identifiedBy: Antonina Kremenetskaia, David L Pawson, Diva J Amon, Amanda F Ziegler; dateIdentified: 2014; identificationRemarks: Identified only from imagery; identificationQualifier: cf.; **Event:** samplingProtocol: Remotely Operated Vehicle; eventDate: 2013-10-10; eventTime: 12:21; habitat: Abyssal polymetallic-nodule field; fieldNumber: Dive 1 (RV01); **Record Level:** language: en; institutionCode: UHM; datasetName: ABYSSLINE; basisOfRecord: HumanObservation

##### Notes

Fig. 48

#### Psychronaetes

Pawson, 1983

#### Psychronaetes cf. hanseni

Pawson, 1983

Psychronaetes cf. hanseni In the “Atlas of Abyssal Megafauna Morphotypes of the Clarion-Clipperton Fracture Zone” created for the ISA (http://ccfzatlas.com/), this morphospecies is listed as "*Psychronaetes hanseni* Pawson, 1983".

##### Materials

- **Type status:** Other material. **Occurrence:** recordedBy: Diva J Amon, Amanda F Ziegler; individualCount: 1; lifeStage: Adult; behavior: On seafloor; occurrenceStatus: present; preparations: Imaged only; associatedReferences: 10.1038/srep30492;*Psychronaetes hanseni*Amon DJ, Ziegler AF, Dahlgren TG, Glover AG, Goineau A, Gooday AJ, Wiklund H, Smith CR. Insights into the abundance and diversity of abyssal megafauna in a polymetallic-nodule region in the eastern Clarion-Clipperton Zone. Scientific Reports. 2016;6. doi: 10.1038/srep30492; *Psychronaetes hanseni*, a new genus and species of Elasipodan sea cucumber from the eastern central Pacific. Proceedings of the Biological Society of Washington 96 (1): 154‑159.; **Taxon:** taxonConceptID: Psychronaetes cf. hanseniPsychronaetes cf. hanseni; scientificName: Psychronaetes hanseniPsychronaetes hanseni; kingdom: AnimaliaAnimalia; phylum: EchinodermataEchinodermata; class: HolothuroideaHolothuroidea; order: ElasipodidaElasipodida; family: LaetmogonidaeLaetmogonidae; genus: PsychronaetesPsychronaetes; taxonRank: species; scientificNameAuthorship: Pawson, 1983; **Location:** waterBody: Pacific Ocean; stateProvince: Clarion-Clipperton Zone; locality: UK Seabed Resources Ltd exploration contract area (UK-1); verbatimLocality: UK-1 Stratum A; maximumDepthInMeters: 4107; locationRemarks: RV Melville Cruise MV1313; decimalLatitude: 13.8502; decimalLongitude: -116.6454; geodeticDatum: WGS84; coordinateUncertaintyInMeters: 25; **Identification:** identifiedBy: Antonina Kremenetskaia, David L Pawson, Diva J Amon, Amanda F Ziegler; dateIdentified: 2014; identificationRemarks: Identified only from imagery; identificationQualifier: cf.; **Event:** samplingProtocol: Remotely Operated Vehicle; eventDate: 2013-10-10; eventTime: 12:12; habitat: Abyssal polymetallic-nodule field; fieldNumber: Dive 1 (RV01); **Record Level:** language: en; institutionCode: UHM; datasetName: ABYSSLINE; basisOfRecord: HumanObservation
- **Type status:** Other material. **Occurrence:** recordedBy: Diva J Amon, Amanda F Ziegler; individualCount: 1; lifeStage: Adult; behavior: On seafloor; occurrenceStatus: present; preparations: Imaged only; **Taxon:** taxonConceptID: Psychronaetes cf. hanseniPsychronaetes cf. hanseni; scientificName: Psychronaetes hanseniPsychronaetes hanseni; kingdom: AnimaliaAnimalia; phylum: EchinodermataEchinodermata; class: HolothuroideaHolothuroidea; order: ElasipodidaElasipodida; family: LaetmogonidaeLaetmogonidae; genus: PsychronaetesPsychronaetes; taxonRank: species; scientificNameAuthorship: Pawson, 1983; **Location:** waterBody: Pacific Ocean; stateProvince: Clarion-Clipperton Zone; locality: UK Seabed Resources Ltd exploration contract area (UK-1); verbatimLocality: UK-1 Stratum B; maximumDepthInMeters: 4250; locationRemarks: RV Thompson Cruise TN319; decimalLatitude: 12.4945; decimalLongitude: -116.6489; geodeticDatum: WGS84; coordinateUncertaintyInMeters: 25; **Identification:** identifiedBy: Antonina Kremenetskaia, David L Pawson, Diva J Amon, Amanda F Ziegler; dateIdentified: 2015; identificationRemarks: Identified only from imagery; identificationQualifier: cf.; **Event:** samplingProtocol: Autonomous Underwater Vehicle; eventDate: 2015-03-03; eventTime: 22:44; habitat: Abyssal polymetallic-nodule field; fieldNumber: Dive 5 (AV05); **Record Level:** language: en; institutionCode: UHM; datasetName: ABYSSLINE; basisOfRecord: HumanObservation

##### Notes

Fig. 49

#### Pelagothuriidae

Ludwig, 1893

#### Enypniastes

Théel, 1882

#### cf. Enypniastes morphospecies

cf. Enypniastes morphospecies In the “Atlas of Abyssal Megafauna Morphotypes of the Clarion-Clipperton Fracture Zone” created for the ISA (http://ccfzatlas.com/), this morphospecies is listed as "*Enypniastes eximia* Théel, 1882".

##### Materials

- **Type status:** Other material. **Occurrence:** recordedBy: Jeffrey Drazen, Astrid Leitner; individualCount: 1; lifeStage: Adult; behavior: On seafloor; occurrenceStatus: present; preparations: Imaged only; **Taxon:** taxonConceptID: *Enypniastes*cf. *Enypniastes* morphospecies; scientificName: *EnypniastesEnypniastes* sp.; kingdom: AnimaliaAnimalia; phylum: EchinodermataEchinodermata; class: HolothuroideaHolothuroidea; order: ElasipodidaElasipodida; family: PelagothuriidaePelagothuriidae; genus: EnypniastesEnypniastes; taxonRank: genus; scientificNameAuthorship: Théel, 1882; **Location:** waterBody: Pacific Ocean; stateProvince: Clarion-Clipperton Zone; locality: UK Seabed Resources Ltd exploration contract area (UK-1); verbatimLocality: UK-1 Stratum B; maximumDepthInMeters: 4200; locationRemarks: RV Thompson Cruise TN319; decimalLatitude: 12.5669; decimalLongitude: -116.7084; geodeticDatum: WGS84; coordinateUncertaintyInMeters: 50; **Identification:** identifiedBy: Jeffrey Drazen, Astrid Leitner, Antonina Kremenetskaia, David L Pawson, Diva J Amon, Amanda F Ziegler; dateIdentified: 2015; identificationRemarks: Identified only from imagery; identificationQualifier: cf.; **Event:** samplingProtocol: Baited Camera; eventDate: 2015-02-21; eventTime: 13:06; habitat: Abyssal polymetallic-nodule field; fieldNumber: CA02; **Record Level:** language: en; institutionCode: UHM; datasetName: ABYSSLINE; basisOfRecord: HumanObservation

##### Notes

Fig. 50

#### Psychropotidae

Théel, 1882

#### cf. Psychropotidae morphospecies

##### Materials

- **Type status:** Other material. **Occurrence:** recordedBy: Diva J Amon, Amanda F Ziegler; individualCount: 1; lifeStage: Young specimen; behavior: On seafloor; occurrenceStatus: present; preparations: Imaged only; associatedReferences: 10.1038/srep30492Amon DJ, Ziegler AF, Dahlgren TG, Glover AG, Goineau A, Gooday AJ, Wiklund H, Smith CR. Insights into the abundance and diversity of abyssal megafauna in a polymetallic-nodule region in the eastern Clarion-Clipperton Zone. Scientific Reports. 2016;6. doi: 10.1038/srep30492; **Taxon:** taxonConceptID: Psychropotidaecf. Psychropotidae morphospecies; scientificName: PsychropotidaePsychropotidae sp.; kingdom: AnimaliaAnimalia; phylum: EchinodermataEchinodermata; class: HolothuroideaHolothuroidea; order: ElasipodidaElasipodida; family: PsychropotidaePsychropotidae; taxonRank: family; scientificNameAuthorship: Théel, 1882; **Location:** waterBody: Pacific Ocean; stateProvince: Clarion-Clipperton Zone; locality: UK Seabed Resources Ltd exploration contract area (UK-1); verbatimLocality: UK-1 Stratum A; maximumDepthInMeters: 4055; locationRemarks: RV Melville Cruise MV1313; decimalLatitude: 13.9673; decimalLongitude: -116.55896; geodeticDatum: WGS84; coordinateUncertaintyInMeters: 25; **Identification:** identifiedBy: Antonina Kremenetskaia, David L Pawson, Diva J Amon, Amanda F Ziegler; dateIdentified: 2014; identificationRemarks: Identified only from imagery; identificationQualifier: cf.; **Event:** samplingProtocol: Remotely Operated Vehicle; eventDate: 2013-10-16; eventTime: 0:18; habitat: Abyssal polymetallic-nodule field; fieldNumber: Dive 3 (RV03); **Record Level:** language: en; institutionCode: UHM; datasetName: ABYSSLINE; basisOfRecord: HumanObservation
- **Type status:** Other material. **Occurrence:** recordedBy: Diva J Amon, Amanda F Ziegler; individualCount: 1; lifeStage: Young specimen; behavior: On seafloor; occurrenceStatus: present; preparations: Imaged only; associatedReferences: 10.1038/srep30492Amon DJ, Ziegler AF, Dahlgren TG, Glover AG, Goineau A, Gooday AJ, Wiklund H, Smith CR. Insights into the abundance and diversity of abyssal megafauna in a polymetallic-nodule region in the eastern Clarion-Clipperton Zone. Scientific Reports. 2016;6. doi: 10.1038/srep30492; **Taxon:** taxonConceptID: Psychropotidaecf. Psychropotidae morphospecies; scientificName: PsychropotidaePsychropotidae sp.; kingdom: AnimaliaAnimalia; phylum: EchinodermataEchinodermata; class: HolothuroideaHolothuroidea; order: ElasipodidaElasipodida; family: PsychropotidaePsychropotidae; taxonRank: family; scientificNameAuthorship: Théel, 1882; **Location:** waterBody: Pacific Ocean; stateProvince: Clarion-Clipperton Zone; locality: Eastern Clarion-Clipperton Zone; verbatimLocality: Site EPIRB; maximumDepthInMeters: 3954; locationRemarks: RV Melville Cruise MV1313; decimalLatitude: 13.6797; decimalLongitude: -114.4145; geodeticDatum: WGS84; coordinateUncertaintyInMeters: 25; **Identification:** identifiedBy: Antonina Kremenetskaia, David L Pawson, Diva J Amon, Amanda F Ziegler; dateIdentified: 2014; identificationRemarks: Identified only from imagery; identificationQualifier: cf.; **Event:** samplingProtocol: Remotely Operated Vehicle; eventDate: 2013-10-23; eventTime: 13:27; habitat: Abyssal polymetallic-nodule field; fieldNumber: Dive 7 (RV07); **Record Level:** language: en; institutionCode: UHM; datasetName: ABYSSLINE; basisOfRecord: HumanObservation

##### Notes

Fig. 51

#### Benthodytes

Théel, 1882

#### cf. Benthodytes morphospecies 1

##### Materials

- **Type status:** Other material. **Occurrence:** recordedBy: Diva J Amon, Amanda F Ziegler; individualCount: 1; lifeStage: Adult; behavior: On seafloor; occurrenceStatus: present; preparations: Imaged only; **Taxon:** taxonConceptID: *Benthodytes*cf. *Benthodytes* morphospecies 1; scientificName: *BenthodytesBenthodytes* sp.; kingdom: AnimaliaAnimalia; phylum: EchinodermataEchinodermata; class: HolothuroideaHolothuroidea; order: ElasipodidaElasipodida; family: PsychropotidaePsychropotidae; genus: BenthodytesBenthodytes; taxonRank: genus; scientificNameAuthorship: Théel, 1882; **Location:** waterBody: Pacific Ocean; stateProvince: Clarion-Clipperton Zone; locality: UK Seabed Resources Ltd exploration contract area (UK-1); verbatimLocality: UK-1 Stratum B; maximumDepthInMeters: 4164; locationRemarks: RV Thompson Cruise TN319; decimalLatitude: 12.3688; decimalLongitude: -116.5207; geodeticDatum: WGS84; coordinateUncertaintyInMeters: 25; **Identification:** identifiedBy: Antonina Kremenetskaia, David L Pawson, Diva J Amon, Amanda F Ziegler; dateIdentified: 2015; identificationRemarks: Identified only from imagery; identificationQualifier: cf.; **Event:** samplingProtocol: Autonomous Underwater Vehicle; eventDate: 2015-02-18; eventTime: 15:37; habitat: Abyssal polymetallic-nodule field; fieldNumber: Dive 1 (AV01); **Record Level:** language: en; institutionCode: UHM; datasetName: ABYSSLINE; basisOfRecord: HumanObservation
- **Type status:** Other material. **Occurrence:** recordedBy: Diva J Amon, Amanda F Ziegler; individualCount: 1; lifeStage: Adult; behavior: On seafloor; occurrenceStatus: present; preparations: Imaged only; **Taxon:** taxonConceptID: *Benthodytes*cf. *Benthodytes* morphospecies 1; scientificName: *BenthodytesBenthodytes* sp.; kingdom: AnimaliaAnimalia; phylum: EchinodermataEchinodermata; class: HolothuroideaHolothuroidea; order: ElasipodidaElasipodida; family: PsychropotidaePsychropotidae; genus: BenthodytesBenthodytes; taxonRank: genus; scientificNameAuthorship: Théel, 1882; **Location:** waterBody: Pacific Ocean; stateProvince: Clarion-Clipperton Zone; locality: UK Seabed Resources Ltd exploration contract area (UK-1); verbatimLocality: UK-1 Stratum B; maximumDepthInMeters: 4255; locationRemarks: RV Thompson Cruise TN319; decimalLatitude: 12.5062; decimalLongitude: -116.6493; geodeticDatum: WGS84; coordinateUncertaintyInMeters: 25; **Identification:** identifiedBy: Antonina Kremenetskaia, David L Pawson, Diva J Amon, Amanda F Ziegler; dateIdentified: 2015; identificationRemarks: Identified only from imagery; identificationQualifier: cf.; **Event:** samplingProtocol: Autonomous Underwater Vehicle; eventDate: 2015-03-03; eventTime: 20:36; habitat: Abyssal polymetallic-nodule field; fieldNumber: Dive 5 (AV05); **Record Level:** language: en; institutionCode: UHM; datasetName: ABYSSLINE; basisOfRecord: HumanObservation

##### Notes

Fig. 52

#### cf. Benthodytes morphospecies 2

##### Materials

- **Type status:** Other material. **Occurrence:** recordedBy: Diva J Amon, Amanda F Ziegler; individualCount: 1; lifeStage: Young specimen; behavior: On seafloor; occurrenceStatus: present; preparations: Imaged only; associatedReferences: 10.1038/srep30492Amon DJ, Ziegler AF, Dahlgren TG, Glover AG, Goineau A, Gooday AJ, Wiklund H, Smith CR. Insights into the abundance and diversity of abyssal megafauna in a polymetallic-nodule region in the eastern Clarion-Clipperton Zone. Scientific Reports. 2016;6. doi: 10.1038/srep30492; **Taxon:** taxonConceptID: *Benthodytes*cf. *Benthodytes* morphospecies 2; scientificName: *BenthodytesBenthodytes* sp.; kingdom: AnimaliaAnimalia; phylum: EchinodermataEchinodermata; class: HolothuroideaHolothuroidea; order: ElasipodidaElasipodida; family: PsychropotidaePsychropotidae; genus: BenthodytesBenthodytes; taxonRank: genus; scientificNameAuthorship: Théel, 1882; **Location:** waterBody: Pacific Ocean; stateProvince: Clarion-Clipperton Zone; locality: UK Seabed Resources Ltd exploration contract area (UK-1); verbatimLocality: UK-1 Stratum A; maximumDepthInMeters: 4024; locationRemarks: RV Melville Cruise MV1313; decimalLatitude: 13.8585; decimalLongitude: -116.5472; geodeticDatum: WGS84; coordinateUncertaintyInMeters: 25; **Identification:** identifiedBy: Antonina Kremenetskaia, David L Pawson, Diva J Amon, Amanda F Ziegler; dateIdentified: 2015; identificationRemarks: Identified only from imagery; identificationQualifier: cf.; **Event:** samplingProtocol: Remotely Operated Vehicle; eventDate: 2013-10-21; eventTime: 2:28; habitat: Abyssal polymetallic-nodule field; fieldNumber: Dive 6 (RV06); **Record Level:** language: en; institutionCode: UHM; datasetName: ABYSSLINE; basisOfRecord: HumanObservation

##### Notes

Fig. 53

#### Benthodytes cf. incerta

Ludwig, 1893

Benthodytes cf. incerta In the “Atlas of Abyssal Megafauna Morphotypes of the Clarion-Clipperton Fracture Zone” created for the ISA (http://ccfzatlas.com/), this morphospecies is listed as "Benthodytes cf. incerta".

##### Materials

- **Type status:** Other material. **Occurrence:** recordedBy: Diva J Amon, Amanda F Ziegler; individualCount: 1; lifeStage: Adult; behavior: On rock; occurrenceStatus: present; preparations: Imaged only; associatedReferences: 10.1038/srep30492Amon DJ, Ziegler AF, Dahlgren TG, Glover AG, Goineau A, Gooday AJ, Wiklund H, Smith CR. Insights into the abundance and diversity of abyssal megafauna in a polymetallic-nodule region in the eastern Clarion-Clipperton Zone. Scientific Reports. 2016;6. doi: 10.1038/srep30492; **Taxon:** taxonConceptID: Benthodytes cf. incertaBenthodytes cf. incerta; scientificName: Benthodytes incertaBenthodytes incerta; kingdom: AnimaliaAnimalia; phylum: EchinodermataEchinodermata; class: HolothuroideaHolothuroidea; order: ElasipodidaElasipodida; family: PsychropotidaePsychropotidae; genus: BenthodytesBenthodytes; taxonRank: species; scientificNameAuthorship: Ludwig, 1893; **Location:** waterBody: Pacific Ocean; stateProvince: Clarion-Clipperton Zone; locality: Eastern Clarion-Clipperton Zone; verbatimLocality: Site EPIRB; maximumDepthInMeters: 3909; locationRemarks: RV Melville Cruise MV1313; decimalLatitude: 13.6785; decimalLongitude: -114.4064; geodeticDatum: WGS84; coordinateUncertaintyInMeters: 25; **Identification:** identifiedBy: Antonina Kremenetskaia, David L Pawson, Diva J Amon, Amanda F Ziegler; dateIdentified: 2014; identificationRemarks: Identified only from imagery; identificationQualifier: cf.; **Event:** samplingProtocol: Remotely Operated Vehicle; eventDate: 2013-10-23; eventTime: 11:18; habitat: Abyssal polymetallic-nodule field; fieldNumber: Dive 7 (RV07); **Record Level:** language: en; institutionCode: UHM; datasetName: ABYSSLINE; basisOfRecord: HumanObservation
- **Type status:** Other material. **Occurrence:** recordedBy: Diva J Amon, Amanda F Ziegler; individualCount: 1; lifeStage: Adult; behavior: On seafloor; occurrenceStatus: present; preparations: Imaged only; associatedReferences: 10.1038/srep30492Amon DJ, Ziegler AF, Dahlgren TG, Glover AG, Goineau A, Gooday AJ, Wiklund H, Smith CR. Insights into the abundance and diversity of abyssal megafauna in a polymetallic-nodule region in the eastern Clarion-Clipperton Zone. Scientific Reports. 2016;6. doi: 10.1038/srep30492; **Taxon:** taxonConceptID: Benthodytes cf. incertaBenthodytes cf. incerta; scientificName: Benthodytes incertaBenthodytes incerta; kingdom: AnimaliaAnimalia; phylum: EchinodermataEchinodermata; class: HolothuroideaHolothuroidea; order: ElasipodidaElasipodida; family: PsychropotidaePsychropotidae; genus: BenthodytesBenthodytes; taxonRank: species; scientificNameAuthorship: Ludwig, 1893; **Location:** waterBody: Pacific Ocean; stateProvince: Clarion-Clipperton Zone; locality: UK Seabed Resources Ltd exploration contract area (UK-1); verbatimLocality: UK-1 Stratum A; maximumDepthInMeters: 4032; locationRemarks: RV Melville Cruise MV1313; decimalLatitude: 13.8628; decimalLongitude: -116.5485; geodeticDatum: WGS84; coordinateUncertaintyInMeters: 25; **Identification:** identifiedBy: Antonina Kremenetskaia, David L Pawson, Diva J Amon, Amanda F Ziegler; dateIdentified: 2014; identificationRemarks: Identified only from imagery; identificationQualifier: cf.; **Event:** samplingProtocol: Remotely Operated Vehicle; eventDate: 2013-10-21; eventTime: 4:59; habitat: Abyssal polymetallic-nodule field; fieldNumber: Dive 6 (RV06); **Record Level:** language: en; institutionCode: UHM; datasetName: ABYSSLINE; basisOfRecord: HumanObservation
- **Type status:** Other material. **Occurrence:** recordedBy: Diva J Amon, Amanda F Ziegler; individualCount: 1; lifeStage: Adult; behavior: On seafloor; occurrenceStatus: present; preparations: Imaged only; associatedReferences: 10.1038/srep30492Amon DJ, Ziegler AF, Dahlgren TG, Glover AG, Goineau A, Gooday AJ, Wiklund H, Smith CR. Insights into the abundance and diversity of abyssal megafauna in a polymetallic-nodule region in the eastern Clarion-Clipperton Zone. Scientific Reports. 2016;6. doi: 10.1038/srep30492; **Taxon:** taxonConceptID: Benthodytes cf. incertaBenthodytes cf. incerta; scientificName: Benthodytes incertaBenthodytes incerta; kingdom: AnimaliaAnimalia; phylum: EchinodermataEchinodermata; class: HolothuroideaHolothuroidea; order: ElasipodidaElasipodida; family: PsychropotidaePsychropotidae; genus: BenthodytesBenthodytes; taxonRank: species; scientificNameAuthorship: Ludwig, 1893; **Location:** waterBody: Pacific Ocean; stateProvince: Clarion-Clipperton Zone; locality: UK Seabed Resources Ltd exploration contract area (UK-1); verbatimLocality: UK-1 Stratum A; maximumDepthInMeters: 4030; locationRemarks: RV Melville Cruise MV1313; decimalLatitude: 13.8624; decimalLongitude: -116.5484; geodeticDatum: WGS84; coordinateUncertaintyInMeters: 25; **Identification:** identifiedBy: Antonina Kremenetskaia, David L Pawson, Diva J Amon, Amanda F Ziegler; dateIdentified: 2014; identificationRemarks: Identified only from imagery; identificationQualifier: cf.; **Event:** samplingProtocol: Remotely Operated Vehicle; eventDate: 2013-10-21; eventTime: 4:54; habitat: Abyssal polymetallic-nodule field; fieldNumber: Dive 6 (RV06); **Record Level:** language: en; institutionCode: UHM; datasetName: ABYSSLINE; basisOfRecord: HumanObservation

##### Notes

Fig. 54

#### Benthodytes cf. typica

Théel, 1882

##### Materials

- **Type status:** Other material. **Occurrence:** recordedBy: Diva J Amon, Amanda F Ziegler; individualCount: 1; lifeStage: Adult; behavior: On seafloor; occurrenceStatus: present; preparations: Imaged only; associatedReferences: 10.1038/srep30492Amon DJ, Ziegler AF, Dahlgren TG, Glover AG, Goineau A, Gooday AJ, Wiklund H, Smith CR. Insights into the abundance and diversity of abyssal megafauna in a polymetallic-nodule region in the eastern Clarion-Clipperton Zone. Scientific Reports. 2016;6. doi: 10.1038/srep30492; **Taxon:** taxonConceptID: Benthodytes cf. typicaBenthodytes cf. typica; scientificName: Benthodytes typicaBenthodytes typica; kingdom: AnimaliaAnimalia; phylum: EchinodermataEchinodermata; class: HolothuroideaHolothuroidea; order: ElasipodidaElasipodida; family: PsychropotidaePsychropotidae; genus: BenthodytesBenthodytes; taxonRank: species; scientificNameAuthorship: Théel, 1882; **Location:** waterBody: Pacific Ocean; stateProvince: Clarion-Clipperton Zone; locality: UK Seabed Resources Ltd exploration contract area (UK-1); verbatimLocality: UK-1 Stratum A; maximumDepthInMeters: 4063; locationRemarks: RV Melville Cruise MV1313; decimalLatitude: 13.96301; decimalLongitude: -116.5513; geodeticDatum: WGS84; coordinateUncertaintyInMeters: 25; **Identification:** identifiedBy: Antonina Kremenetskaia, David L Pawson, Diva J Amon, Amanda F Ziegler; dateIdentified: 2014; identificationRemarks: Identified only from imagery; identificationQualifier: cf.; **Event:** samplingProtocol: Remotely Operated Vehicle; eventDate: 2013-10-15; eventTime: 22:37; habitat: Abyssal polymetallic-nodule field; fieldNumber: Dive 3 (RV03); **Record Level:** language: en; institutionCode: UHM; datasetName: ABYSSLINE; basisOfRecord: HumanObservation
- **Type status:** Other material. **Occurrence:** recordedBy: Diva J Amon, Amanda F Ziegler; individualCount: 1; lifeStage: Adult; behavior: On seafloor; occurrenceStatus: present; preparations: Imaged only; associatedReferences: 10.1038/srep30492Amon DJ, Ziegler AF, Dahlgren TG, Glover AG, Goineau A, Gooday AJ, Wiklund H, Smith CR. Insights into the abundance and diversity of abyssal megafauna in a polymetallic-nodule region in the eastern Clarion-Clipperton Zone. Scientific Reports. 2016;6. doi: 10.1038/srep30492; **Taxon:** taxonConceptID: Benthodytes cf. typicaBenthodytes cf. typica; scientificName: Benthodytes typicaBenthodytes typica; kingdom: AnimaliaAnimalia; phylum: EchinodermataEchinodermata; class: HolothuroideaHolothuroidea; order: ElasipodidaElasipodida; family: PsychropotidaePsychropotidae; genus: BenthodytesBenthodytes; taxonRank: species; scientificNameAuthorship: Théel, 1882; **Location:** waterBody: Pacific Ocean; stateProvince: Clarion-Clipperton Zone; locality: UK Seabed Resources Ltd exploration contract area (UK-1); verbatimLocality: UK-1 Stratum A; maximumDepthInMeters: 4060; locationRemarks: RV Melville Cruise MV1313; decimalLatitude: 13.9621; decimalLongitude: -116.5523; geodeticDatum: WGS84; coordinateUncertaintyInMeters: 25; **Identification:** identifiedBy: Antonina Kremenetskaia, David L Pawson, Diva J Amon, Amanda F Ziegler; dateIdentified: 2014; identificationRemarks: Identified only from imagery; identificationQualifier: cf.; **Event:** samplingProtocol: Remotely Operated Vehicle; eventDate: 2013-10-16; eventTime: 4:17; habitat: Abyssal polymetallic-nodule field; fieldNumber: Dive 3 (RV03); **Record Level:** language: en; institutionCode: UHM; datasetName: ABYSSLINE; basisOfRecord: HumanObservation
- **Type status:** Other material. **Occurrence:** catalogNumber: AB01-RV03-CS-04; recordNumber: AB01-RV03-CS-04; NHM216; recordedBy: Diva J Amon, Amanda F Ziegler; individualCount: 1; lifeStage: Adult; behavior: On seafloor; occurrenceStatus: present; preparations: tissue and DNA voucher stored in 80% non-denatured ethanol aqueous solution and remainder of animal preserved in 4% formaldehyde; otherCatalogNumbers: d0062182-89dc-4deb-b746-688289783b5f; 5023498; associatedReferences: 10.1038/srep30492Echinodermata10.3897/BDJ.4.e7251Amon DJ, Ziegler AF, Dahlgren TG, Glover AG, Goineau A, Gooday AJ, Wiklund H, Smith CR. Insights into the abundance and diversity of abyssal megafauna in a polymetallic-nodule region in the eastern Clarion-Clipperton Zone. Scientific Reports. 2016;6. doi: 10.1038/srep30492 | Glover AG, Wiklund H, Rabone M, Amon DJ, Smith CR, O'Hara T, Mah CL, Dahlgren TG. Abyssal fauna of the UK-1 polymetallic nodule exploration claim, Clarion-Clipperton Zone, central Pacific Ocean: Echinodermata. Biodiversity data journal. 2016(4). doi: 10.3897/BDJ.4.e7251; associatedSequences: http://www.ncbi.nlm.nih.gov/nuccore/KU519546KU519513http://www.ncbi.nlm.nih.gov/nuccore/KU519546 | KU519513; **Taxon:** taxonConceptID: Benthodytes cf. typicaBenthodytes cf. typica; scientificName: Benthodytes typicaBenthodytes typica; kingdom: AnimaliaAnimalia; phylum: EchinodermataEchinodermata; class: HolothuroideaHolothuroidea; order: ElasipodidaElasipodida; family: PsychropotidaePsychropotidae; genus: BenthodytesBenthodytes; taxonRank: species; scientificNameAuthorship: Théel, 1882; **Location:** waterBody: Pacific Ocean; stateProvince: Clarion-Clipperton Zone; locality: UK Seabed Resources Ltd exploration contract area (UK-1); verbatimLocality: UK-1 Stratum A; maximumDepthInMeters: 4063; locationRemarks: RV Melville Cruise MV1313; decimalLatitude: 13.9629; decimalLongitude: -116.5513; geodeticDatum: WGS84; coordinateUncertaintyInMeters: 25; **Identification:** identifiedBy: Antonina Kremenetskaia, David L Pawson, Diva J Amon, Amanda F Ziegler, Adrian Glover, Helena Wiklund, Thomas Dahlgren; dateIdentified: 2014; identificationRemarks: Identified by morphology and DNA of collected specimen; identificationQualifier: cf.; **Event:** samplingProtocol: Remotely Operated Vehicle; eventDate: 2013-10-16; eventTime: 6:14; habitat: Abyssal polymetallic-nodule field; fieldNumber: Dive 3 (RV03); **Record Level:** language: en; institutionCode: UHM; datasetName: ABYSSLINE; basisOfRecord: PreservedSpecimen

##### Notes

Fig. 55

#### Psychropotes

Théel, 1882

#### Psychropotes cf. semperiana

Théel, 1882

Psychropotes cf. semperiana In the “Atlas of Abyssal Megafauna Morphotypes of the Clarion-Clipperton Fracture Zone” created for the ISA (http://ccfzatlas.com/), this morphospecies is listed as "Psychropotes cf. semperiana".

##### Materials

- **Type status:** Other material. **Occurrence:** catalogNumber: AB01-RV03-CS05; recordNumber: AB01-RV03-CS05; NHM220; recordedBy: Diva J Amon, Amanda F Ziegler; individualCount: 1; lifeStage: Adult; behavior: On seafloor; occurrenceStatus: present; preparations: tissue and DNA voucher stored in 80% non-denatured ethanol aqueous solution and remainder of animal preserved in 4% formaldehyde; otherCatalogNumbers: 38c16bec-7bf9-4c2b-b862-5da460ba6c0c; 5023502; associatedReferences: 10.1038/srep30492Echinodermata10.3897/BDJ.4.e7251Amon DJ, Ziegler AF, Dahlgren TG, Glover AG, Goineau A, Gooday AJ, Wiklund H, Smith CR. Insights into the abundance and diversity of abyssal megafauna in a polymetallic-nodule region in the eastern Clarion-Clipperton Zone. Scientific Reports. 2016;6. doi: 10.1038/srep30492 | Glover AG, Wiklund H, Rabone M, Amon DJ, Smith CR, O'Hara T, Mah CL, Dahlgren TG. Abyssal fauna of the UK-1 polymetallic nodule exploration claim, Clarion-Clipperton Zone, central Pacific Ocean: Echinodermata. Biodiversity data journal. 2016(4). doi: 10.3897/BDJ.4.e7251; associatedSequences: http://www.ncbi.nlm.nih.gov/nuccore/KU519526http://www.ncbi.nlm.nih.gov/nuccore/KU519526; **Taxon:** taxonConceptID: Psychropotes semperianaPsychropotes semperiana; scientificName: Psychropotes semperianaPsychropotes semperiana; kingdom: AnimaliaAnimalia; phylum: EchinodermataEchinodermata; class: HolothuroideaHolothuroidea; order: ElasipodidaElasipodida; family: PsychropotidaePsychropotidae; genus: PsychropotesPsychropotes; taxonRank: species; scientificNameAuthorship: Théel, 1882; **Location:** waterBody: Pacific Ocean; stateProvince: Clarion-Clipperton Zone; locality: UK Seabed Resources Ltd exploration contract area (UK-1); verbatimLocality: UK-1 Stratum A; maximumDepthInMeters: 4062; locationRemarks: RV Melville Cruise MV1313; decimalLatitude: 13.9628; decimalLongitude: -116.5509; geodeticDatum: WGS84; coordinateUncertaintyInMeters: 25; **Identification:** identifiedBy: Antonina Kremenetskaia, David L Pawson, Diva J Amon, Amanda F Ziegler, Adrian Glover, Helena Wiklund, Thomas Dahlgren; dateIdentified: 2014; identificationRemarks: Identified by morphology and DNA of collected specimen; **Event:** samplingProtocol: Remotely Operated Vehicle; eventDate: 2013-10-16; eventTime: 4:38; habitat: Abyssal polymetallic-nodule field; fieldNumber: Dive 3 (RV03); **Record Level:** language: en; institutionCode: UHM; datasetName: ABYSSLINE; basisOfRecord: PreservedSpecimen
- **Type status:** Other material. **Occurrence:** recordedBy: Diva J Amon, Amanda F Ziegler; individualCount: 1; lifeStage: Adult; behavior: On seafloor; occurrenceStatus: present; preparations: Imaged only; **Taxon:** taxonConceptID: Psychropotes cf. semperianaPsychropotes cf. semperiana; scientificName: Psychropotes semperianaPsychropotes semperiana; kingdom: AnimaliaAnimalia; phylum: EchinodermataEchinodermata; class: HolothuroideaHolothuroidea; order: ElasipodidaElasipodida; family: PsychropotidaePsychropotidae; genus: PsychropotesPsychropotes; taxonRank: species; scientificNameAuthorship: Théel, 1882; **Location:** waterBody: Pacific Ocean; stateProvince: Clarion-Clipperton Zone; locality: UK Seabed Resources Ltd exploration contract area (UK-1); verbatimLocality: UK-1 Stratum B; maximumDepthInMeters: 4170; locationRemarks: RV Thompson Cruise TN319; decimalLatitude: 12.37503; decimalLongitude: -116.5249; geodeticDatum: WGS84; coordinateUncertaintyInMeters: 25; **Identification:** identifiedBy: Antonina Kremenetskaia, David L Pawson, Diva J Amon, Amanda F Ziegler; dateIdentified: 2015; identificationRemarks: Identified only from imagery; identificationQualifier: cf.; **Event:** samplingProtocol: Autonomous Underwater Vehicle; eventDate: 2015-02-19; eventTime: 0:31; habitat: Abyssal polymetallic-nodule field; fieldNumber: Dive 1 (AV01); **Record Level:** language: en; institutionCode: UHM; datasetName: ABYSSLINE; basisOfRecord: HumanObservation
- **Type status:** Other material. **Occurrence:** recordedBy: Diva J Amon, Amanda F Ziegler; individualCount: 1; lifeStage: Adult; behavior: On seafloor; occurrenceStatus: present; preparations: Imaged only; **Taxon:** taxonConceptID: Psychropotes cf. semperianaPsychropotes cf. semperiana; scientificName: Psychropotes semperianaPsychropotes semperiana; kingdom: AnimaliaAnimalia; phylum: EchinodermataEchinodermata; class: HolothuroideaHolothuroidea; order: ElasipodidaElasipodida; family: PsychropotidaePsychropotidae; genus: PsychropotesPsychropotes; taxonRank: species; scientificNameAuthorship: Théel, 1882; **Location:** waterBody: Pacific Ocean; stateProvince: Clarion-Clipperton Zone; locality: UK Seabed Resources Ltd exploration contract area (UK-1); verbatimLocality: UK-1 Stratum B; maximumDepthInMeters: 4184; locationRemarks: RV Thompson Cruise TN319; decimalLatitude: 12.5687; decimalLongitude: -116.7383; geodeticDatum: WGS84; coordinateUncertaintyInMeters: 25; **Identification:** identifiedBy: Antonina Kremenetskaia, David L Pawson, Diva J Amon, Amanda F Ziegler; dateIdentified: 2015; identificationRemarks: Identified only from imagery; identificationQualifier: cf.; **Event:** samplingProtocol: Autonomous Underwater Vehicle; eventDate: 2015-03-09; eventTime: 7:36; habitat: Abyssal polymetallic-nodule field; fieldNumber: Dive 6 (AV06); **Record Level:** language: en; institutionCode: UHM; datasetName: ABYSSLINE; basisOfRecord: HumanObservation
- **Type status:** Other material. **Occurrence:** recordedBy: Diva J Amon, Amanda F Ziegler; individualCount: 1; lifeStage: Adult; behavior: On seafloor; occurrenceStatus: present; preparations: Imaged only; **Taxon:** taxonConceptID: Psychropotes cf. semperianaPsychropotes cf. semperiana; scientificName: Psychropotes semperianaPsychropotes semperiana; kingdom: AnimaliaAnimalia; phylum: EchinodermataEchinodermata; class: HolothuroideaHolothuroidea; order: ElasipodidaElasipodida; family: PsychropotidaePsychropotidae; genus: PsychropotesPsychropotes; taxonRank: species; scientificNameAuthorship: Théel, 1882; **Location:** waterBody: Pacific Ocean; stateProvince: Clarion-Clipperton Zone; locality: UK Seabed Resources Ltd exploration contract area (UK-1); verbatimLocality: UK-1 Stratum B; maximumDepthInMeters: 4212; locationRemarks: RV Thompson Cruise TN319; decimalLatitude: 12.5735; decimalLongitude: -116.7334; geodeticDatum: WGS84; coordinateUncertaintyInMeters: 25; **Identification:** identifiedBy: Antonina Kremenetskaia, David L Pawson, Diva J Amon, Amanda F Ziegler; dateIdentified: 2015; identificationRemarks: Identified only from imagery; identificationQualifier: cf.; **Event:** samplingProtocol: Autonomous Underwater Vehicle; eventDate: 2015-03-09; eventTime: 7:45; habitat: Abyssal polymetallic-nodule field; fieldNumber: Dive 6 (AV06); **Record Level:** language: en; institutionCode: UHM; datasetName: ABYSSLINE; basisOfRecord: HumanObservation

##### Notes

Fig. 56

#### Psychropotes cf. verrucosa

Ludwig, 1893

Psychropotes cf. verrucosa In the “Atlas of Abyssal Megafauna Morphotypes of the Clarion-Clipperton Fracture Zone” created for the ISA (http://ccfzatlas.com/), this morphospecies is listed as "*Psychropotes verrucosa* Ludwig, 1893".

##### Materials

- **Type status:** Other material. **Occurrence:** recordedBy: Diva J Amon, Amanda F Ziegler; individualCount: 1; lifeStage: Adult; behavior: On seafloor; occurrenceStatus: present; preparations: Imaged only; associatedReferences: 10.1038/srep30492Amon DJ, Ziegler AF, Dahlgren TG, Glover AG, Goineau A, Gooday AJ, Wiklund H, Smith CR. Insights into the abundance and diversity of abyssal megafauna in a polymetallic-nodule region in the eastern Clarion-Clipperton Zone. Scientific Reports. 2016;6. doi: 10.1038/srep30492; **Taxon:** taxonConceptID: Psychropotes cf. verrucosaPsychropotes cf. verrucosa; scientificName: Psychropotes verrucosaPsychropotes verrucosa; kingdom: AnimaliaAnimalia; phylum: EchinodermataEchinodermata; class: HolothuroideaHolothuroidea; order: ElasipodidaElasipodida; family: PsychropotidaePsychropotidae; genus: PsychropotesPsychropotes; taxonRank: species; scientificNameAuthorship: Ludwig, 1893; **Location:** waterBody: Pacific Ocean; stateProvince: Clarion-Clipperton Zone; locality: UK Seabed Resources Ltd exploration contract area (UK-1); verbatimLocality: UK-1 Stratum A; maximumDepthInMeters: 4023; locationRemarks: RV Melville Cruise MV1313; decimalLatitude: 13.8569; decimalLongitude: -116.5474; geodeticDatum: WGS84; coordinateUncertaintyInMeters: 25; **Identification:** identifiedBy: Antonina Kremenetskaia, David L Pawson, Diva J Amon, Amanda F Ziegler; dateIdentified: 2014; identificationRemarks: Identified only from imagery; identificationQualifier: cf.; **Event:** samplingProtocol: Remotely Operated Vehicle; eventDate: 2013-10-21; eventTime: 2:49; habitat: Abyssal polymetallic-nodule field; fieldNumber: Dive 6 (RV06); **Record Level:** language: en; institutionCode: UHM; datasetName: ABYSSLINE; basisOfRecord: HumanObservation

##### Notes

Fig. 57

#### Ophiuroidea

Gray, 1840

#### cf. Ophiuroidea morphospecies

##### Materials

- **Type status:** Other material. **Occurrence:** recordedBy: Diva J Amon, Amanda F Ziegler; individualCount: 1; lifeStage: Adult; behavior: On rock; occurrenceStatus: present; preparations: Imaged only; **Taxon:** taxonConceptID: Ophiuroideacf. Ophiuroidea morphospecies; scientificName: OphiuroideaOphiuroidea sp.; kingdom: AnimaliaAnimalia; phylum: EchinodermataEchinodermata; class: OphiuroideaOphiuroidea; taxonRank: class; scientificNameAuthorship: Gray, 1840; **Location:** waterBody: Pacific Ocean; stateProvince: Clarion-Clipperton Zone; locality: UK Seabed Resources Ltd exploration contract area (UK-1); verbatimLocality: UK-1 Stratum B; maximumDepthInMeters: 4224; locationRemarks: RV Thompson Cruise TN319; decimalLatitude: 12.5789; decimalLongitude: -116.6918; geodeticDatum: WGS84; coordinateUncertaintyInMeters: 25; **Identification:** identifiedBy: Tim O'Hara, Diva J, Amon, Amanda F Ziegler; dateIdentified: 2015; identificationRemarks: Identified only from imagery; identificationQualifier: cf.; **Event:** samplingProtocol: Autonomous Underwater Vehicle; eventDate: 2015-03-09; eventTime: 5:12; habitat: Abyssal polymetallic-nodule field; fieldNumber: Dive 6 (AV06); **Record Level:** language: en; institutionCode: UHM; datasetName: ABYSSLINE; basisOfRecord: HumanObservation
- **Type status:** Other material. **Occurrence:** recordedBy: Diva J Amon, Amanda F Ziegler; individualCount: 1; lifeStage: Adult; behavior: On seafloor; occurrenceStatus: present; preparations: Imaged only; **Taxon:** taxonConceptID: Ophiuroideacf. Ophiuroidea morphospecies; scientificName: OphiuroideaOphiuroidea sp.; kingdom: AnimaliaAnimalia; phylum: EchinodermataEchinodermata; class: OphiuroideaOphiuroidea; taxonRank: class; scientificNameAuthorship: Gray, 1840; **Location:** waterBody: Pacific Ocean; stateProvince: Clarion-Clipperton Zone; locality: UK Seabed Resources Ltd exploration contract area (UK-1); verbatimLocality: UK-1 Stratum B; maximumDepthInMeters: 4255; locationRemarks: RV Thompson Cruise TN319; decimalLatitude: 12.5021; decimalLongitude: -116.64896; geodeticDatum: WGS84; coordinateUncertaintyInMeters: 25; **Identification:** identifiedBy: Tim O'Hara, Diva J, Amon, Amanda F Ziegler; dateIdentified: 2015; identificationRemarks: Identified only from imagery; identificationQualifier: cf.; **Event:** samplingProtocol: Autonomous Underwater Vehicle; eventDate: 2015-03-18; eventTime: 8:36; habitat: Abyssal polymetallic-nodule field; fieldNumber: Dive 9 (AV09); **Record Level:** language: en; institutionCode: UHM; datasetName: ABYSSLINE; basisOfRecord: HumanObservation
- **Type status:** Other material. **Occurrence:** recordedBy: Diva J Amon, Amanda F Ziegler; individualCount: 1; lifeStage: Adult; behavior: On seafloor; occurrenceStatus: present; preparations: Imaged only; **Taxon:** taxonConceptID: Ophiuroideacf. Ophiuroidea morphospecies; scientificName: OphiuroideaOphiuroidea sp.; kingdom: AnimaliaAnimalia; phylum: EchinodermataEchinodermata; class: OphiuroideaOphiuroidea; taxonRank: class; scientificNameAuthorship: Gray, 1840; **Location:** waterBody: Pacific Ocean; stateProvince: Clarion-Clipperton Zone; locality: UK Seabed Resources Ltd exploration contract area (UK-1); verbatimLocality: UK-1 Stratum B; maximumDepthInMeters: 4248; locationRemarks: RV Thompson Cruise TN319; decimalLatitude: 12.5046; decimalLongitude: -116.6393; geodeticDatum: WGS84; coordinateUncertaintyInMeters: 25; **Identification:** identifiedBy: Tim O'Hara, Diva J, Amon, Amanda F Ziegler; dateIdentified: 2015; identificationRemarks: Identified only from imagery; identificationQualifier: cf.; **Event:** samplingProtocol: Autonomous Underwater Vehicle; eventDate: 2015-03-18; eventTime: 9:25; habitat: Abyssal polymetallic-nodule field; fieldNumber: Dive 9 (AV09); **Record Level:** language: en; institutionCode: UHM; datasetName: ABYSSLINE; basisOfRecord: HumanObservation

##### Notes

Fig. 58

#### Ophiurida

Müller & Troschel, 1840

#### Amphiuridae

Ljungman, 1867

#### Amphioplus

Verrill, 1899

#### Amphioplus (Unioplus) daleus

Lyman, 1879

##### Materials

- **Type status:** Other material. **Occurrence:** catalogNumber: AB01-MC10-CS-15; recordNumber: AB01-MC10-CS-15; NHM447; recordedBy: Diva J Amon, Amanda F Ziegler; individualCount: 1; lifeStage: Adult; behavior: On seafloor; occurrenceStatus: present; preparations: tissue and DNA voucher stored in 80% non-denatured ethanol aqueous solution and remainder of animal preserved in 4% formaldehyde; otherCatalogNumbers: 15e6ddc7-3ca7-453c-bba5-f84888716505; associatedReferences: 10.1038/srep30492Echinodermata10.3897/BDJ.4.e7251Amon DJ, Ziegler AF, Dahlgren TG, Glover AG, Goineau A, Gooday AJ, Wiklund H, Smith CR. Insights into the abundance and diversity of abyssal megafauna in a polymetallic-nodule region in the eastern Clarion-Clipperton Zone. Scientific Reports. 2016;6. doi: 10.1038/srep30492 | Glover AG, Wiklund H, Rabone M, Amon DJ, Smith CR, O'Hara T, Mah CL, Dahlgren TG. Abyssal fauna of the UK-1 polymetallic nodule exploration claim, Clarion-Clipperton Zone, central Pacific Ocean: Echinodermata. Biodiversity data journal. 2016(4). doi: 10.3897/BDJ.4.e7251; associatedSequences: http://www.ncbi.nlm.nih.gov/nuccore/KU519545KU519511KU519529http://www.ncbi.nlm.nih.gov/nuccore/KU519545 | KU519511 | KU519529; **Taxon:** taxonConceptID: Amphioplus (Unioplus) daleusAmphioplus (Unioplus) daleus; scientificName: Amphioplus (Unioplus) daleusAmphioplus (Unioplus) daleus; kingdom: AnimaliaAnimalia; phylum: EchinodermataEchinodermata; class: OphiuroideaOphiuroidea; order: OphiuridaOphiurida; family: AmphiuridaeAmphiuridae; genus: AmphioplusAmphioplus; taxonRank: species; scientificNameAuthorship: Lyman, 1879; **Location:** waterBody: Pacific Ocean; stateProvince: Clarion-Clipperton Zone; locality: UK Seabed Resources Ltd exploration contract area (UK-1); verbatimLocality: UK-1 Stratum A; maximumDepthInMeters: 4053; locationRemarks: RV Melville Cruise MV1313; decimalLatitude: 13.8634; decimalLongitude: -116.5467; geodeticDatum: WGS84; coordinateUncertaintyInMeters: 50; **Identification:** identifiedBy: Tim O'Hara, Diva J Amon, Amanda F Ziegler, Adrian Glover, Helena Wiklund, Thomas Dahlgren; dateIdentified: 2014; identificationRemarks: Identified by morphology and DNA of collected specimen; identificationQualifier: cf.; **Event:** samplingProtocol: Megacorer; eventDate: 2013-10-21; eventTime: 8:48; habitat: Abyssal polymetallic-nodule field; fieldNumber: Megacorer 10 (MC10); **Record Level:** language: en; institutionCode: UHM; datasetName: ABYSSLINE; basisOfRecord: PreservedSpecimen
- **Type status:** Other material. **Occurrence:** catalogNumber: AB02-BC19-CS-30; recordNumber: AB02-BC19-CS-30; NHM1591; recordedBy: Diva J Amon, Amanda F Ziegler; individualCount: 1; lifeStage: Adult; behavior: Found underneath a nodule; occurrenceStatus: present; preparations: tissue and DNA voucher stored in 80% non-denatured ethanol aqueous solution and remainder of animal preserved in 4% formaldehyde; **Taxon:** taxonConceptID: Amphioplus (Unioplus) daleusAmphioplus (Unioplus) daleus; scientificName: Amphioplus (Unioplus) daleusAmphioplus (Unioplus) daleus; kingdom: AnimaliaAnimalia; phylum: EchinodermataEchinodermata; class: OphiuroideaOphiuroidea; order: OphiuridaOphiurida; family: AmphiuridaeAmphiuridae; genus: AmphioplusAmphioplus; taxonRank: species; scientificNameAuthorship: Lyman, 1879; **Location:** waterBody: Pacific Ocean; stateProvince: Clarion-Clipperton Zone; locality: UK Seabed Resources Ltd exploration contract area (UK-1); verbatimLocality: UK-1 Stratum B; maximumDepthInMeters: 4237; locationRemarks: RV Thompson Cruise TN319; decimalLatitude: 12.5212; decimalLongitude: -116.6982; geodeticDatum: WGS84; coordinateUncertaintyInMeters: 50; **Identification:** identifiedBy: Tim O'Hara, Diva J Amon, Amanda F Ziegler, Adrian Glover, Helena Wiklund, Thomas Dahlgren; dateIdentified: 2015; identificationRemarks: Identified by morphology and DNA of collected specimen; identificationQualifier: cf.; **Event:** samplingProtocol: Box corer; eventDate: 2015-03-08; eventTime: 8:04; habitat: Abyssal polymetallic-nodule field; fieldNumber: Box corer 19 (BC19); **Record Level:** language: en; institutionCode: UHM; datasetName: ABYSSLINE; basisOfRecord: PreservedSpecimen

##### Notes

Fig. 59

#### Ophiacanthidae

Ljungman, 1867

#### Ophiacantha

Müller & Troschel, 1842

#### cf. Ophiacantha morphospecies

##### Materials

- **Type status:** Other material. **Occurrence:** recordedBy: Diva J Amon, Amanda F Ziegler; individualCount: 1; lifeStage: Adult; behavior: Frequently observed on sponge stalks, rocks and seafloor; occurrenceStatus: present; preparations: Imaged only; associatedReferences: 10.1038/srep30492Amon DJ, Ziegler AF, Dahlgren TG, Glover AG, Goineau A, Gooday AJ, Wiklund H, Smith CR. Insights into the abundance and diversity of abyssal megafauna in a polymetallic-nodule region in the eastern Clarion-Clipperton Zone. Scientific Reports. 2016;6. doi: 10.1038/srep30492; **Taxon:** taxonConceptID: *Ophiacantha*cf. *Ophiacantha* morphospecies; scientificName: *OphiacanthaOphiacantha* sp.; kingdom: AnimaliaAnimalia; phylum: EchinodermataEchinodermata; class: OphiuroideaOphiuroidea; order: OphiuridaOphiurida; family: OphiacanthidaeOphiacanthidae; genus: OphiacanthaOphiacantha; taxonRank: genus; scientificNameAuthorship: Muller & Troschel, 1842; **Location:** waterBody: Pacific Ocean; stateProvince: Clarion-Clipperton Zone; locality: UK Seabed Resources Ltd exploration contract area (UK-1); verbatimLocality: UK-1 Stratum A; maximumDepthInMeters: 3935; locationRemarks: RV Melville Cruise MV1313; decimalLatitude: 13.8604; decimalLongitude: -116.5484; geodeticDatum: WGS84; coordinateUncertaintyInMeters: 25; **Identification:** identifiedBy: Tim O'Hara, Diva J, Amon, Amanda F Ziegler; dateIdentified: 2014; identificationRemarks: Identified only from imagery; identificationQualifier: cf.; **Event:** samplingProtocol: Remotely Operated Vehicle; eventDate: 2013-10-21; eventTime: 4:27; habitat: Abyssal polymetallic-nodule field; fieldNumber: Dive 6 (RV06); **Record Level:** language: en; institutionCode: UHM; datasetName: ABYSSLINE; basisOfRecord: HumanObservation
- **Type status:** Other material. **Occurrence:** recordedBy: Diva J Amon, Amanda F Ziegler; individualCount: 1; lifeStage: Adult; behavior: Frequently observed on sponge stalks, rocks and seafloor; occurrenceStatus: present; preparations: Imaged only; associatedReferences: 10.1038/srep30492Amon DJ, Ziegler AF, Dahlgren TG, Glover AG, Goineau A, Gooday AJ, Wiklund H, Smith CR. Insights into the abundance and diversity of abyssal megafauna in a polymetallic-nodule region in the eastern Clarion-Clipperton Zone. Scientific Reports. 2016;6. doi: 10.1038/srep30492; **Taxon:** taxonConceptID: *Ophiacantha*cf. *Ophiacantha* morphospecies; scientificName: *OphiacanthaOphiacantha* sp.; kingdom: AnimaliaAnimalia; phylum: EchinodermataEchinodermata; class: OphiuroideaOphiuroidea; order: OphiuridaOphiurida; family: OphiacanthidaeOphiacanthidae; genus: OphiacanthaOphiacantha; taxonRank: genus; scientificNameAuthorship: Muller & Troschel, 1842; **Location:** waterBody: Pacific Ocean; stateProvince: Clarion-Clipperton Zone; locality: UK Seabed Resources Ltd exploration contract area (UK-1); verbatimLocality: UK-1 Stratum A; maximumDepthInMeters: 4028; locationRemarks: RV Melville Cruise MV1313; decimalLatitude: 13.8631; decimalLongitude: -116.5486; geodeticDatum: WGS84; coordinateUncertaintyInMeters: 25; **Identification:** identifiedBy: Tim O'Hara, Diva J, Amon, Amanda F Ziegler; dateIdentified: 2014; identificationRemarks: Identified only from imagery; identificationQualifier: cf.; **Event:** samplingProtocol: Remotely Operated Vehicle; eventDate: 2013-10-21; eventTime: 5:02; habitat: Abyssal polymetallic-nodule field; fieldNumber: Dive 6 (RV06); **Record Level:** language: en; institutionCode: UHM; datasetName: ABYSSLINE; basisOfRecord: HumanObservation

##### Notes

Fig. 60

#### Ophiolepididae

Ljungman, 1867

#### Ophiosphalma

H.L. Clark, 1941

#### Ophiosphalma cf. glabrum

Lütken & Mortensen, 1899

Ophiosphalma cf. glabrum In the “Atlas of Abyssal Megafauna Morphotypes of the Clarion-Clipperton Fracture Zone” created for the ISA (http://ccfzatlas.com/), this morphospecies is listed as "*Ophiosphalma* morphotype".

##### Materials

- **Type status:** Other material. **Occurrence:** catalogNumber: AB01-RV05-CS-06; recordNumber: AB01-RV05-CS-06; NHM329; recordedBy: Diva J Amon, Amanda F Ziegler; individualCount: 1; lifeStage: Adult; behavior: On seafloor; occurrenceStatus: present; preparations: tissue and DNA voucher stored in 80% non-denatured ethanol aqueous solution and remainder of animal preserved in 4% formaldehyde; otherCatalogNumbers: 11948cb9-654f-4519-a654-f134380093ea; associatedReferences: 10.1038/srep30492Echinodermata10.3897/BDJ.4.e7251Amon DJ, Ziegler AF, Dahlgren TG, Glover AG, Goineau A, Gooday AJ, Wiklund H, Smith CR. Insights into the abundance and diversity of abyssal megafauna in a polymetallic-nodule region in the eastern Clarion-Clipperton Zone. Scientific Reports. 2016;6. doi: 10.1038/srep30492 | Glover AG, Wiklund H, Rabone M, Amon DJ, Smith CR, O'Hara T, Mah CL, Dahlgren TG. Abyssal fauna of the UK-1 polymetallic nodule exploration claim, Clarion-Clipperton Zone, central Pacific Ocean: Echinodermata. Biodiversity data journal. 2016(4). doi: 10.3897/BDJ.4.e7251; associatedSequences: http://www.ncbi.nlm.nih.gov/nuccore/KU519555KU519519KU519536http://www.ncbi.nlm.nih.gov/nuccore/KU519555 | KU519519 | KU519536; **Taxon:** taxonConceptID: Ophiosphalma cf. glabrumOphiosphalma cf. glabrum; scientificName: Ophiosphalma glabrumOphiosphalma glabrum; kingdom: AnimaliaAnimalia; phylum: EchinodermataEchinodermata; class: OphiuroideaOphiuroidea; order: OphiuridaOphiurida; family: OphiolepididaeOphiolepididae; genus: OphiosphalmaOphiosphalma; taxonRank: species; scientificNameAuthorship: Lutken & Mortensen, 1899; **Location:** waterBody: Pacific Ocean; stateProvince: Clarion-Clipperton Zone; locality: UK Seabed Resources Ltd exploration contract area (UK-1); verbatimLocality: UK-1 Stratum A; maximumDepthInMeters: 4075; locationRemarks: RV Melville Cruise MV1313; decimalLatitude: 13.7609; decimalLongitude: -116.4653; geodeticDatum: WGS84; coordinateUncertaintyInMeters: 25; **Identification:** identifiedBy: Tim O'Hara, Diva J Amon, Amanda F Ziegler, Adrian Glover, Helena Wiklund, Thomas Dahlgren; dateIdentified: 2014; identificationRemarks: Identified by morphology and DNA of collected specimen; identificationQualifier: cf.; **Event:** samplingProtocol: Remotely Operated Vehicle; eventDate: 2013-10-17; eventTime: 19:06; habitat: Abyssal polymetallic-nodule field; fieldNumber: Dive 5 (RV05); **Record Level:** language: en; institutionCode: UHM; datasetName: ABYSSLINE; basisOfRecord: PreservedSpecimen
- **Type status:** Other material. **Occurrence:** catalogNumber: AB01-RV05-CS-08; recordNumber: AB01-RV05-CS-08; NHM338; recordedBy: Diva J Amon, Amanda F Ziegler; individualCount: 1; lifeStage: Adult; behavior: On seafloor; occurrenceStatus: present; preparations: tissue and DNA voucher stored in 80% non-denatured ethanol aqueous solution and remainder of animal preserved in 4% formaldehyde; otherCatalogNumbers: 292bd655-83d6-440f-9668-82dfa4185b04; associatedReferences: 10.1038/srep30492Echinodermata10.3897/BDJ.4.e7251Amon DJ, Ziegler AF, Dahlgren TG, Glover AG, Goineau A, Gooday AJ, Wiklund H, Smith CR. Insights into the abundance and diversity of abyssal megafauna in a polymetallic-nodule region in the eastern Clarion-Clipperton Zone. Scientific Reports. 2016;6. doi: 10.1038/srep30492 | Glover AG, Wiklund H, Rabone M, Amon DJ, Smith CR, O'Hara T, Mah CL, Dahlgren TG. Abyssal fauna of the UK-1 polymetallic nodule exploration claim, Clarion-Clipperton Zone, central Pacific Ocean: Echinodermata. Biodiversity data journal. 2016(4). doi: 10.3897/BDJ.4.e7251; associatedSequences: http://www.ncbi.nlm.nih.gov/nuccore/KU519556http://www.ncbi.nlm.nih.gov/nuccore/KU519556; **Taxon:** taxonConceptID: Ophiosphalma cf. glabrumOphiosphalma cf. glabrum; scientificName: Ophiosphalma glabrumOphiosphalma glabrum; kingdom: AnimaliaAnimalia; phylum: EchinodermataEchinodermata; class: OphiuroideaOphiuroidea; order: OphiuridaOphiurida; family: OphiolepididaeOphiolepididae; genus: OphiosphalmaOphiosphalma; taxonRank: species; scientificNameAuthorship: Lutken & Mortensen, 1899; **Location:** waterBody: Pacific Ocean; stateProvince: Clarion-Clipperton Zone; locality: UK Seabed Resources Ltd exploration contract area (UK-1); verbatimLocality: UK-1 Stratum A; maximumDepthInMeters: 4075; locationRemarks: RV Melville Cruise MV1313; decimalLatitude: 13.7609; decimalLongitude: -116.4653; geodeticDatum: WGS84; coordinateUncertaintyInMeters: 25; **Identification:** identifiedBy: Tim O'Hara, Diva J Amon, Amanda F Ziegler, Adrian Glover, Helena Wiklund, Thomas Dahlgren; dateIdentified: 2014; identificationRemarks: Identified by morphology and DNA of collected specimen; identificationQualifier: cf.; **Event:** samplingProtocol: Remotely Operated Vehicle; eventDate: 2013-10-17; eventTime: 19:06; habitat: Abyssal polymetallic-nodule field; fieldNumber: Dive 5 (RV05); **Record Level:** language: en; institutionCode: UHM; datasetName: ABYSSLINE; basisOfRecord: PreservedSpecimen
- **Type status:** Other material. **Occurrence:** recordedBy: Diva J Amon, Amanda F Ziegler; individualCount: 1; lifeStage: Adult; behavior: On seafloor; occurrenceStatus: present; preparations: Imaged only; associatedReferences: 10.1038/srep30492Echinodermata10.3897/BDJ.4.e7251Amon DJ, Ziegler AF, Dahlgren TG, Glover AG, Goineau A, Gooday AJ, Wiklund H, Smith CR. Insights into the abundance and diversity of abyssal megafauna in a polymetallic-nodule region in the eastern Clarion-Clipperton Zone. Scientific Reports. 2016;6. doi: 10.1038/srep30492 | Glover AG, Wiklund H, Rabone M, Amon DJ, Smith CR, O'Hara T, Mah CL, Dahlgren TG. Abyssal fauna of the UK-1 polymetallic nodule exploration claim, Clarion-Clipperton Zone, central Pacific Ocean: Echinodermata. Biodiversity data journal. 2016(4). doi: 10.3897/BDJ.4.e7251; **Taxon:** taxonConceptID: Ophiosphalma cf. glabrumOphiosphalma cf. glabrum; scientificName: Ophiosphalma glabrumOphiosphalma glabrum; kingdom: AnimaliaAnimalia; phylum: EchinodermataEchinodermata; class: OphiuroideaOphiuroidea; order: OphiuridaOphiurida; family: OphiolepididaeOphiolepididae; genus: OphiosphalmaOphiosphalma; taxonRank: species; scientificNameAuthorship: Lutken & Mortensen, 1899; **Location:** waterBody: Pacific Ocean; stateProvince: Clarion-Clipperton Zone; locality: UK Seabed Resources Ltd exploration contract area (UK-1); verbatimLocality: UK-1 Stratum A; maximumDepthInMeters: 4123; locationRemarks: RV Melville Cruise MV1313; decimalLatitude: 13.8498; decimalLongitude: -116.6457; geodeticDatum: WGS84; coordinateUncertaintyInMeters: 25; **Identification:** identifiedBy: Tim O'Hara, Diva J, Amon, Amanda F Ziegler; dateIdentified: 2014; identificationRemarks: Identified only from imagery; identificationQualifier: cf.; **Event:** samplingProtocol: Remotely Operated Vehicle; eventDate: 2013-10-10; eventTime: 12:34; habitat: Abyssal polymetallic-nodule field; fieldNumber: Dive 1 (RV01); **Record Level:** language: en; institutionCode: UHM; datasetName: ABYSSLINE; basisOfRecord: HumanObservation

##### Notes

Fig. 61

#### Ophiohelidae

Perrier, 1893

#### Ophiotholia

Lyman, 1880

#### cf. Ophiotholia morphospecies

##### Materials

- **Type status:** Other material. **Occurrence:** catalogNumber: AB02-BC02-CS-02; recordNumber: AB02-BC02-CS-02; NHM524; recordedBy: Diva J Amon, Amanda F Ziegler; individualCount: 1; lifeStage: Adult; behavior: On seafloor; occurrenceStatus: present; preparations: tissue and DNA voucher stored in 80% non-denatured ethanol aqueous solution and remainder of animal preserved in 4% formaldehyde; **Taxon:** taxonConceptID: *Ophiotholia*cf. *Ophiotholia* morphospecies; scientificName: *OphiotholiaOphiotholia* sp.; kingdom: AnimaliaAnimalia; phylum: EchinodermataEchinodermata; class: OphiuroideaOphiuroidea; order: OphiuridaOphiurida; family: OphiohelidaeOphiohelidae; genus: OphiotholiaOphiotholia; taxonRank: genus; scientificNameAuthorship: Lyman, 1880; **Location:** waterBody: Pacific Ocean; stateProvince: Clarion-Clipperton Zone; locality: UK Seabed Resources Ltd exploration contract area (UK-1); verbatimLocality: UK-1 Stratum B; maximumDepthInMeters: 4158; locationRemarks: RV Thompson Cruise TN319; decimalLatitude: 12.367; decimalLongitude: -116.51695; geodeticDatum: WGS84; coordinateUncertaintyInMeters: 50; **Identification:** identifiedBy: Tim O'Hara, Diva J Amon, Amanda F Ziegler; dateIdentified: 2015; identificationRemarks: Identified by morphology; identificationQualifier: cf.; **Event:** samplingProtocol: Box corer; eventDate: 2015-02-17; eventTime: 12:12; habitat: Abyssal polymetallic-nodule field; fieldNumber: Box corer 02 (BC02); **Record Level:** language: en; institutionCode: UHM; datasetName: ABYSSLINE; basisOfRecord: PreservedSpecimen

##### Notes

Fig. 62

#### Ophiuridae

Müller & Troschel, 1840

#### Ophiotypa

Koehler, 1897

#### Ophiotypa cf. simplex

Koehler, 1897

##### Materials

- **Type status:** Other material. **Occurrence:** recordedBy: Diva J Amon, Amanda F Ziegler; individualCount: 1; lifeStage: Adult; behavior: On seafloor; occurrenceStatus: present; preparations: Imaged only; **Taxon:** taxonConceptID: Ophiotypa cf. simplexOphiotypa cf. simplex; scientificName: Ophiotypa simplexOphiotypa simplex; kingdom: AnimaliaAnimalia; phylum: EchinodermataEchinodermata; class: OphiuroideaOphiuroidea; order: OphiuridaOphiurida; family: OphiuridaeOphiuridae; genus: OphiotypaOphiotypa; taxonRank: species; scientificNameAuthorship: Koehler, 1897; **Location:** waterBody: Pacific Ocean; stateProvince: Clarion-Clipperton Zone; locality: UK Seabed Resources Ltd exploration contract area (UK-1); verbatimLocality: UK-1 Stratum A; maximumDepthInMeters: 4066; locationRemarks: RV Melville Cruise MV1313; decimalLatitude: 13.7602; decimalLongitude: -116.4678; geodeticDatum: WGS84; coordinateUncertaintyInMeters: 25; **Identification:** identifiedBy: Tim O'Hara, Diva J Amon, Amanda F Ziegler; dateIdentified: 2014; identificationRemarks: Identified only from imagery; identificationQualifier: cf.; **Event:** samplingProtocol: Remotely Operated Vehicle; eventDate: 2013-10-18; eventTime: 2:47; habitat: Abyssal polymetallic-nodule field; fieldNumber: Dive 5 (RV05); **Record Level:** language: en; institutionCode: UHM; datasetName: ABYSSLINE; basisOfRecord: HumanObservation
- **Type status:** Other material. **Occurrence:** recordedBy: Diva J Amon, Amanda F Ziegler; individualCount: 1; lifeStage: Adult; behavior: On seafloor; occurrenceStatus: present; preparations: Imaged only; **Taxon:** taxonConceptID: Ophiotypa cf. simplexOphiotypa cf. simplex; scientificName: Ophiotypa simplexOphiotypa simplex; kingdom: AnimaliaAnimalia; phylum: EchinodermataEchinodermata; class: OphiuroideaOphiuroidea; order: OphiuridaOphiurida; family: OphiuridaeOphiuridae; genus: OphiotypaOphiotypa; taxonRank: species; scientificNameAuthorship: Koehler, 1897; **Location:** waterBody: Pacific Ocean; stateProvince: Clarion-Clipperton Zone; locality: Eastern Clarion-Clipperton Zone; verbatimLocality: Site EPIRB; maximumDepthInMeters: 3952; locationRemarks: RV Melville Cruise MV1313; decimalLatitude: 13.6794; decimalLongitude: -114.4143; geodeticDatum: WGS84; coordinateUncertaintyInMeters: 25; **Identification:** identifiedBy: Tim O'Hara, Diva J Amon, Amanda F Ziegler; dateIdentified: 2014; identificationRemarks: Identified only from imagery; identificationQualifier: cf.; **Event:** samplingProtocol: Remotely Operated Vehicle; eventDate: 2013-10-23; eventTime: 9:49; habitat: Abyssal polymetallic-nodule field; fieldNumber: Dive 7 (RV07); **Record Level:** language: en; institutionCode: UHM; datasetName: ABYSSLINE; basisOfRecord: HumanObservation
- **Type status:** Other material. **Occurrence:** catalogNumber: AB02-MC25-CS76; recordNumber: AB02-MC25-CS76; NHM2083; recordedBy: Diva J. Amon, Amanda F. Ziegler; individualCount: 1; lifeStage: Adult; behavior: On seafloor; occurrenceStatus: present; preparations: tissue and DNA voucher stored in 80% non-denaturedethanol aqueous solution and remainder of animal preserved in 4% formaldehyde; **Taxon:** taxonConceptID: Ophiotypa simplexOphiotypa simplex; scientificName: Ophiotypa simplexOphiotypa simplex; kingdom: AnimaliaAnimalia; phylum: EchinodermataEchinodermata; class: OphiuroideaOphiuroidea; order: OphiuridaOphiurida; family: OphiuridaeOphiuridae; genus: OphiotypaOphiotypa; taxonRank: species; scientificNameAuthorship: Koehler, 1897; **Location:** waterBody: Pacific Ocean; stateProvince: Clarion-Clipperton Zone; locality: UK Seabed Resources Ltd exploration contract area (UK-1); verbatimLocality: UK-1 Stratum B; maximumDepthInMeters: 4224; locationRemarks: RV Thompson Cruise TN319; decimalLatitude: 12.58255; decimalLongitude: -116.6509667; geodeticDatum: WGS84; coordinateUncertaintyInMeters: 50; **Identification:** identifiedBy: Tim O'Hara, Diva J Amon, Amanda F Ziegler; dateIdentified: 2015; identificationRemarks: Identified by morphology; **Event:** samplingProtocol: Megacorer; eventDate: 2015-03-18; eventTime: 3:2; habitat: Abyssal polymetallic-nodule field; **Record Level:** language: en; institutionCode: UHM; datasetName: ABYSSLINE; basisOfRecord: HumanObservation

##### Notes

Fig. 63

## Discussion

Although many of the morphospecies included here remain taxonomically ambiguous, we provide the first image atlas of echinoderm megafauna morphospecies inhabiting the UK-1 exploration contract area and the eastern CCZ. There were 62 distinct morphospecies (13 Asteroidea, 5 Crinoidea, 9 Echinoidea, 29 Holothuroidea and 6 Ophiuroidea) observed, making it the most speciose phylum in the wider ABYSSLINE study. This is the highest species richness for one phyla ever recorded in the entire CCZ region and is even more remarkable given that this is from one exploration contract area (UK-1) and a single dive site east of the contract area. Previous studies in the CCZ have identified 38 echinoderm morphotypes (Foell and Pawson 1986), and 46 morphotypes (Tilot 2006). Bluhm and Gebruk (1999) noted 28 holothurian morphotypes from the DISCOL site in the southeastern Pacific Ocean, which is similar to the numbers observed during this study (29). The quantitative study by (Amon et al. 2016b) which utilised many of the images from AB01 included in this study reported 41 echinoderm morphotypes (the second-most speciose phylum behind Cnidaria - 48 morphotypes) from UK-1 Stratum A and EPIRB only. However, we recognise that the comparison of echinoderm species richness aross the region is only valid if sampling effort was similar or standardised (Amon et al. 2016b). This suggests that the Echinodermata are the most conspicuous, and therefore, best-characterised of all phyla occuring the CCZ.

These morphospecies represent a range of functional traits: the crinoids are sessile or semi-sessile suspension feeders, reliant on the polymetallic nodules as hard substrate, whereas most of the asteroids (excluding brisingids), echinoids, holothurians, and ophiuroids are mobile deposit feeders (Amon et al. 2016b, Vanreusel et al. 2016). Approximately half of the morphospecies in this atlas have been observed in other contract areas in the CCZ (http://ccfzatlas.com/, Bluhm and Gebruk 1999, Pawson 1983, Pawson and Foell 1986, Tilot 2006), although this may be an overestimate given the presence of cryptic species and the problems identifying megafauna from imagery, as has been experienced during studies in other poorly-explored areas (Amon et al. 2016b, Vrijenhoek 2009, Bickford et al. 2006, Linse et al. 2007). Information like this will likely be crucial to inform the future environmental management of the region.

While this image atlas has expanded the knowledge of benthic fauna in the UK-1 exploration claim area and overall CCZ, there is still a need for further high-quality imagery of fauna, and an even more dire need for physical megafaunal specimens to ground-truth the morphospecies observed in images via detailed morphological and molecular analyses. We expect that a number of the morphospecies included in this atlas may be new to science, new records, or poorly known, but this can only be confirmed when specimens are collected and analysed. Molecular analyses are especially important given the presence of cryptic species. The limited collection of voucher specimens in the CCZ thus far has severely hampered reliable estimation of species richness and species distributions and continues to be an issue. Although the taxonomic identification of preserved material is always necessary, we hope that this atlas will aid scientists by showing what these morphospecies look like in situ in their natural surroundings, as well as by providing some ecological information (e.g. feeding modes, preferred habitat etc.). This information will be important in estimating the human impact on this ecosystem. Furthermore, the appearance of morphospecies captured in situ in images can drastically differ from that of collected or preserved material, especially when relatively rudimentary collection equipment (trawls, dredges etc.) are used. As mentioned in Amon et al. (2016b), there is also a need for those working in the CCZ to make available detailed descriptions of equipment and methods to facilitate data standardization and statistically-rigorous regional comparisons. It is also important that the ISA-sponsored online atlas continues to be updated with new imagery (such as the images in this atlas), and that the morphospecies are properly identified with the help of taxonomists.

## Figures and Tables

**Figure 2a.**
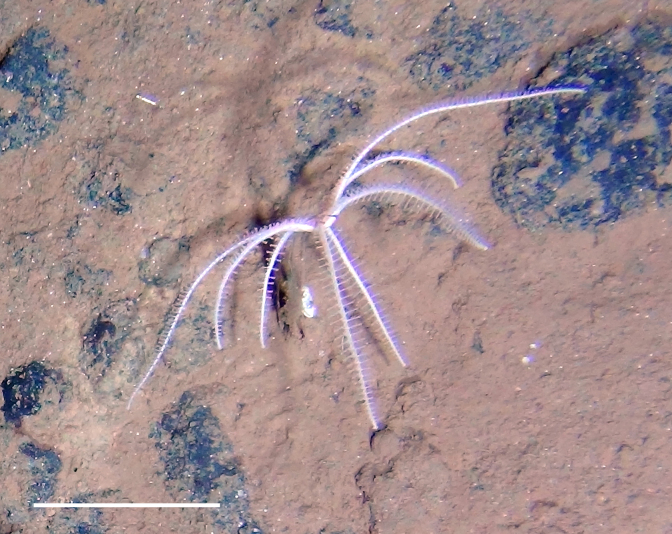
cf. *Freyella* morphospecies attached to dead sponge stalk. Scale bar is 10 cm. Image attribution: DJ Amon & CR Smith, University of Hawai’i.

**Figure 2b.**
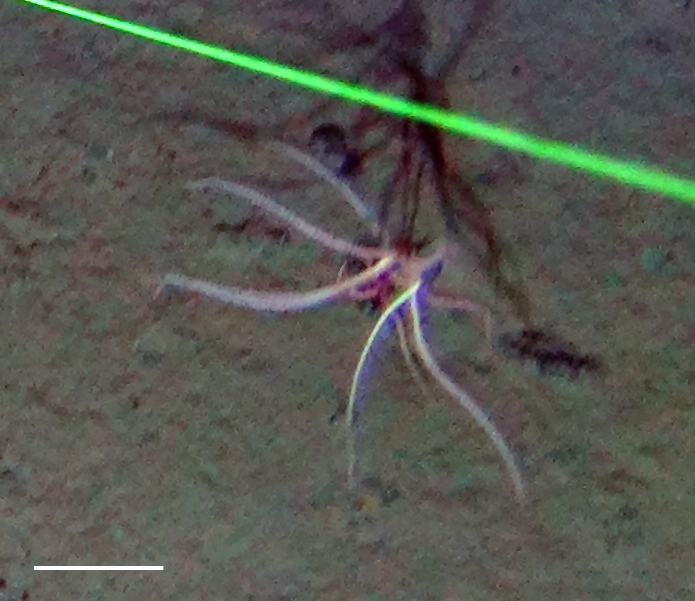
cf. *Freyella* morphospecies attached to dead sponge stalk. Scale bar is 10 cm. Image attribution: DJ Amon & CR Smith, University of Hawai’i.

**Figure 2c.**
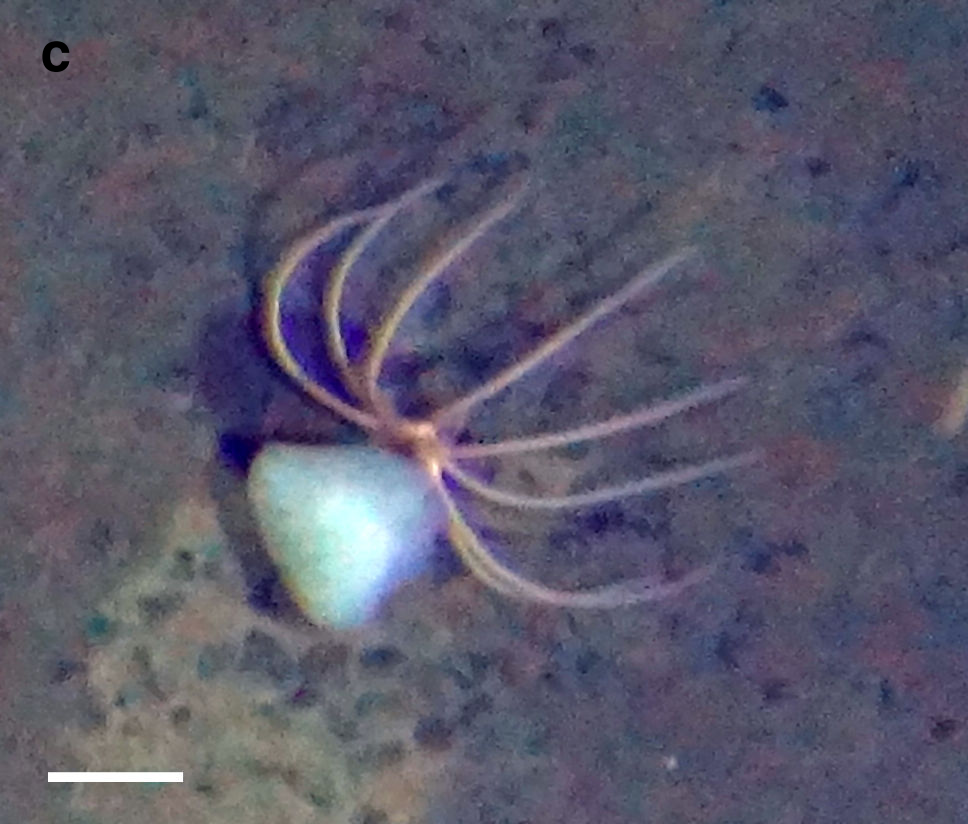
cf. *Freyella* morphospecies attached to a live sponge. Scale bar is 10 cm. Image attribution: DJ Amon & CR Smith, University of Hawai’i.

**Figure 2d.**
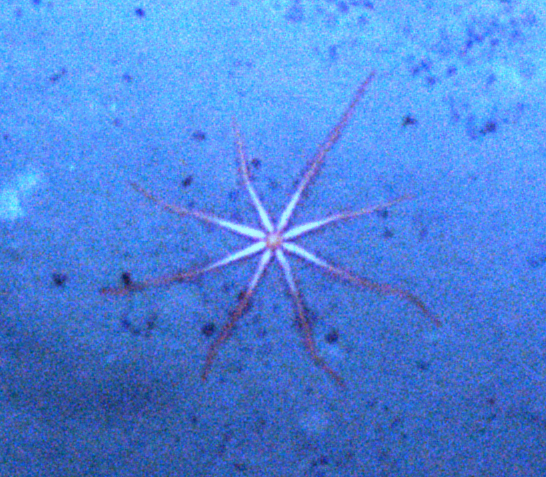
cf. *Freyella* morphospecies in situ on seafloor. Image attribution: Woods Hole Oceanographic Institution.

**Figure 3a.**
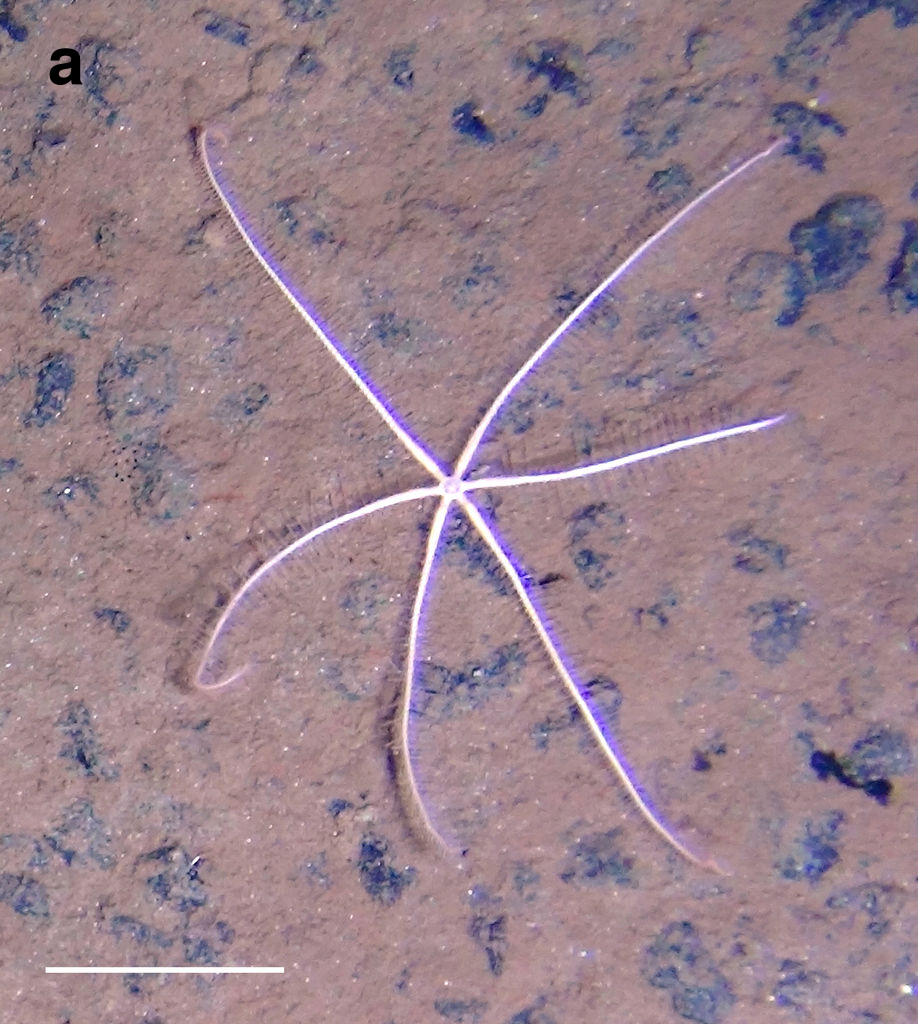
Freyastera cf. benthophila in situ on seafloor. Scale bar is 10 cm. Image attribution: DJ Amon & CR Smith, University of Hawai’i.

**Figure 3b.**
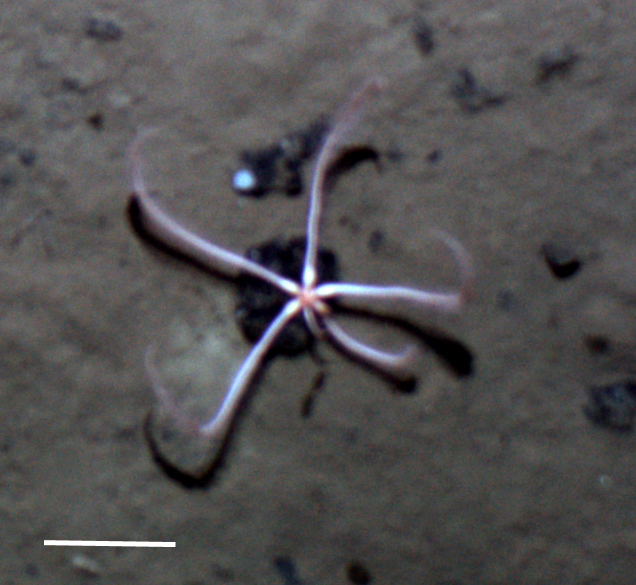
Freyastera cf. benthophila attached to a polymetallic nodule on seafloor. Scale bar is 10 cm. Image attribution: Woods Hole Oceanographic Institution.

**Figure 3c.**
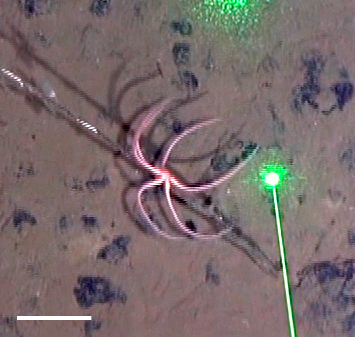
Freyastera cf. benthophila attached to dead stalk regenerating sixth arm. Scale bar is 10 cm. Image attribution: DJ Amon & CR Smith, University of Hawai’i.

**Figure 3d.**
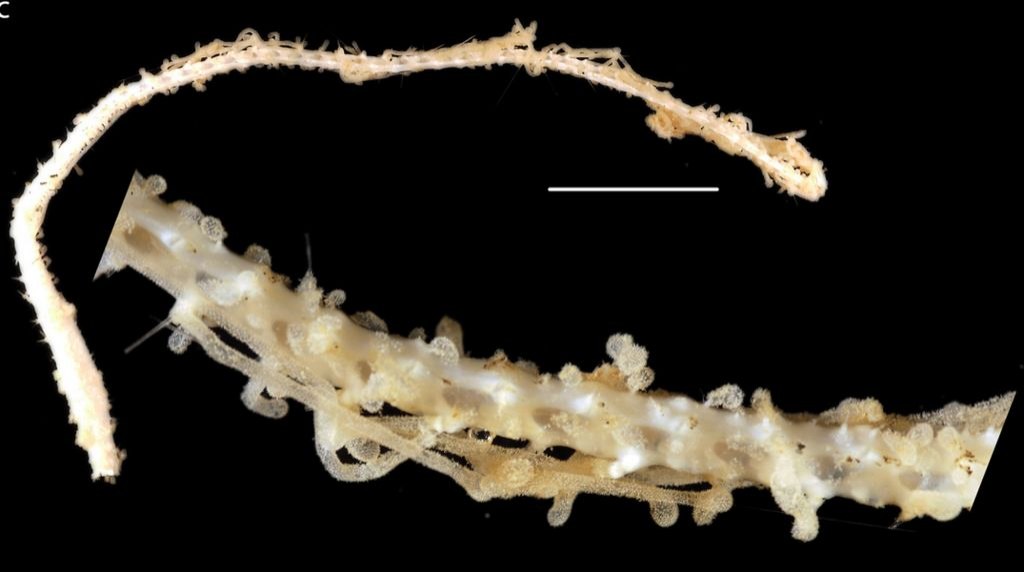
Tentacle of Freyastera cf. benthophila only partly recovered. Scale bar is 2 cm. Image attribution: AG Glover, TD Dahlgren & H Wiklund, Natural History Museum, London & Uni Research.

**Figure 4.**
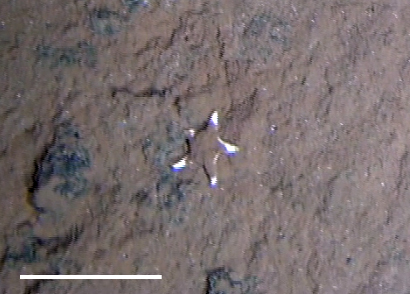
cf. Paxillosida morphospecies 1 in situ on seafloor in the UK-1 exploration contract area. Image corresponds with the data above. Scale bar is 10 cm. Image attribution: DJ Amon & CR Smith, University of Hawai’i.

**Figure 5.**
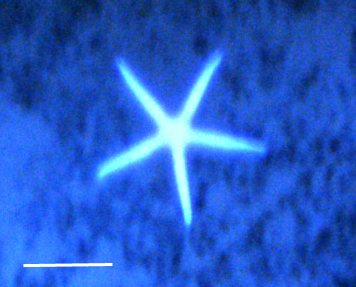
cf. Paxillosida morphospecies 2 in situ on seafloor in the UK-1 exploration contract area. Image corresponds with the data above. Scale bar is 10 cm. Image attribution: Woods Hole Oceanographic Institution.

**Figure 6.**
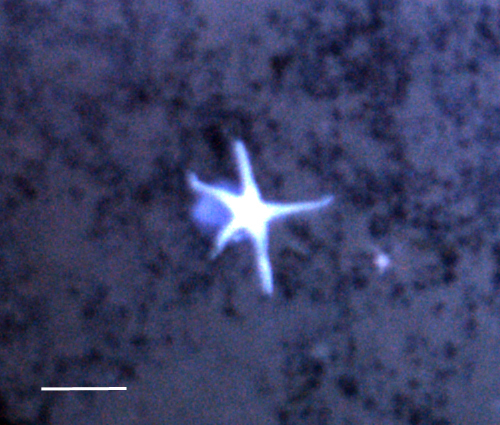
cf. Paxillosida morphospecies 3 observed in situ on sponge on seafloor in the UK-1 exploration contract area. Image corresponds with the data above. Scale bar is 10 cm. Image attribution: Woods Hole Oceanographic Institution.

**Figure 7a.**
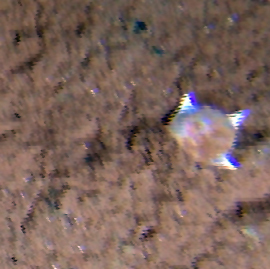
cf. Porcellanasteridae morphospecies in situ on seafloor. Image attribution: DJ Amon & CR Smith, University of Hawai’i.

**Figure 7b.**
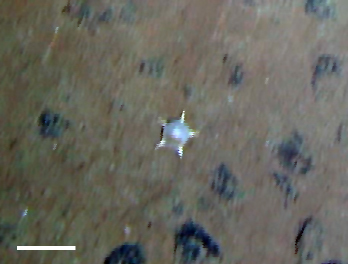
cf. Porcellanasteridae morphospecies in situ on seafloor. Scale bar is 10 cm. Image attribution: DJ Amon & CR Smith, University of Hawai’i.

**Figure 8a.**
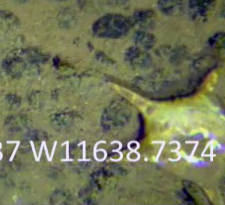
cf. *Porcellanaster* morphospecies in situ on seafloor. Image attribution: DJ Amon & CR Smith, University of Hawai’i.

**Figure 8b.**
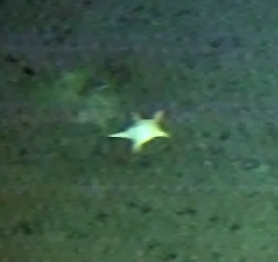
cf. *Porcellanaster* morphospecies in situ on seafloor. Image attribution: DJ Amon & CR Smith, University of Hawai’i.

**Figure 9.**
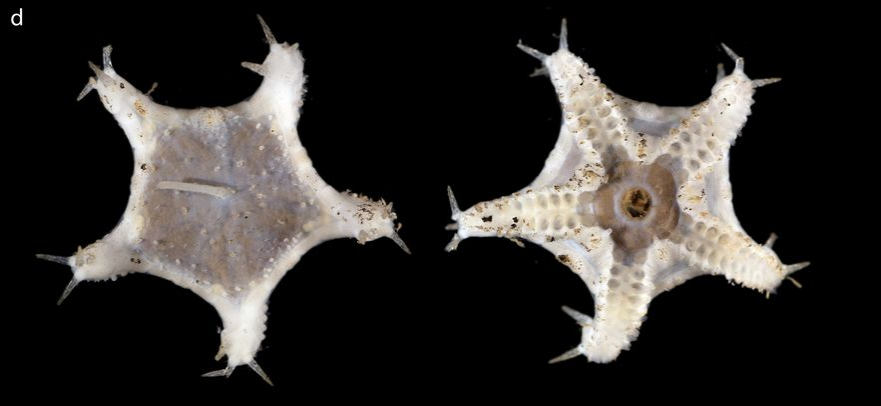
*Porcellanaster ceruleus* after collection from the UK-1 exploration contract area. Aboral view on left and oral view on right. Image corresponds with the data above. Image attribution: AG Glover, TD Dahlgren & H Wiklund, Natural History Museum, London & Uni Research.

**Figure 10a.**
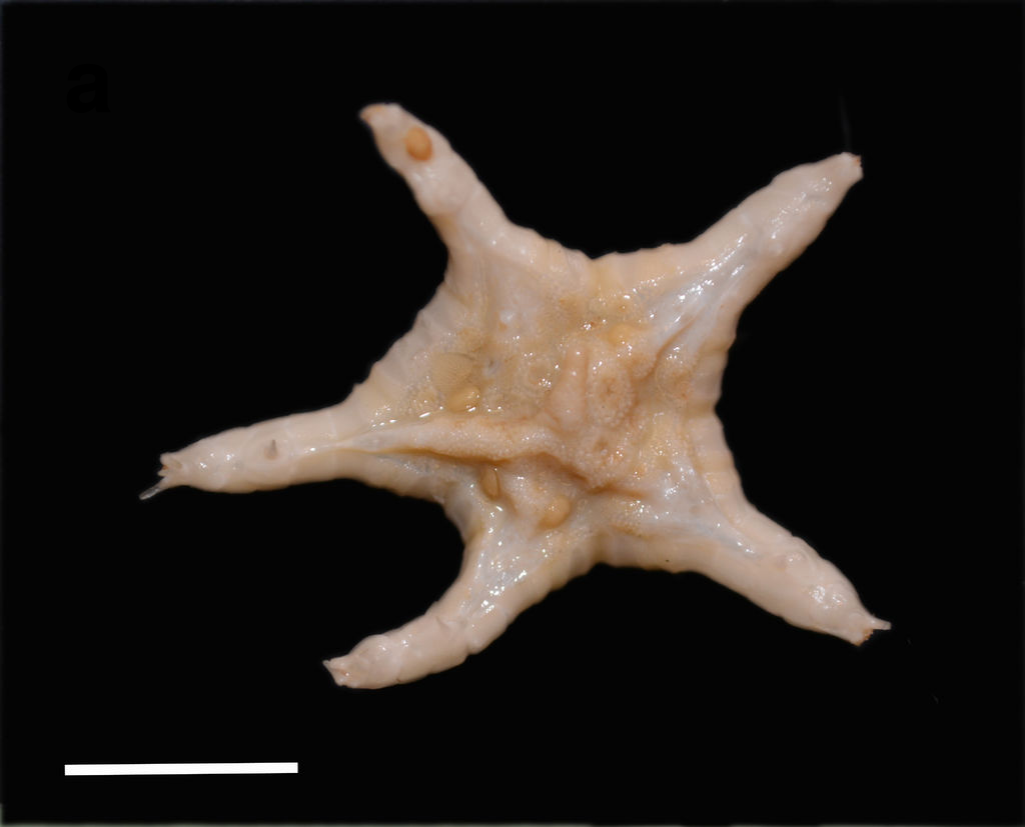
Aboral view of *Styracaster paucispinus* after collection. The pink dots on the specimen are parasites. Scale bar is 1 cm. Image attribution: DJ Amon and CR Smith.

**Figure 10b.**
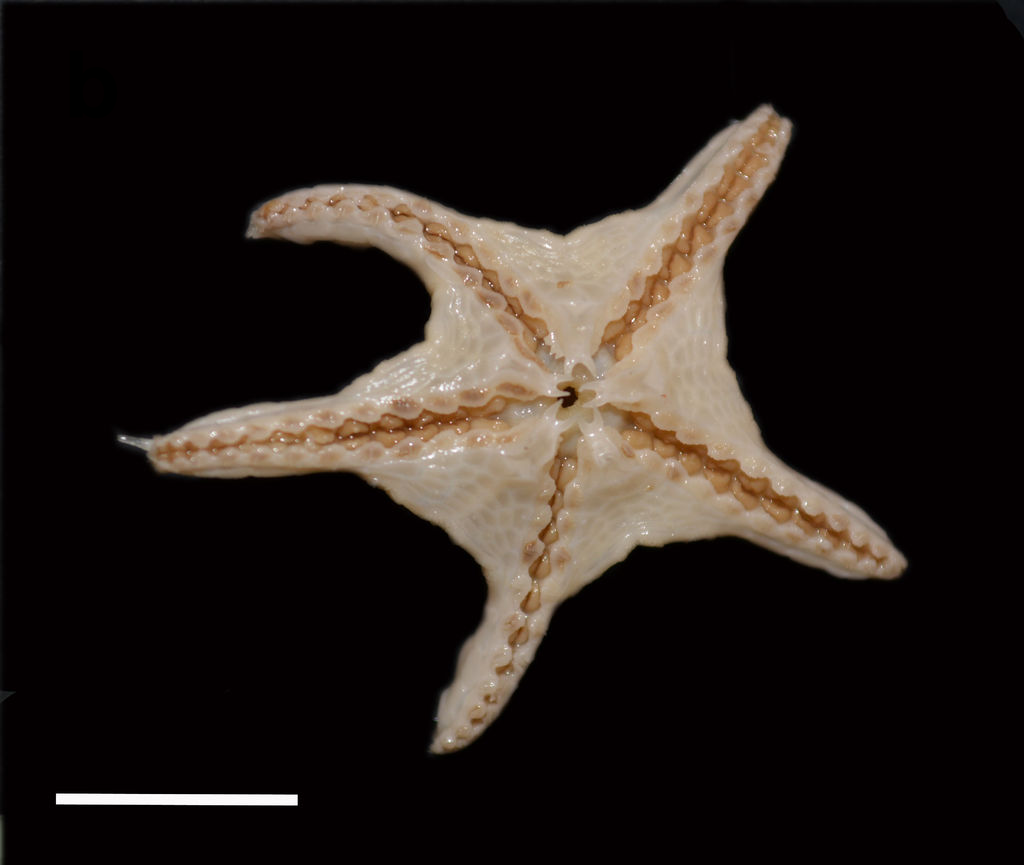
Oral view of *Styracaster paucispinus* after collection. Scale bar is 1 cm. Image attribution: DJ Amon and CR Smith.

**Figure 11a.**
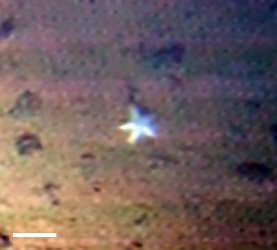
cf. Pterasteridae morphospecies 1 in situ on seafloor. Scale bar is 10 cm. Image attribution: DJ Amon & CR Smith, University of Hawai’i.

**Figure 11b.**
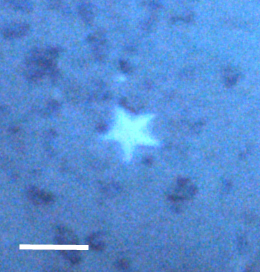
cf. Pterasteridae morphospecies 1 in situ on seafloor. Scale bar is 10 cm. Image attribution: Woods Hole Oceanographic Institution.

**Figure 12.**
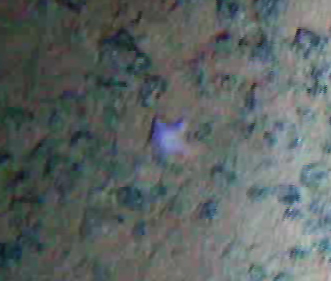
cf. Pterasteridae morphospecies 2 observed in the UK-1 exploration contract area. Image corresponds with the data above. Image attribution: DJ Amon & CR Smith, University of Hawai’i.

**Figure 13.**
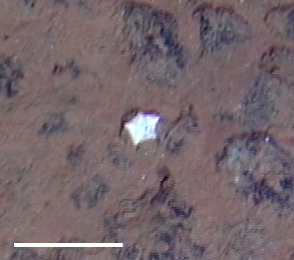
cf. *Hymenaster* morphospecies 1 in situ on seafloor in the UK-1 exploration contract area. Image corresponds with the data above. Scale bar is 10 cm. Image attribution: DJ Amon & CR Smith, University of Hawai’i.

**Figure 14a.**
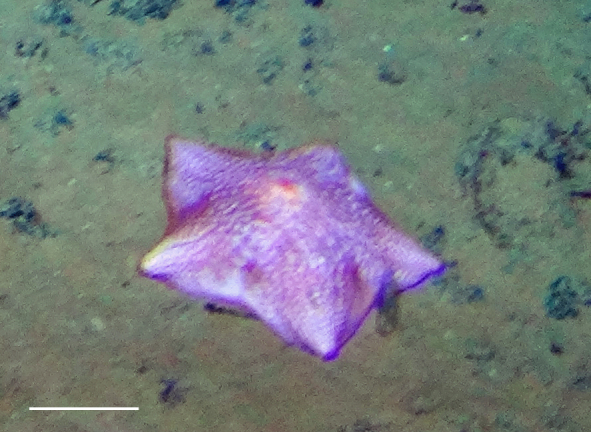
Side view of cf. *Hymenaster* morphospecies 2 in situ on seafloor. Scale bar is 10 cm. Image attribution: DJ Amon & CR Smith, University of Hawai’i.

**Figure 14b.**
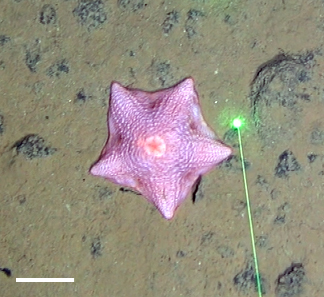
Aboral view of cf. *Hymenaster* morphospecies 2 in situ on seafloor. Scale bar is 10 cm. Image attribution: DJ Amon & CR Smith, University of Hawai’i.

**Figure 15a.**
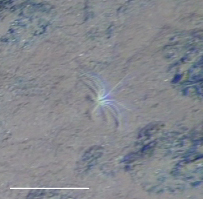
cf. Comatulida morphospecies 1 in situ on seafloor. Scale bar is 10 cm. Image attribution: DJ Amon & CR Smith, University of Hawai’i.

**Figure 15b.**
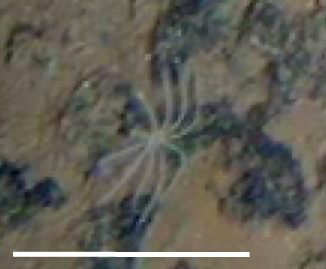
cf. Comatulida morphospecies 1 in situ on seafloor. Scale bar is 10 cm. Image attribution: DJ Amon & CR Smith, University of Hawai’i.

**Figure 16a.**
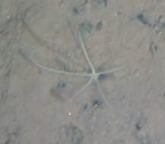
cf. Comatulida morphospecies 2 in situ on seafloor. Image attribution: DJ Amon & CR Smith, University of Hawai’i.

**Figure 16b.**
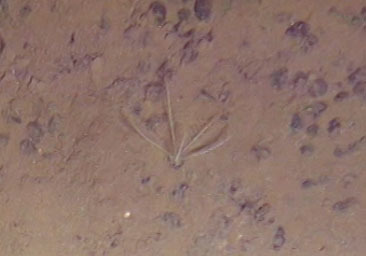
cf. Comatulida morphospecies 2 in situ on seafloor. Image attribution: DJ Amon & CR Smith, University of Hawai’i.

**Figure 17a.**
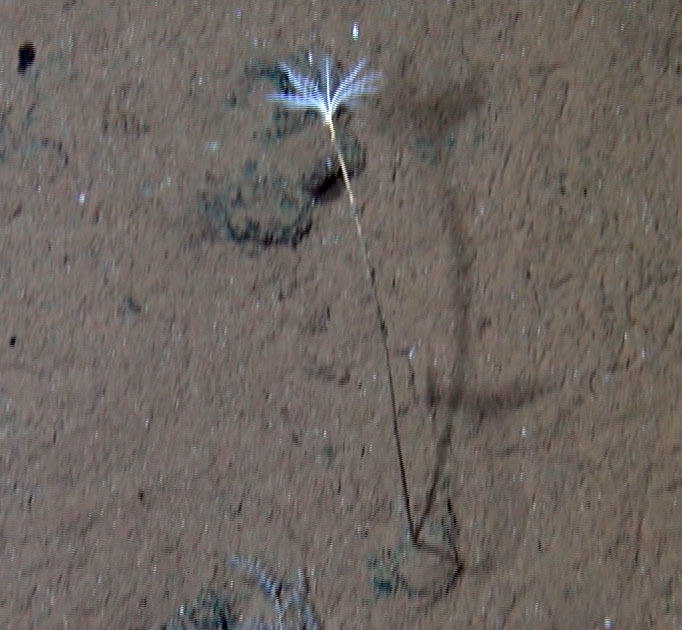
Bathycrinus cf. equatorialis in situ on seafloor. Image attribution: DJ Amon & CR Smith, University of Hawai’i.

**Figure 17b.**
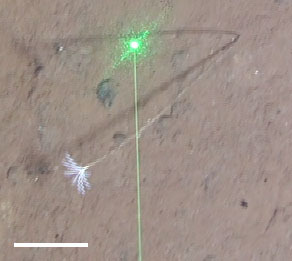
Bathycrinus cf. equatorialis in situ on seafloor. Scale bar is 10 cm. Image attribution: DJ Amon & CR Smith, University of Hawai’i.

**Figure 18.**
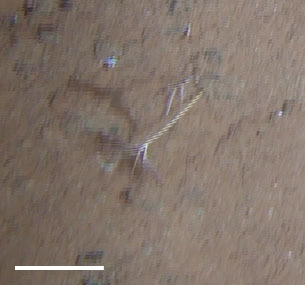
cf. Hyocrinidae morphospecies in situ on seafloor in the eastern CCZ. Image corresponds with the data above. Scale bar is 10 cm. Image attribution: DJ Amon & CR Smith, University of Hawai’i.

**Figure 19a.**
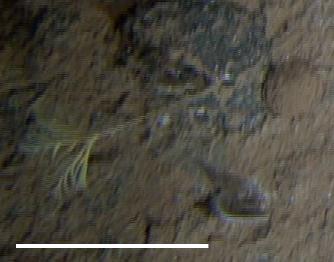
Hyocrinus cf. foelli in situ on seafloor. Scale bar is 10 cm. Image attribution: DJ Amon & CR Smith, University of Hawai’i.

**Figure 19b.**
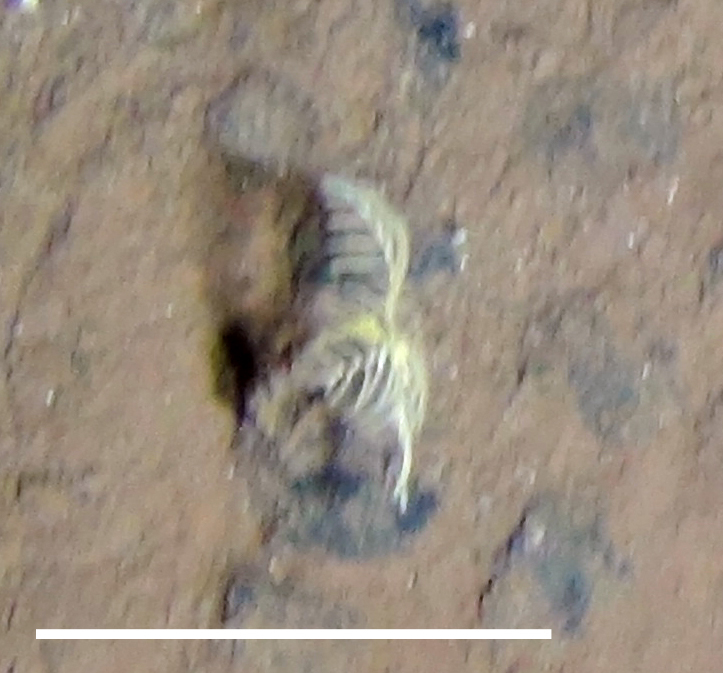
Hyocrinus cf. foelli in situ on seafloor. Scale bar is 10 cm. Image attribution: DJ Amon & CR Smith, University of Hawai’i.

**Figure 20a.**
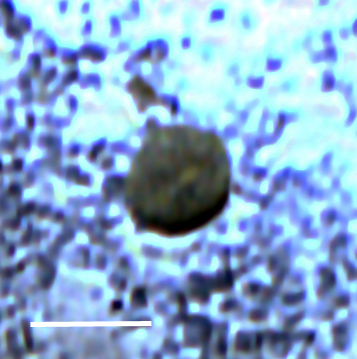
cf. Echinoidea morphospecies 1 in situ on seafloor. Scale bar is 10 cm. Image attribution: Woods Hole Oceanographic Institution.

**Figure 20b.**
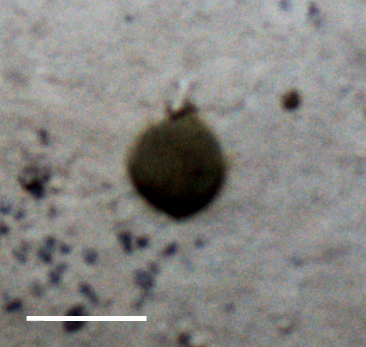
cf. Echinoidea morphospecies 1 in situ on seafloor. Scale bar is 10 cm. Image attribution: Woods Hole Oceanographic Institution.

**Figure 21.**
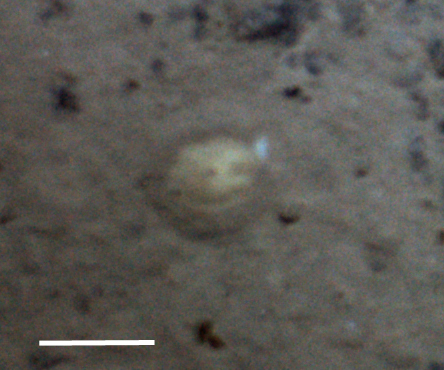
cf. Echinoidea morphospecies 2 in situ on seafloor in the UK-1 exploration contract area. Image corresponds with the data above. Scale bar is 10 cm. Image attribution: Woods Hole Oceanographic Institution.

**Figure 22a.**
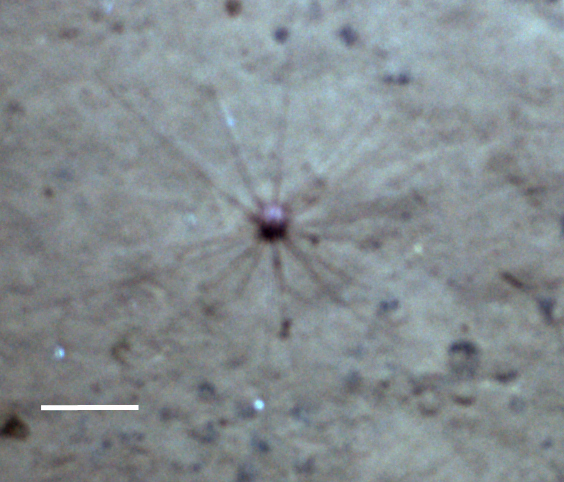
cf. Aspidodiadematidae morphospecies in situ on seafloor. Scale bar is 10 cm. Image attribution: Woods Hole Oceanographic Institution.

**Figure 22b.**
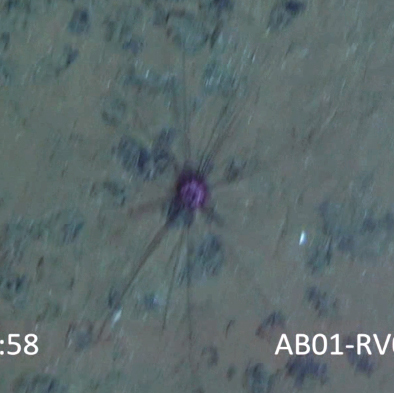
cf. Aspidodiadematidae morphospecies in situ on seafloor. Image attribution: DJ Amon & CR Smith, University of Hawai’i.

**Figure 23.**
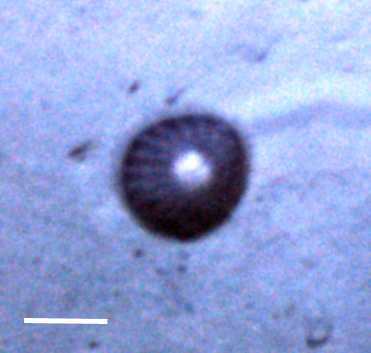
cf. Holasteroida morphospecies 1 in situ on seafloor in the UK-1 exploration contract area. Image corresponds with the data above. Scale bar is 10 cm. Image attribution: Woods Hole Oceanographic Institution.

**Figure 24a.**
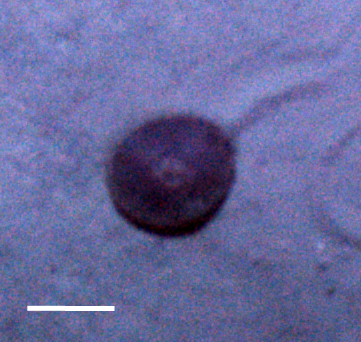
cf. Holasteroida morphospecies 2 in situ on seafloor. Scale bar is 10 cm. Image attribution: Woods Hole Oceanographic Institution.

**Figure 24b.**
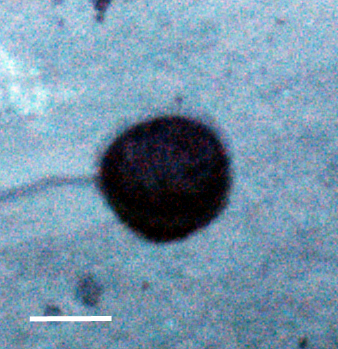
cf. Holasteroida morphospecies 2 in situ on seafloor. Scale bar is 10 cm. Image attribution: Woods Hole Oceanographic Institution.

**Figure 25.**
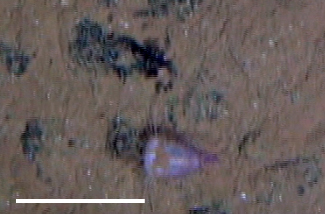
cf. *Cystocrepis* morphospecies in situ on seafloor in the UK-1 exploration contract area. Image corresponds with the data above. Scale bar is 10 cm. Image attribution: DJ Amon & CR Smith, University of Hawai’i.

**Figure 26a.**
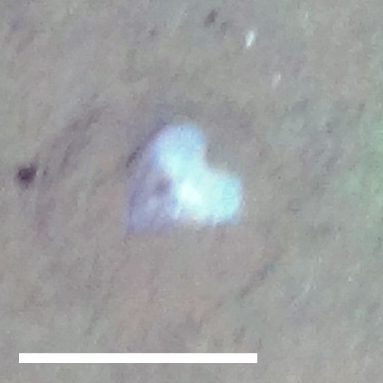
cf. *Echinocrepis* morphospecies in situ on seafloor. Scale bar is 10 cm. Image attribution: DJ Amon & CR Smith, University of Hawai’i.

**Figure 26b.**
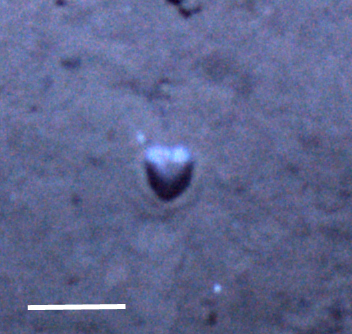
cf. *Echinocrepis* morphospecies in situ on seafloor. Scale bar is 10 cm. Image attribution: Woods Hole Oceanographic Institution.

**Figure 26c.**
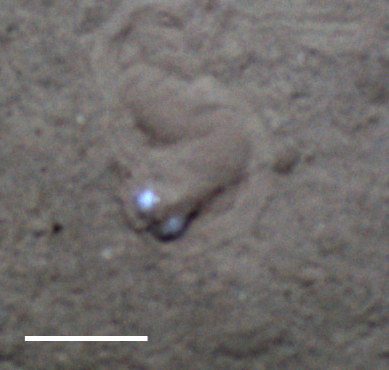
cf. *Echinocrepis* morphospecies in situ on seafloor. Scale bar is 10 cm. Image attribution: Woods Hole Oceanographic Institution.

**Figure 27.**
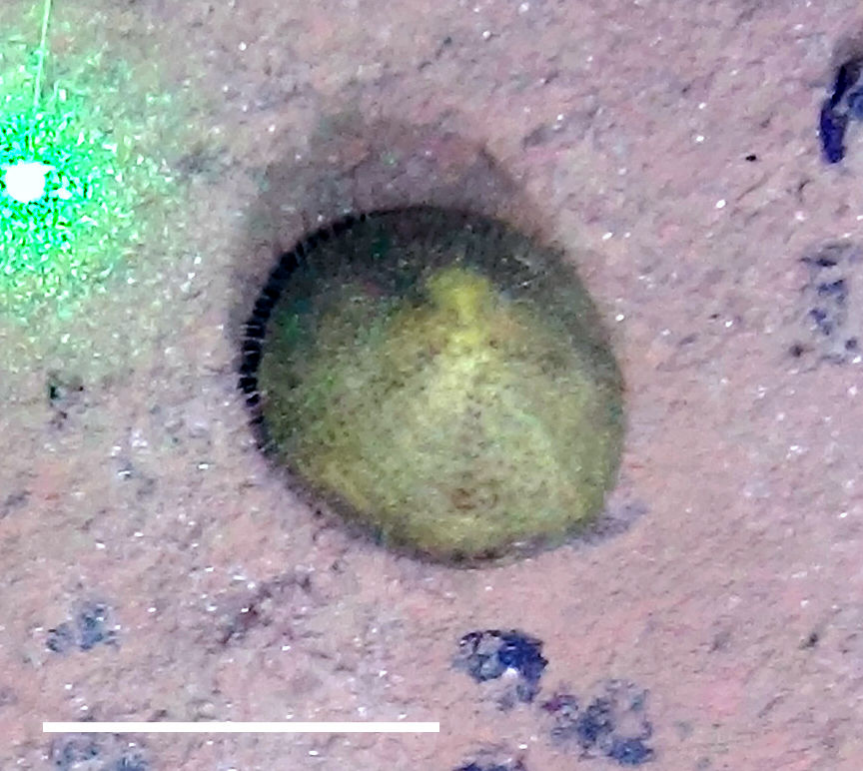
cf. Urechinidae morphospecies in situ on seafloor in the UK-1 exploration contract area. Image corresponds with the data above. Scale bar is 10 cm. Image attribution: DJ Amon & CR Smith, University of Hawai’i.

**Figure 28a.**
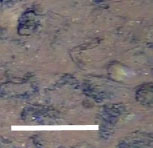
*Aceste ovata* in situ on seafloor. Image attribution: DJ Amon and CR Smith.

**Figure 28b.**
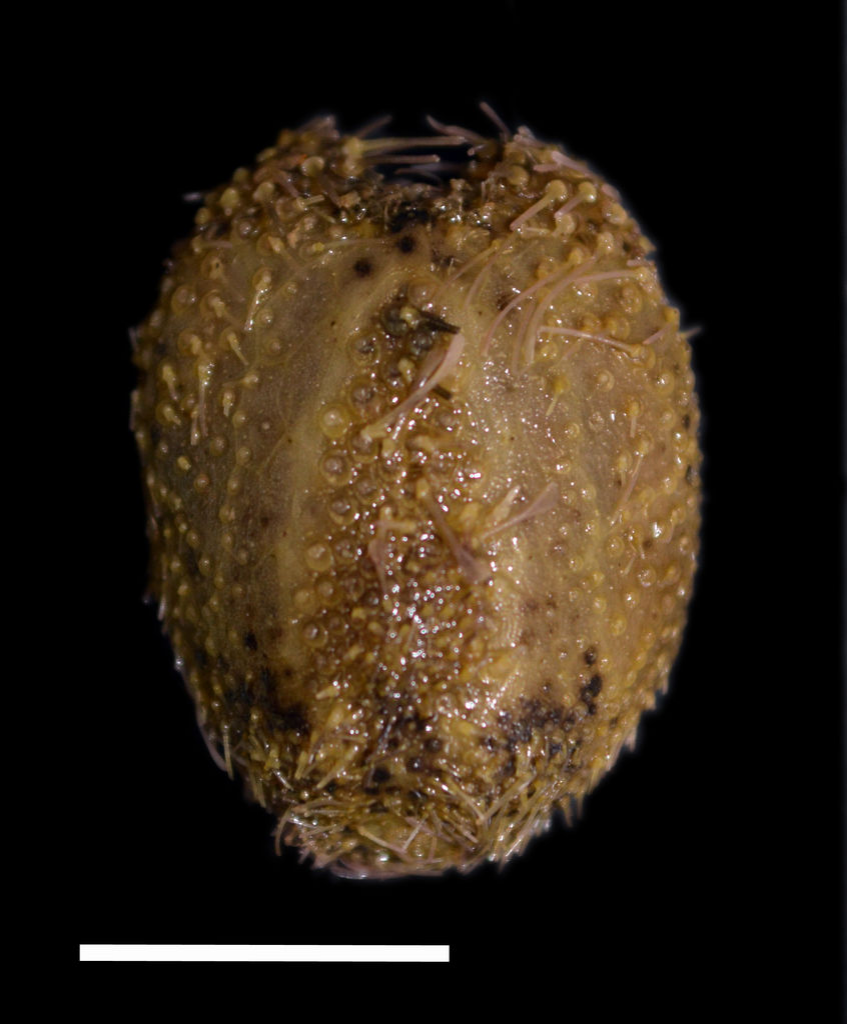
Aboral view of *Aceste ovata* after collection. Scale bar is 1 cm. Image attribution: DJ Amon and CR Smith.

**Figure 28c.**
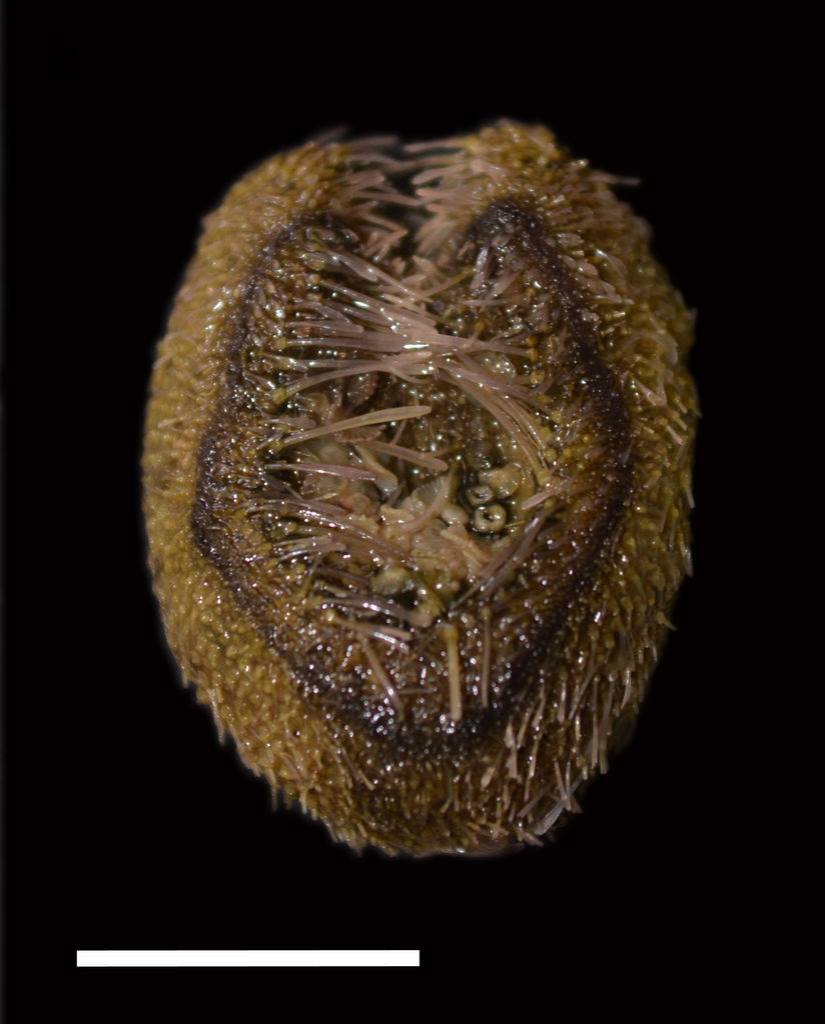
Oral view of *Aceste ovata* after collection. Scale bar is 1 cm. Image attribution: DJ Amon and CR Smith.

**Figure 29a.**
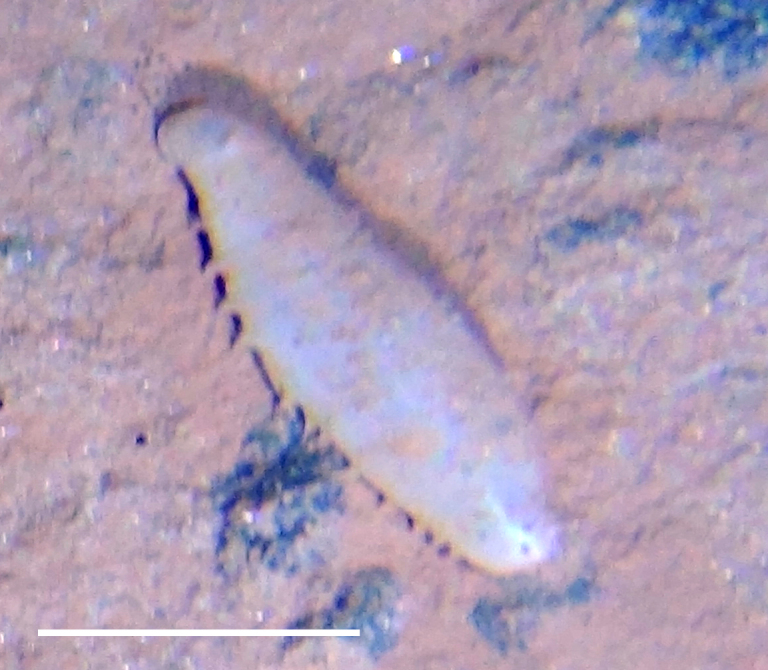
cf. *Mesothuria* morphospecies in situ on seafloor. Scale bar is 10 cm. Image attribution: DJ Amon & CR Smith, University of Hawai’i.

**Figure 29b.**
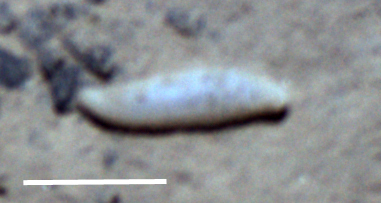
cf. *Mesothuria* morphospecies in situ on seafloor. Scale bar is 10 cm. Image attribution: Woods Hole Oceanographic Institution.

**Figure 30a.**
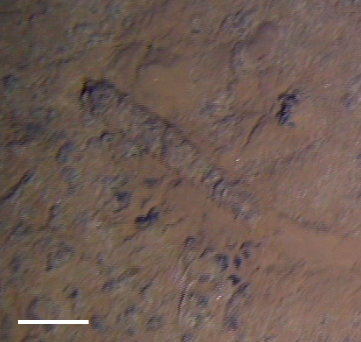
cf. Synallactidae morphospecies 1 in situ on seafloor. Scale bar is 10 cm. Image attribution: DJ Amon & CR Smith, University of Hawai’i.

**Figure 30b.**
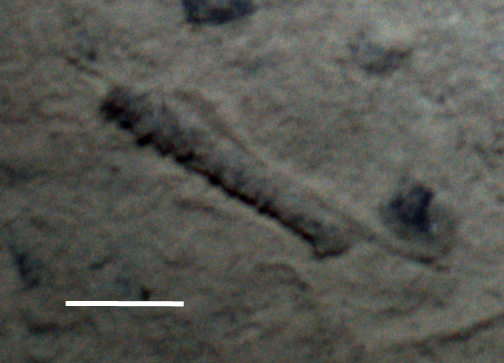
cf. Synallactidae morphospecies 1 in situ on seafloor. Scale bar is 10 cm. Image attribution: Woods Hole Oceanographic Institution.

**Figure 30c.**
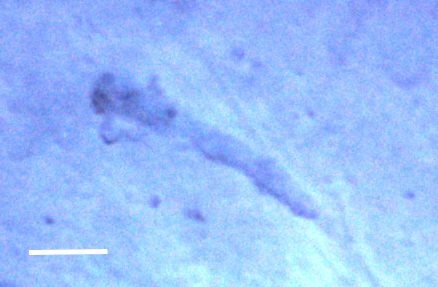
cf. Synallactidae morphospecies 1 in situ on seafloor. Scale bar is 10 cm. Image attribution: Woods Hole Oceanographic Institution.

**Figure 31.**
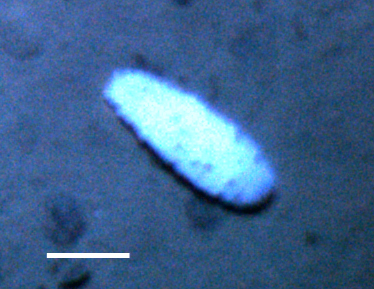
cf. Synallactidae morphospecies 2 in situ on seafloor in the UK-1 exploration contract area. Image corresponds with the data above. Scale bar is 10 cm. Image attribution: Woods Hole Oceanographic Institution.

**Figure 32a.**
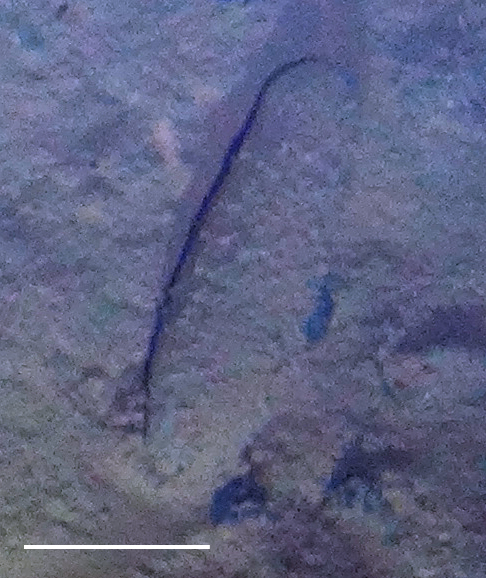
cf. *Molpadiodemas* morphospecies in situ on seafloor. Scale bar is 10 cm. Image attribution: DJ Amon & CR Smith, University of Hawai’i.

**Figure 32b.**
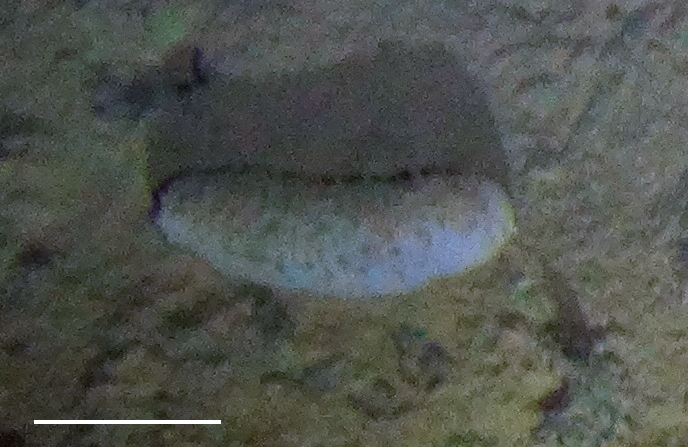
cf. *Molpadiodemas* morphospecies in situ on seafloor. Scale bar is 10 cm. Image attribution: DJ Amon & CR Smith, University of Hawai’i.

**Figure 32c.**
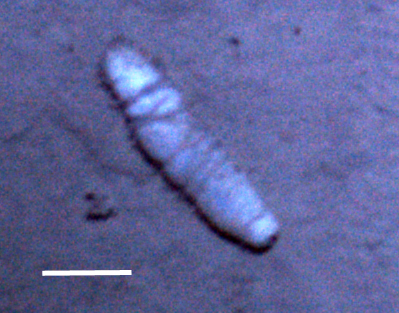
cf. *Molpadiodemas* morphospecies in situ on seafloor. Scale bar is 10 cm. Image attribution: Woods Hole Oceanographic Institution.

**Figure 32d.**
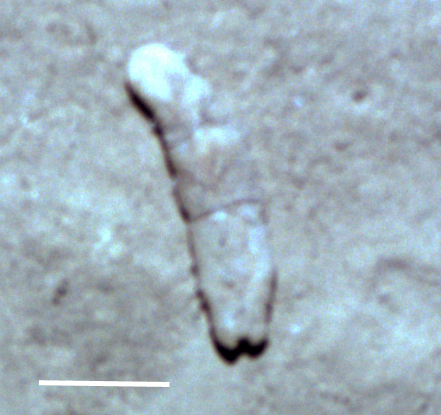
cf. *Molpadiodemas* morphospecies in situ on seafloor. Scale bar is 10 cm. Image attribution: Woods Hole Oceanographic Institution.

**Figure 33.**
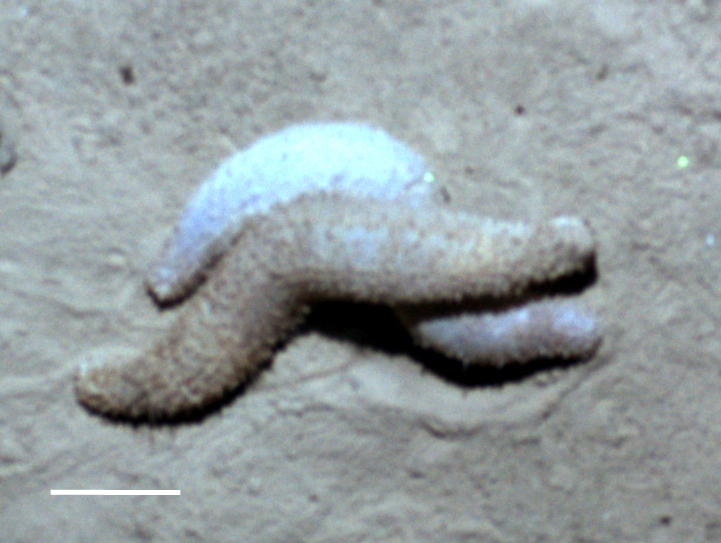
Two cf. *Paroriza* morphospecies specimens (likely male and female) in situ on seafloor in the UK-1 exploration contract area. Image corresponds with the data above. Scale bar is 10 cm. Image attribution: Woods Hole Oceanographic Institution.

**Figure 34a.**
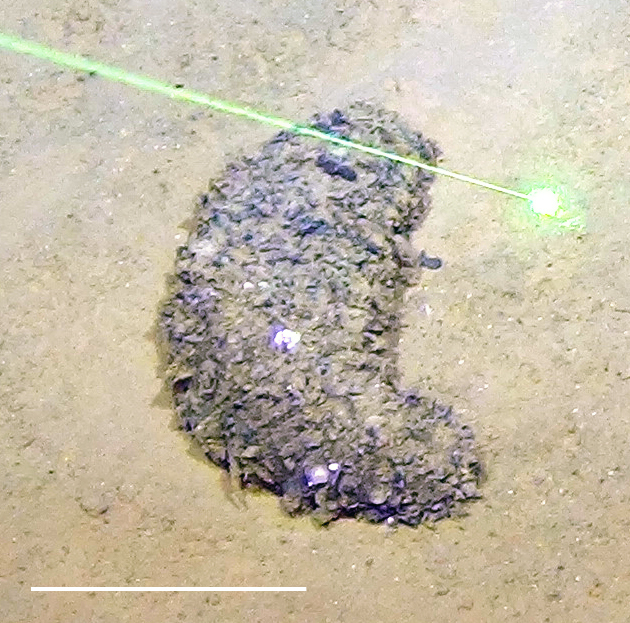
cf. *Pseudostichopus* morphospecies in situ on seafloor. Scale bar is 10 cm. Image attribution: DJ Amon & CR Smith, University of Hawai’i.

**Figure 34b.**
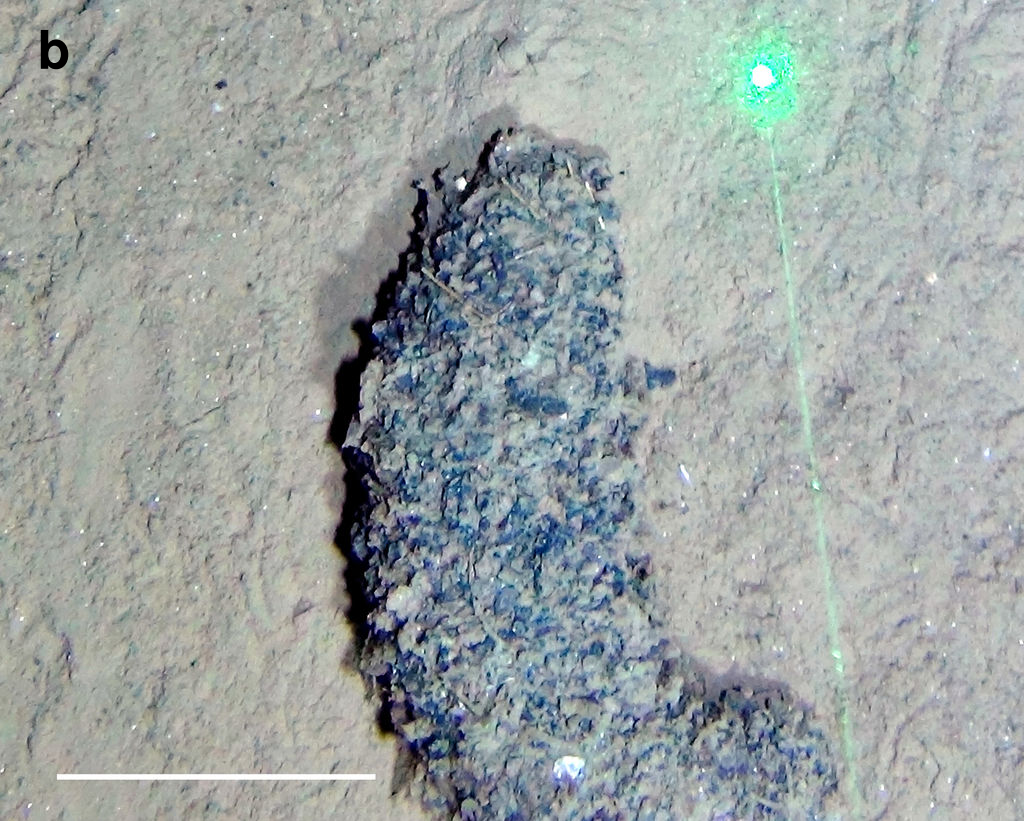
cf. *Pseudostichopus* morphospecies in situ on seafloor. Scale bar is 10 cm. Image attribution: DJ Amon & CR Smith, University of Hawai’i.

**Figure 34c.**
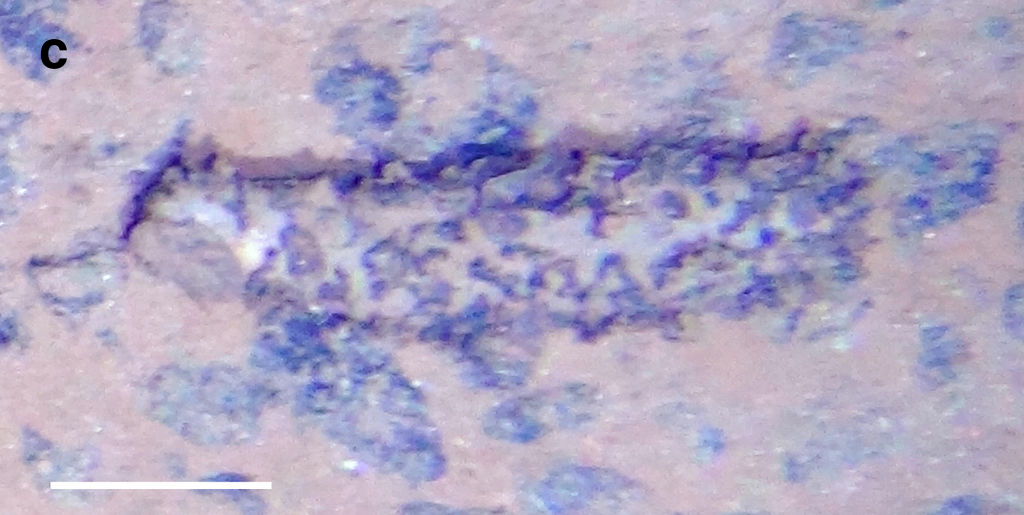
cf. *Pseudostichopus* morphospecies in situ on seafloor. Scale bar is 10 cm. Image attribution: DJ Amon & CR Smith, University of Hawai’i.

**Figure 35a.**
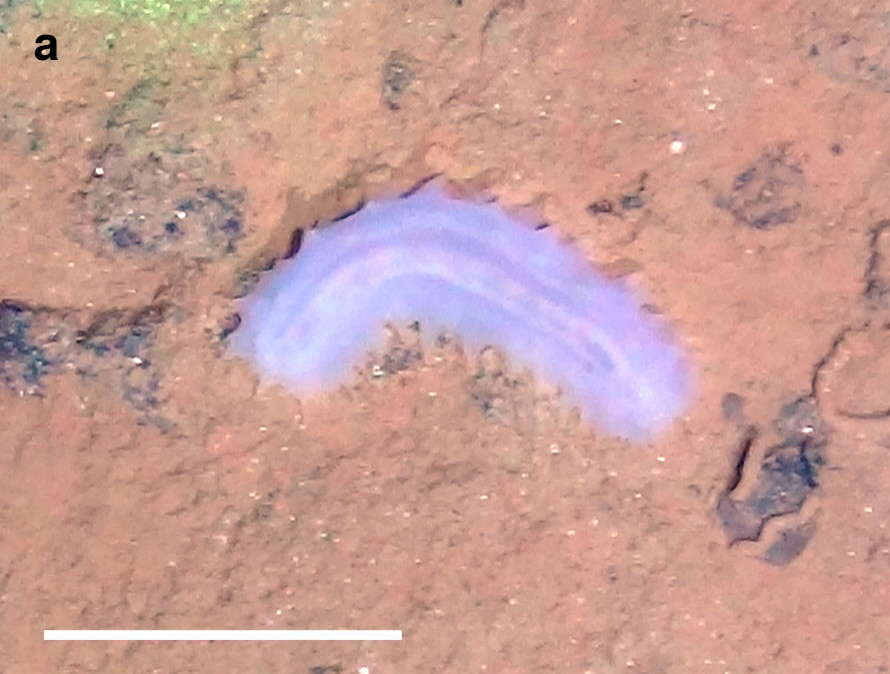
cf. *Synallactes* morphospecies 1 in situ on seafloor. Scale bar is 10 cm. Image attribution: DJ Amon & CR Smith, University of Hawai’i.

**Figure 35b.**
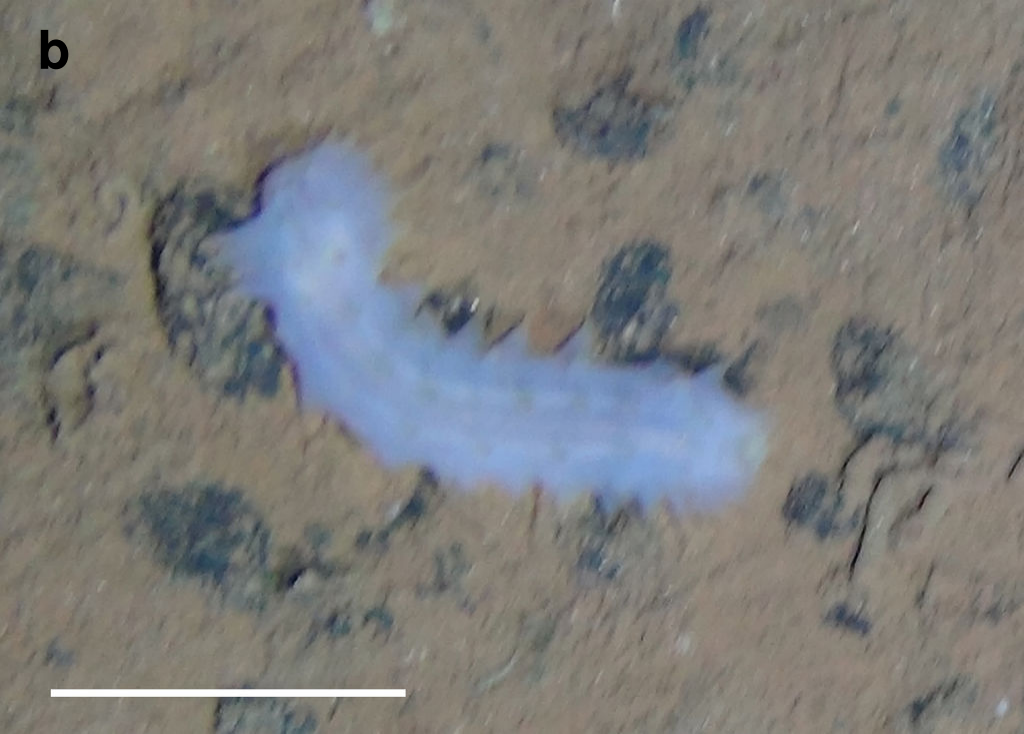
cf. *Synallactes* morphospecies 1 in situ on seafloor. Scale bar is 10 cm. Image attribution: DJ Amon & CR Smith, University of Hawai’i.

**Figure 35c.**
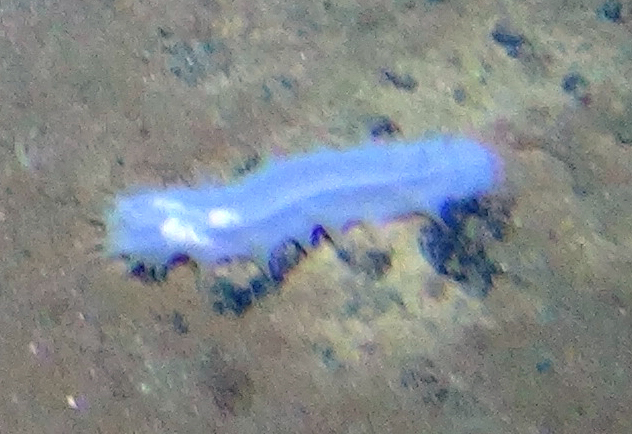
cf. *Synallactes* morphospecies 1 in situ on seafloor. Image attribution: DJ Amon & CR Smith, University of Hawai’i.

**Figure 36a.**
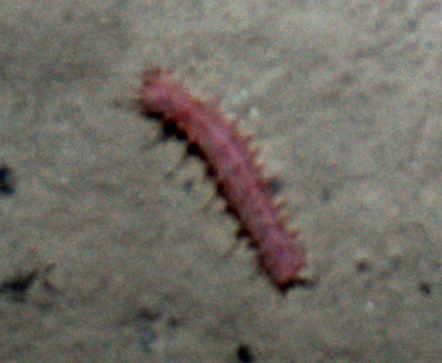
cf. *Synallactes* morphospecies 2 in situ on seafloor. Image attribution: Woods Hole Oceanographic Institution.

**Figure 36b.**
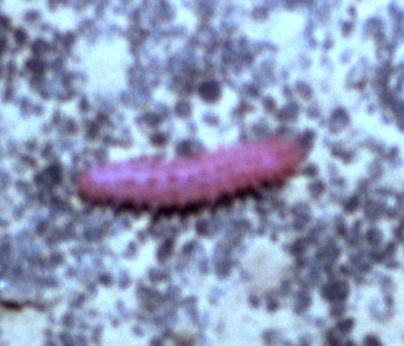
cf. *Synallactes* morphospecies 2 in situ on seafloor. Image attribution: Woods Hole Oceanographic Institution.

**Figure 37a.**
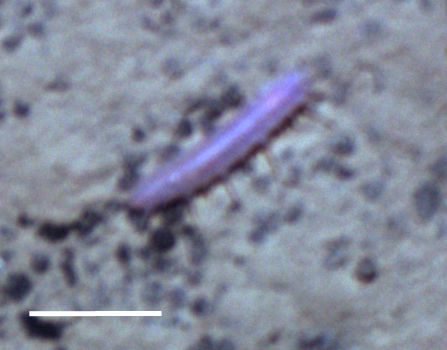
cf. *Synallactes* morphospecies 3 in situ on seafloor. Scale bar is 10 cm. Image attribution: Woods Hole Oceanographic Institution.

**Figure 37b.**
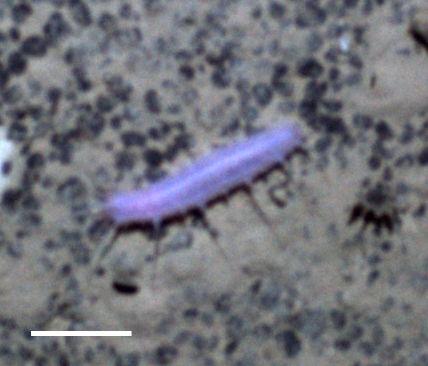
cf. *Synallactes* morphospecies 3 in situ on seafloor. Scale bar is 10 cm. Image attribution: Woods Hole Oceanographic Institution.

**Figure 38a.**
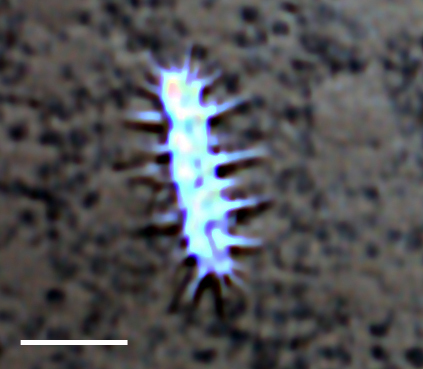
cf. Deimatidae morphospecies 1 in situ on seafloor. Scale bar is 10 cm. Image attribution: Woods Hole Oceanographic Institution.

**Figure 38b.**
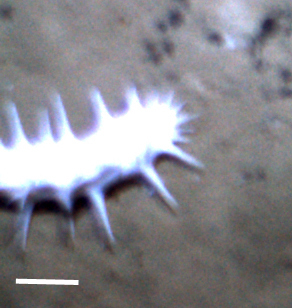
cf. Deimatidae morphospecies 1 in situ on seafloor. Scale bar is 5 cm. Image attribution: Woods Hole Oceanographic Institution.

**Figure 39.**
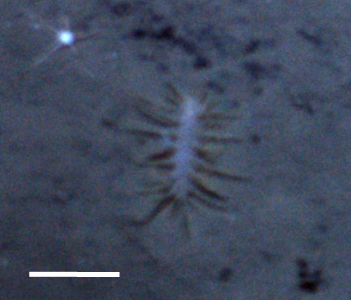
cf. Deimatidae morphospecies 2 observed in the UK-1 exploration contract area. Image corresponds with the data above. Scale bar is 10 cm. Image attribution: Woods Hole Oceanographic Institution.

**Figure 40a.**
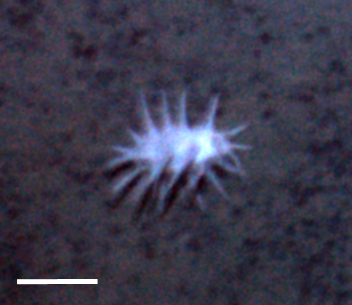
Deima cf. validum in situ on seafloor. Scale bar is 10 cm. Image attribution: Woods Hole Oceanographic Institution.

**Figure 40b.**
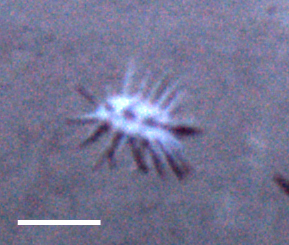
Deima cf. validum in situ on seafloor. Scale bar is 10 cm. Image attribution: Woods Hole Oceanographic Institution.

**Figure 41a.**
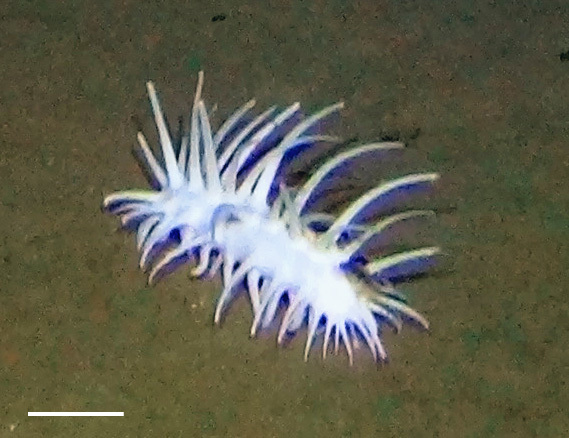
cf. *Oneirophanta* morphospecies in situ on seafloor. Scale bar is 10 cm. Image attribution: DJ Amon & CR Smith, University of Hawai’i.

**Figure 41b.**
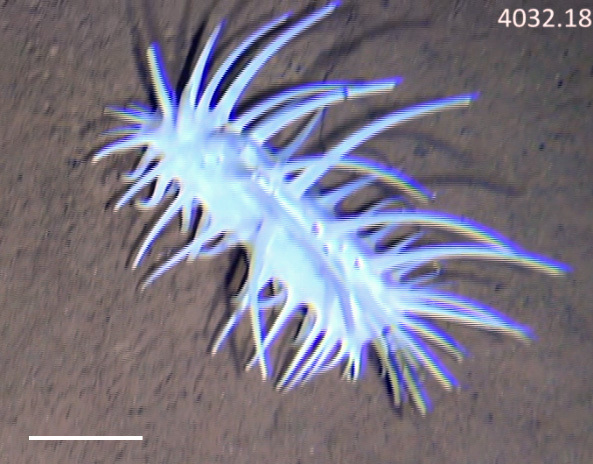
cf. *Oneirophanta* morphospecies in situ on seafloor. Scale bar is 10 cm. Image attribution: DJ Amon & CR Smith, University of Hawai’i.

**Figure 42.**
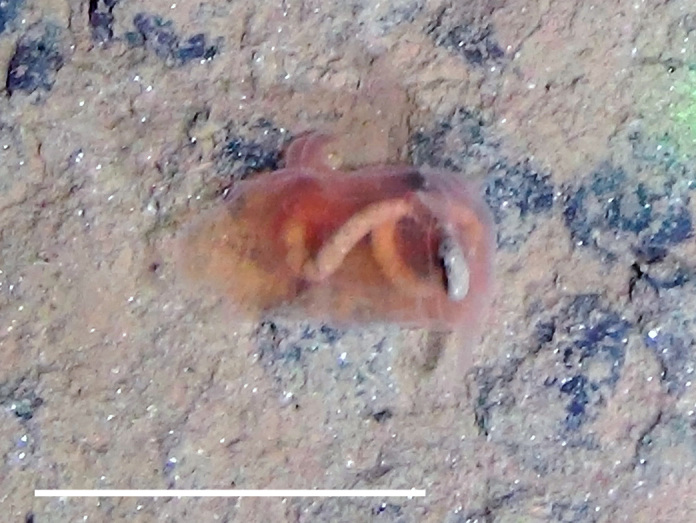
cf. Elpidiidae morphospecies 1 observed in the UK-1 exploration contract area. Image corresponds with the data above. Scale bar is 10 cm. Image attribution: DJ Amon & CR Smith, University of Hawai’i.

**Figure 43.**
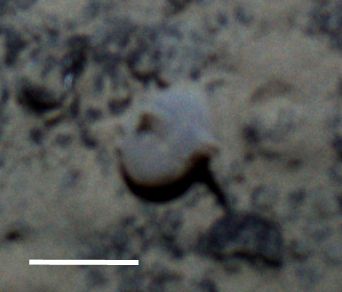
cf. Elpidiidae morphospecies 2 in situ on seafloor in the UK-1 exploration contract area. Image corresponds with the data above. Scale bar is 10 cm. Image attribution: Woods Hole Oceanographic Institution.

**Figure 44a.**
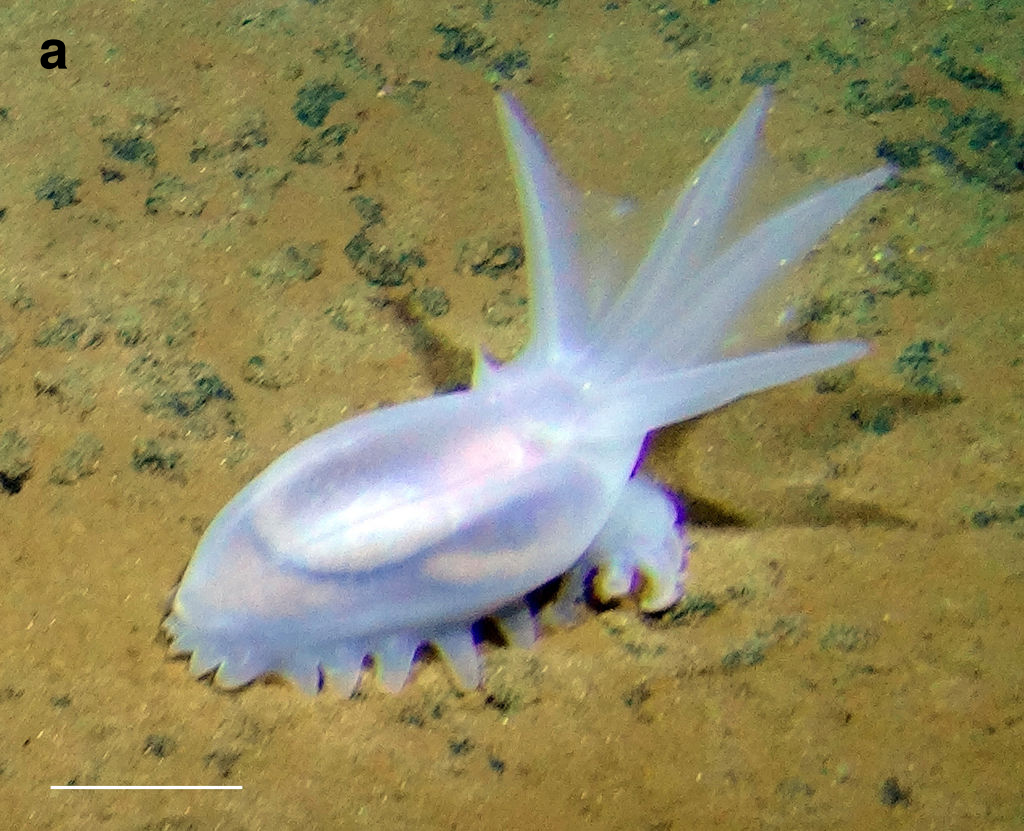
cf. *Amperima* morphospecies in situ on seafloor. Scale bar is 10 cm. Image attribution: DJ Amon & CR Smith, University of Hawai’i.

**Figure 44b.**
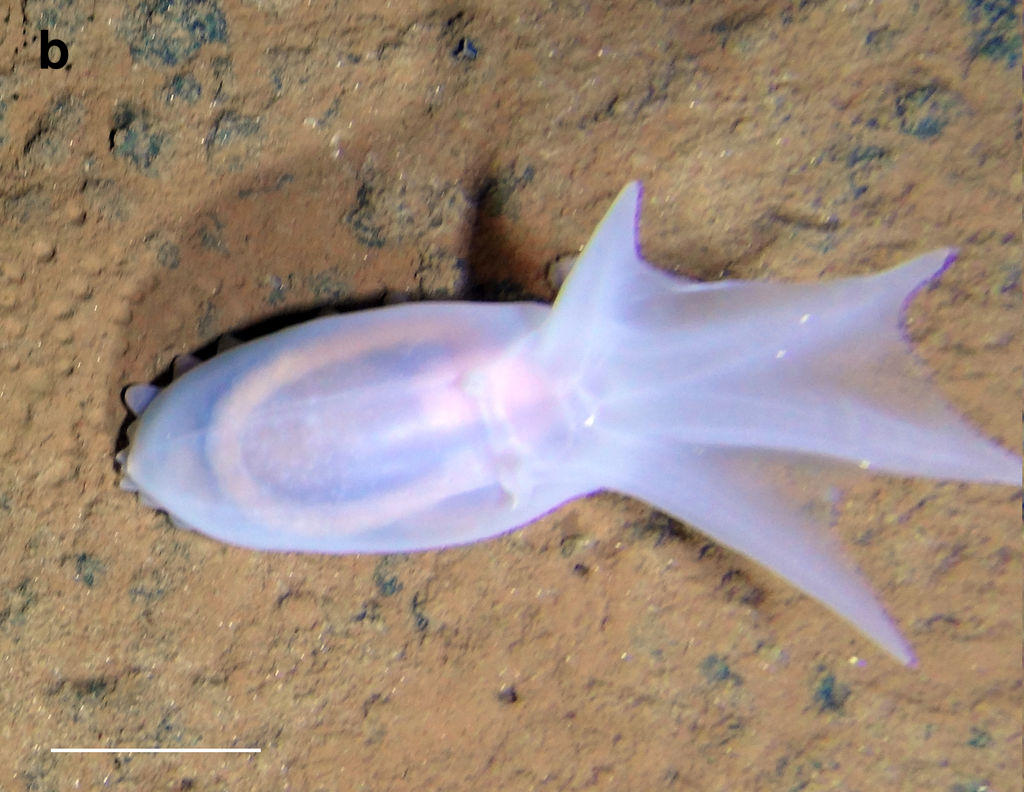
cf. *Amperima* morphospecies in situ on seafloor. Scale bar is 10 cm. Image attribution: DJ Amon & CR Smith, University of Hawai’i.

**Figure 45.**
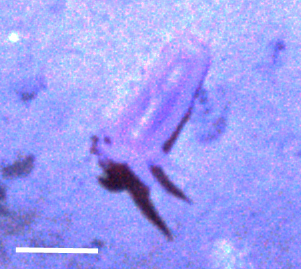
cf. *Peniagone* morphospecies 1 in situ on seafloor in the UK-1 exploration contract area. Image corresponds with the data above. Scale bar is 10 cm. Image attribution: Woods Hole Oceanographic Institution.

**Figure 46a.**
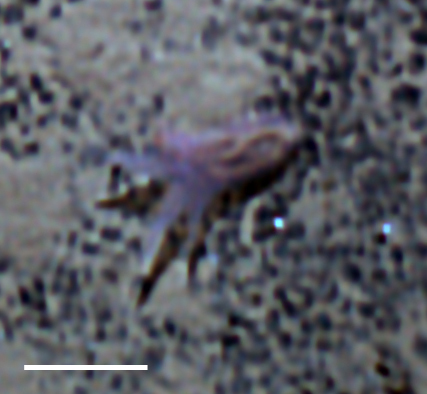
cf. *Peniagone* morphospecies 2 in situ on seafloor. Scale bar is 10 cm. Image attribution: Woods Hole Oceanographic Institution.

**Figure 46b.**
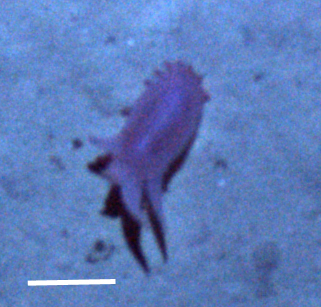
cf. *Peniagone* morphospecies 2 in situ on seafloor. Scale bar is 10 cm. Image attribution: Woods Hole Oceanographic Institution.

**Figure 47.**
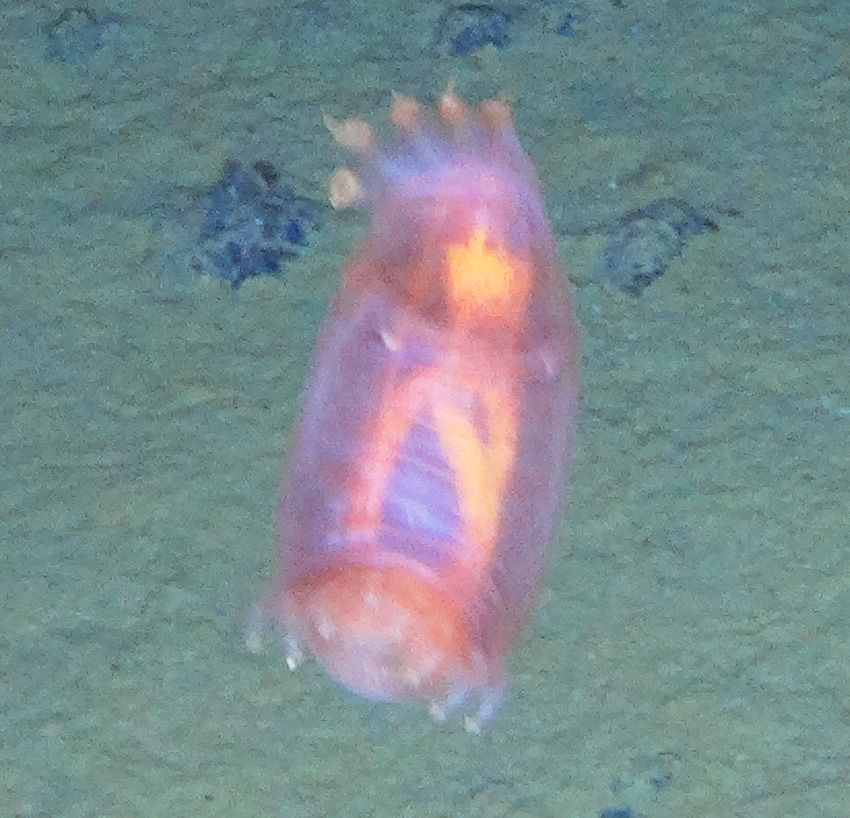
Peniagone cf. leander in situ on seafloor in the UK-1 exploration contract area. Image corresponds with the data above. Image attribution: DJ Amon & CR Smith, University of Hawai’i.

**Figure 48.**
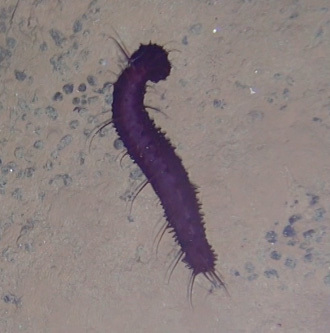
cf. Laetmogonidae morphospecies in situ on seafloor in the UK-1 exploration contract area. Image corresponds with the data above. Image attribution: DJ Amon & CR Smith, University of Hawai’i.

**Figure 49a.**
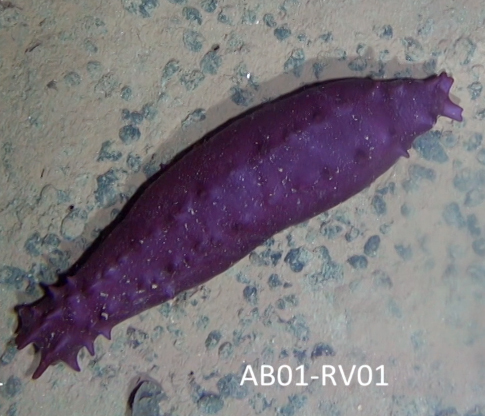
Psychronaetes cf. hanseni in situ on seafloor. Image attribution: DJ Amon & CR Smith, University of Hawai’i.

**Figure 49b.**
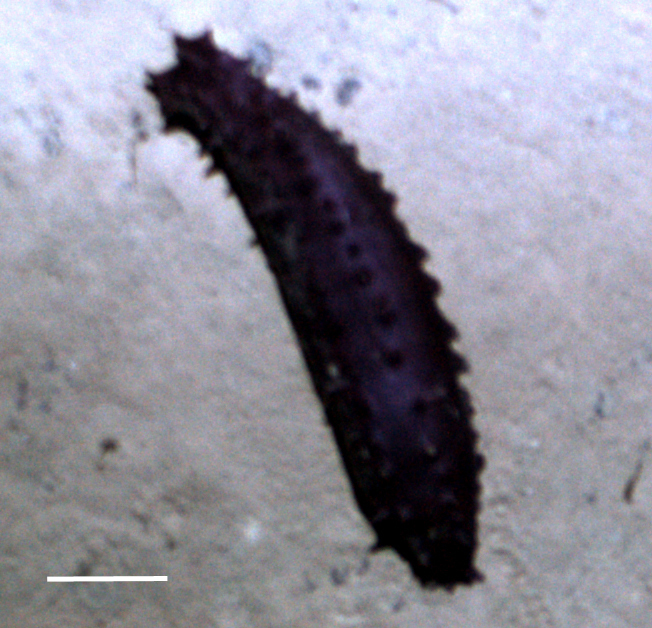
Psychronaetes cf. hanseni in situ on seafloor. Scale bar is 10 cm. Image attribution: Woods Hole Oceanographic Institution.

**Figure 50.**
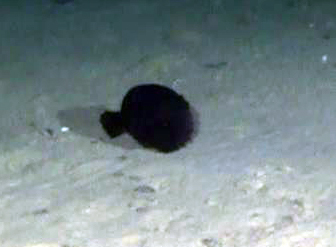
cf. *Enypniastes* morphospecies observed in situ on seafloor the UK-1 exploration contract area. Image corresponds with the data above. Image attribution: A Leitner and J Drazen, University of Hawai'i.

**Figure 51a.**
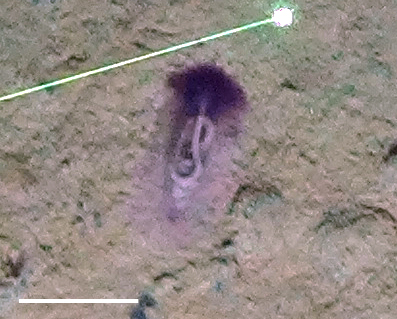
cf. Psychropotidae morphospecies (young specimen) in situ on seafloor. Scale bar is 10 cm. Image attribution: DJ Amon & CR Smith, University of Hawai’i.

**Figure 51b.**
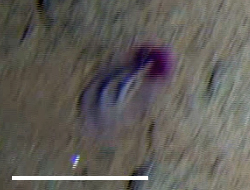
cf. Psychropotidae morphospecies (young specimen) in situ on seafloor. Scale bar is 10 cm. Image attribution: DJ Amon & CR Smith, University of Hawai’i.

**Figure 52a.**
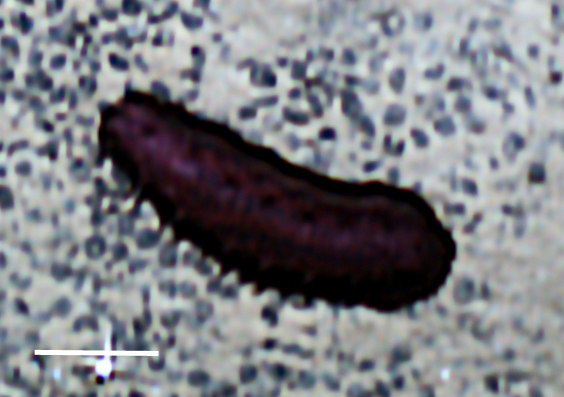
cf. *Benthodytes* morphospecies 1 in situ on seafloor. Scale bar is 10 cm. Image attribution: Woods Hole Oceanographic Institution.

**Figure 52b.**
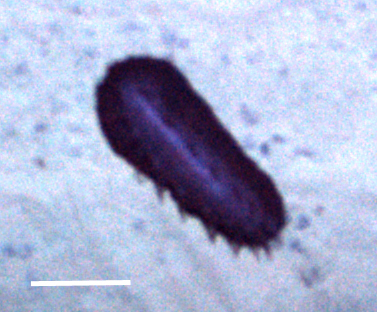
cf. *Benthodytes* morphospecies 1 in situ on seafloor. Scale bar is 10 cm. Image attribution: Woods Hole Oceanographic Institution.

**Figure 53.**
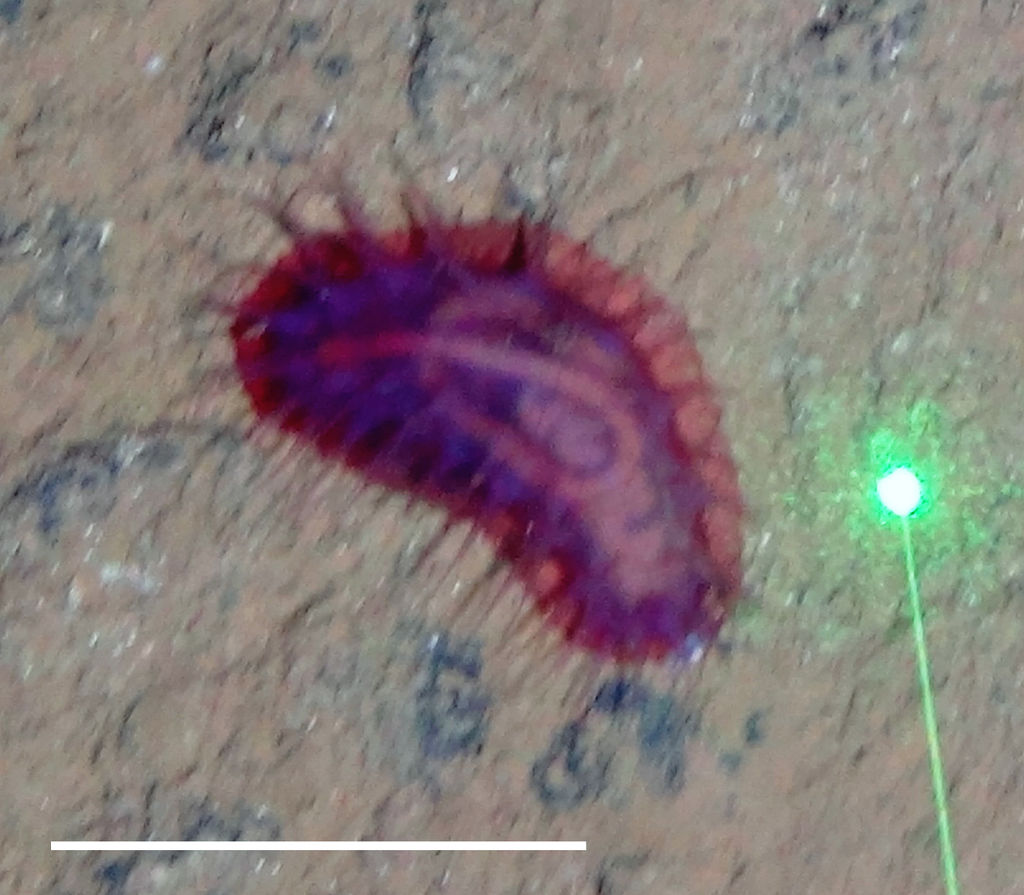
cf. *Benthodytes* morphospecies 2 (young specimen) in situ on seafloor in the UK-1 exploration contract area. Image corresponds with the data above. Scale bar is 10 cm. Image attribution: DJ Amon & CR Smith, University of Hawai’i.

**Figure 54a.**
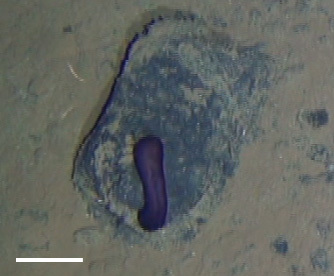
Benthodytes cf. incerta in situ on seafloor. Scale bar is 10 cm. Image attribution: DJ Amon & CR Smith, University of Hawai’i.

**Figure 54b.**
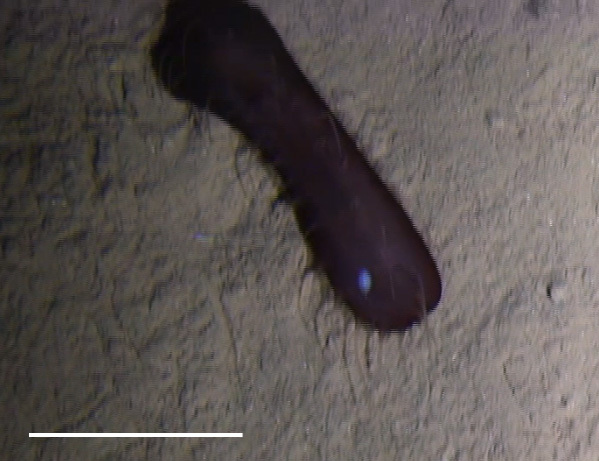
Benthodytes cf. incerta in situ on seafloor. Scale bar is 10 cm. Image attribution: DJ Amon & CR Smith, University of Hawai’i.

**Figure 54c.**
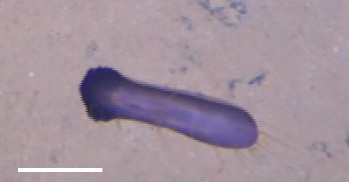
Benthodytes cf. incerta in situ on seafloor. Scale bar is 10 cm. Image attribution: DJ Amon & CR Smith, University of Hawai’i.

**Figure 55a.**
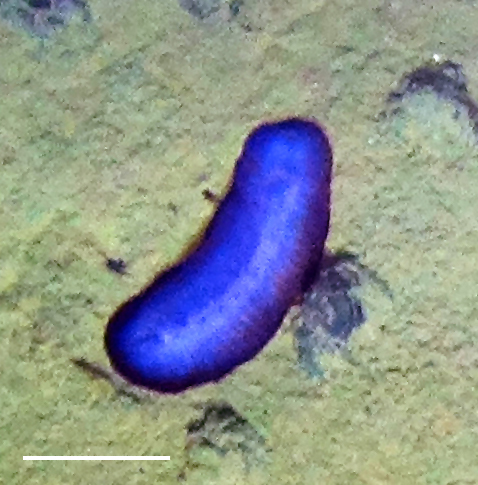
Benthodytes cf. typica in situ on seafloor. Scale bar is 10 cm. Image attribution: DJ Amon & CR Smith, University of Hawai’i.

**Figure 55b.**
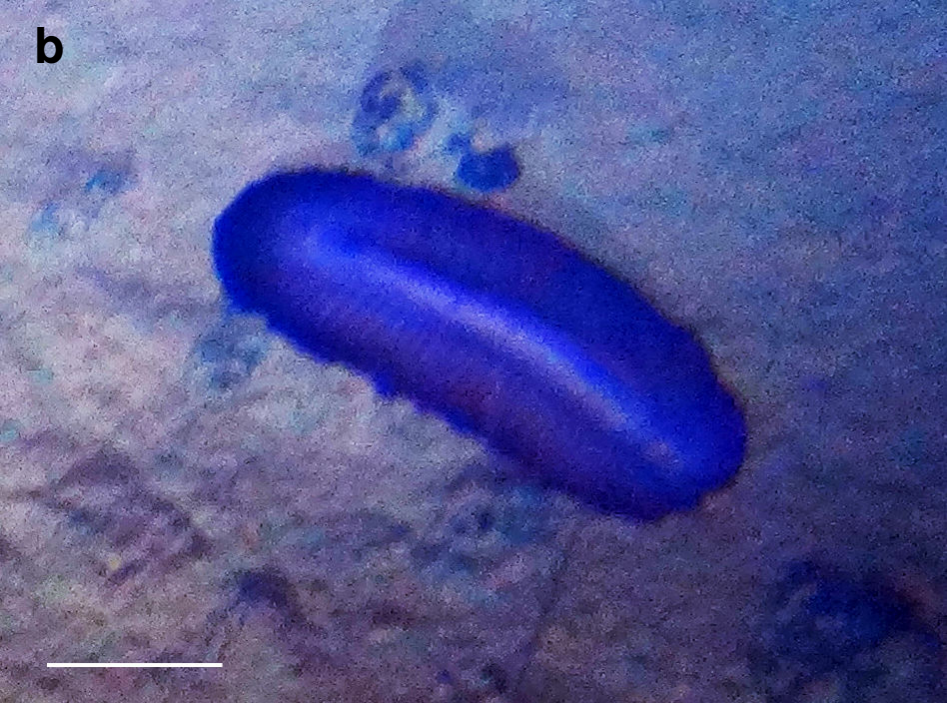
Benthodytes cf. typica in situ on seafloor. Scale bar is 10 cm. Image attribution: DJ Amon & CR Smith, University of Hawai’i.

**Figure 55c.**
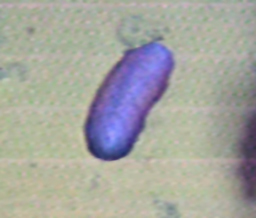
Benthodytes cf. typica in situ on seafloor. Image attribution: DJ Amon & CR Smith, University of Hawai’i.

**Figure 55d.**
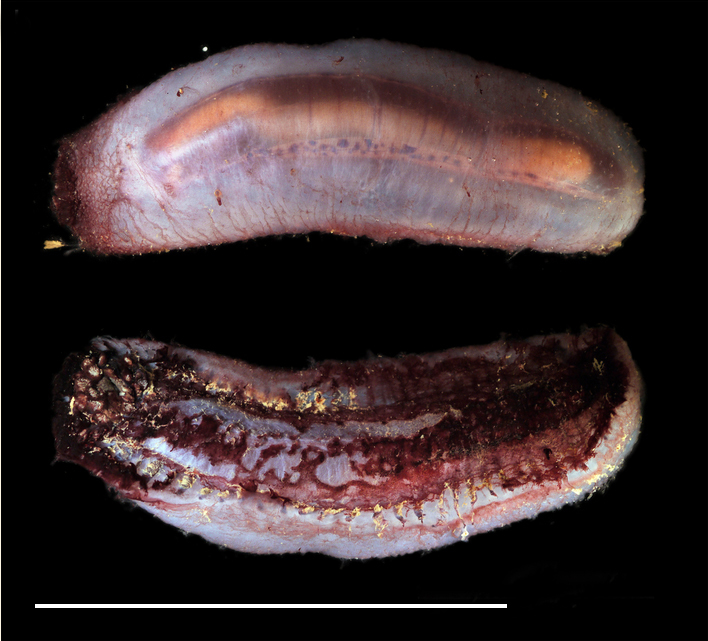
Benthodytes cf. typica after collection. Scale bar is 10 cm. Image attribution: AG Glover, TD Dahlgren & H Wiklund, Natural History Museum, London & Uni Research.

**Figure 56a.**
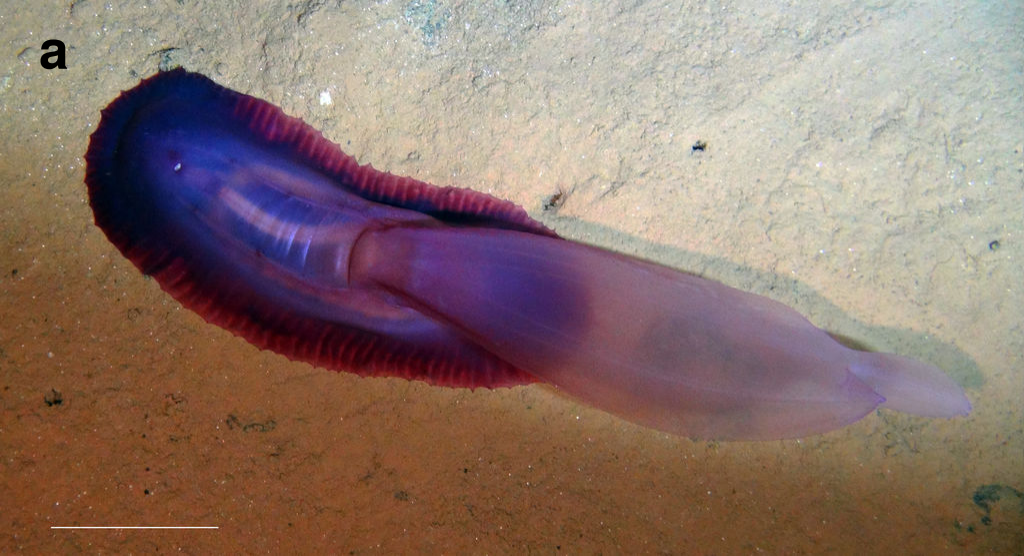
*Psychropotes semperiana* in situ on seafloor. Scale bar is 10 cm. Image attribution: DJ Amon & CR Smith, University of Hawai’i.

**Figure 56b.**
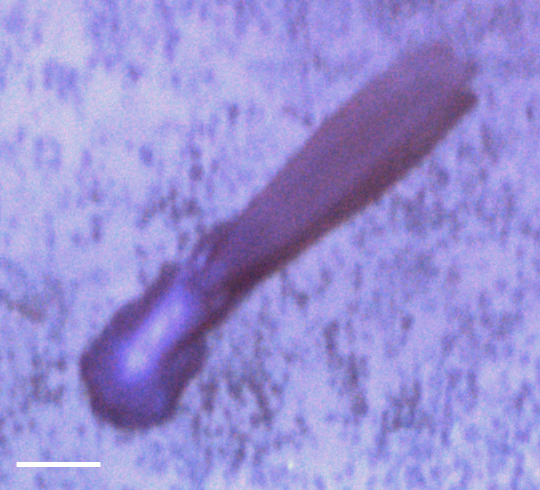
Psychropotes cf. semperiana in situ on seafloor. Scale bar is 10 cm. Image attribution: Woods Hole Oceanographic Institution.

**Figure 56c.**
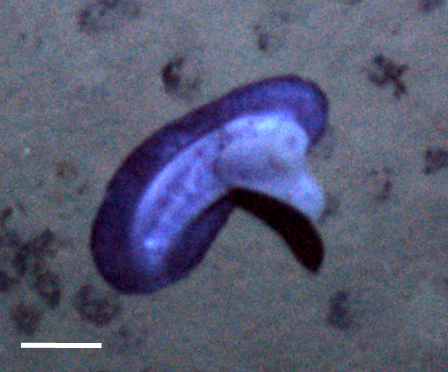
Psychropotes cf. semperiana in situ on seafloor. Scale bar is 10 cm. Image attribution: Woods Hole Oceanographic Institution.

**Figure 56d.**
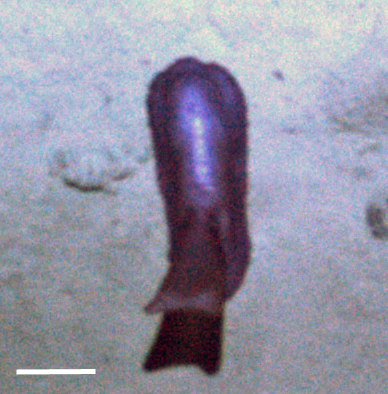
Psychropotes cf. semperiana in situ on seafloor. Scale bar is 10 cm. Image attribution: Woods Hole Oceanographic Institution.

**Figure 57.**
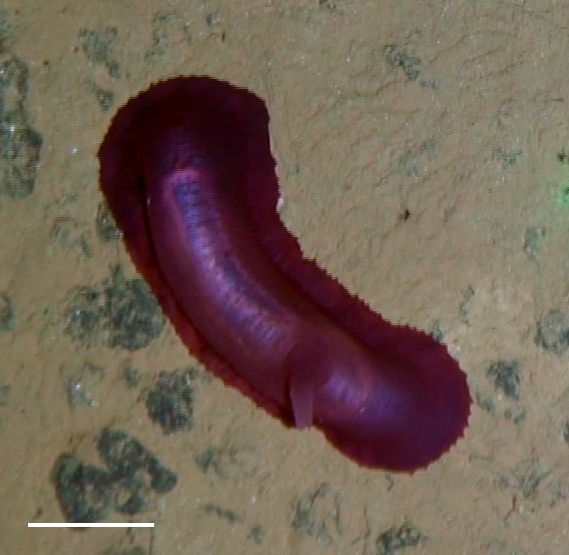
Psychropotes cf. verrucosa in situ on seafloor in the UK-1 exploration contract area. Image corresponds with the data above. Scale bar is 10 cm. Image attribution: DJ Amon & CR Smith, University of Hawai’i.

**Figure 58a.**
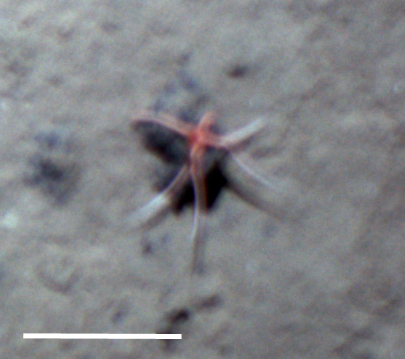
cf. Ophiuroidea morphospecies in situ on seafloor. Scale bar is 10 cm. Image attribution: Woods Hole Oceanographic Institution.

**Figure 58b.**
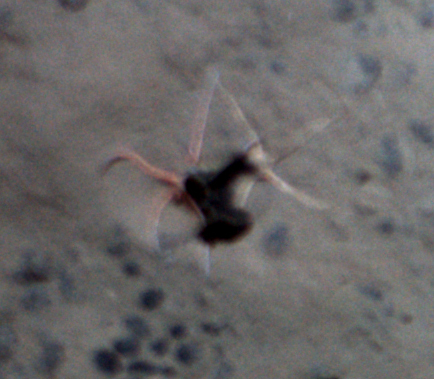
cf. Ophiuroidea morphospecies (left) and an unidentified ophiuroid (right) in situ on seafloor. Image attribution: Woods Hole Oceanographic Institution.

**Figure 58c.**
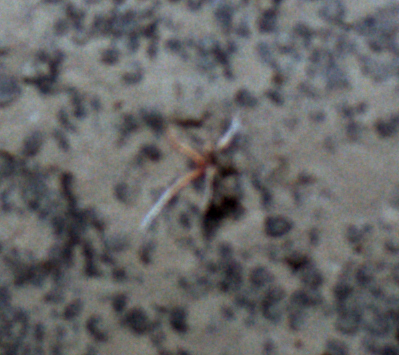
cf. Ophiuroidea morphospecies in situ on seafloor. Image attribution: Woods Hole Oceanographic Institution.

**Figure 59a.**
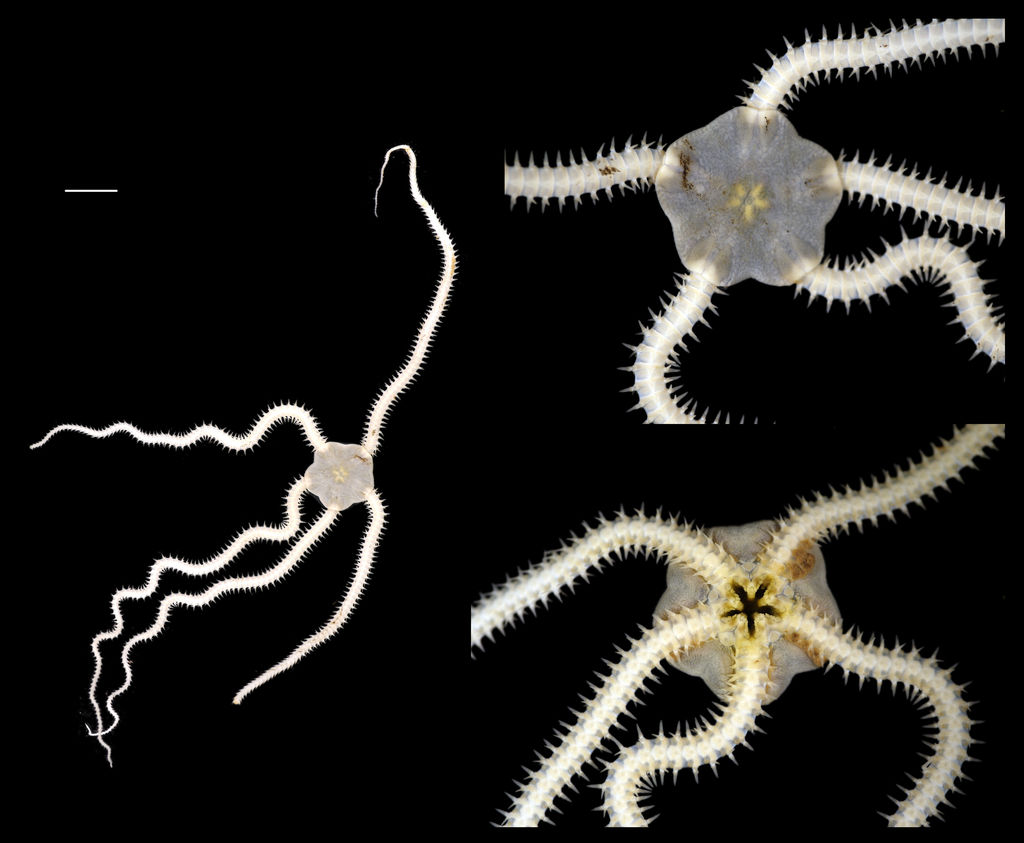
Amphioplus (Unioplus) daleus after collection. Scale bar is 1 cm. Image attribution: AG Glover, TD Dahlgren & H Wiklund, Natural History Museum, London & Uni Research.

**Figure 59b.**
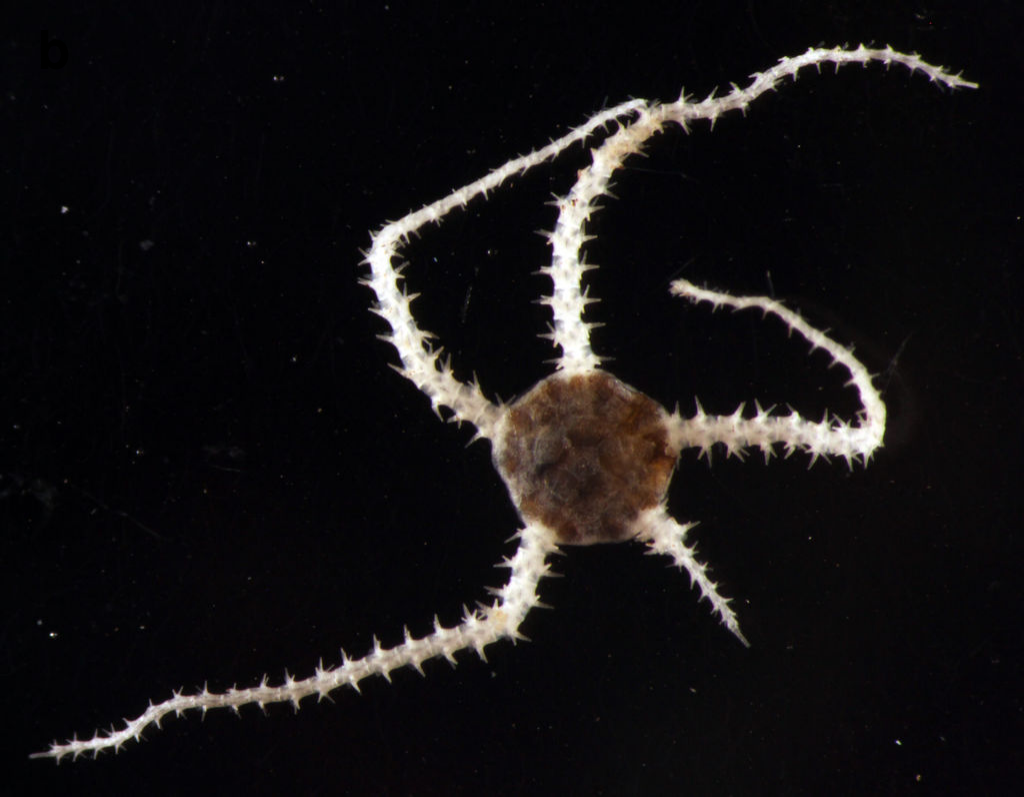
Amphioplus (Unioplus) daleus after collection. Image attribution: AG Glover, TD Dahlgren & H Wiklund, Natural History Museum, London & Uni Research.

**Figure 60a.**
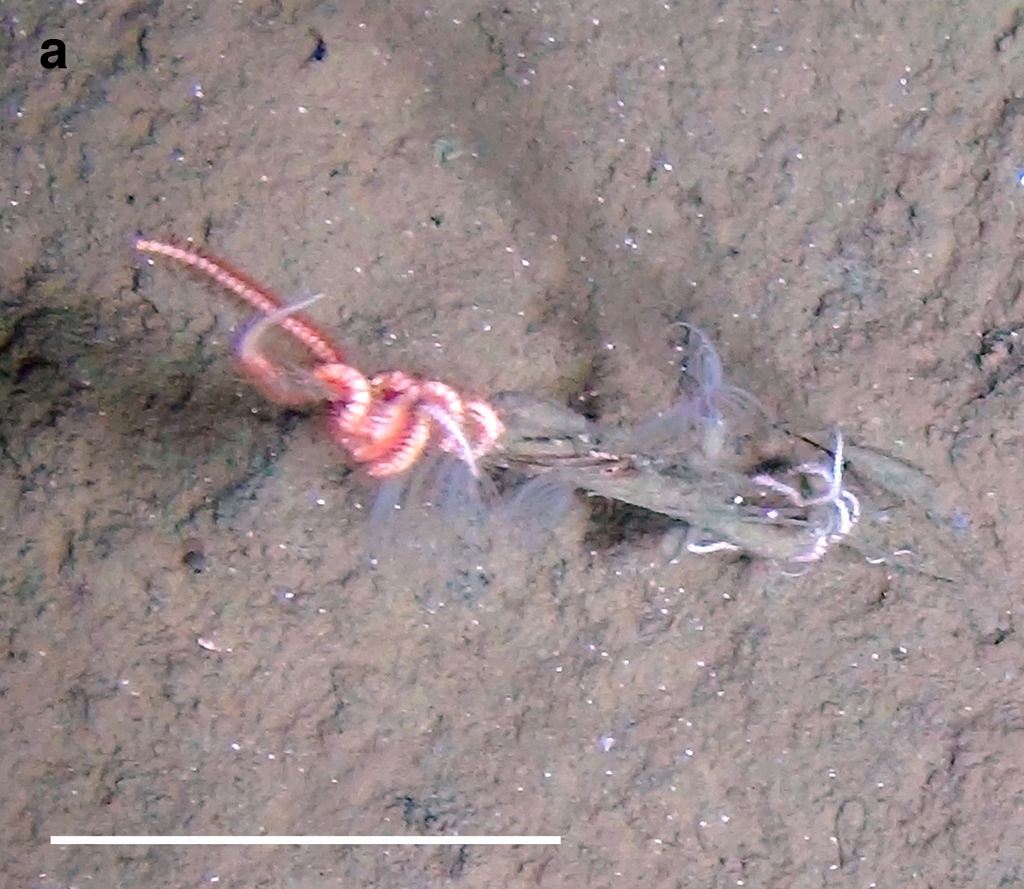
cf. *Ophiacantha* morphospecies in situ attached to a dead sponge stalk. Scale bar is 10 cm. Image attribution: DJ Amon & CR Smith, University of Hawai’i.

**Figure 60b.**
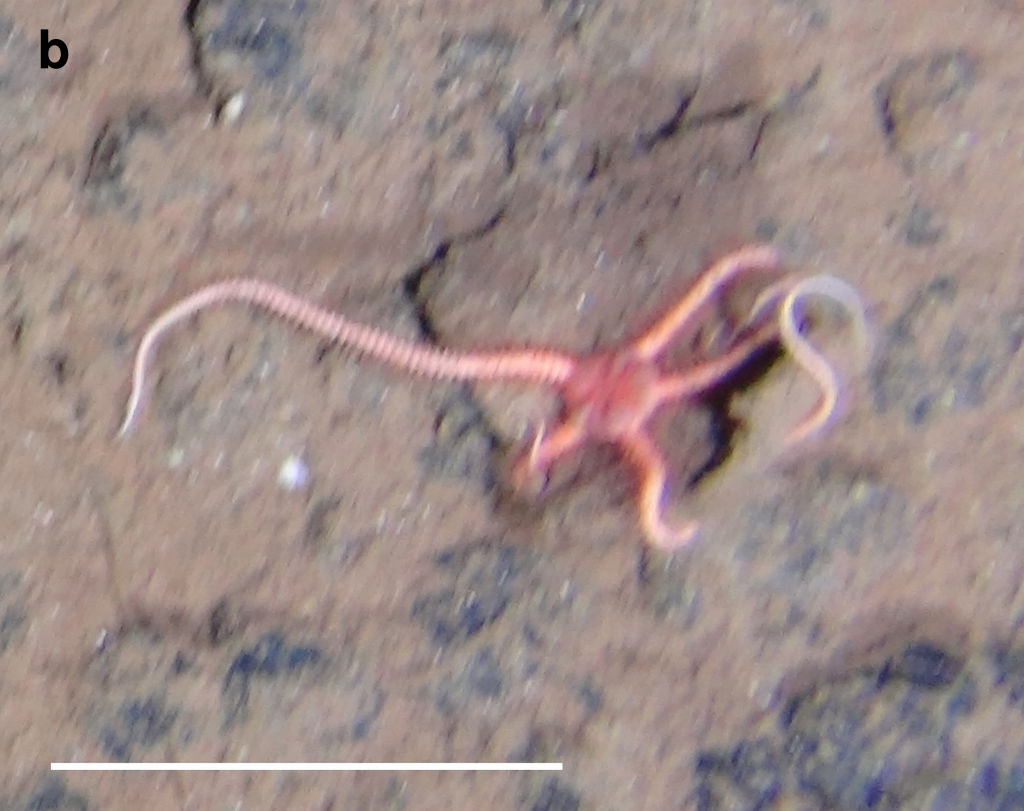
cf. *Ophiacantha* morphospecies in situ on seafloor. Scale bar is 10 cm. Image attribution: DJ Amon & CR Smith, University of Hawai’i.

**Figure 61a.**
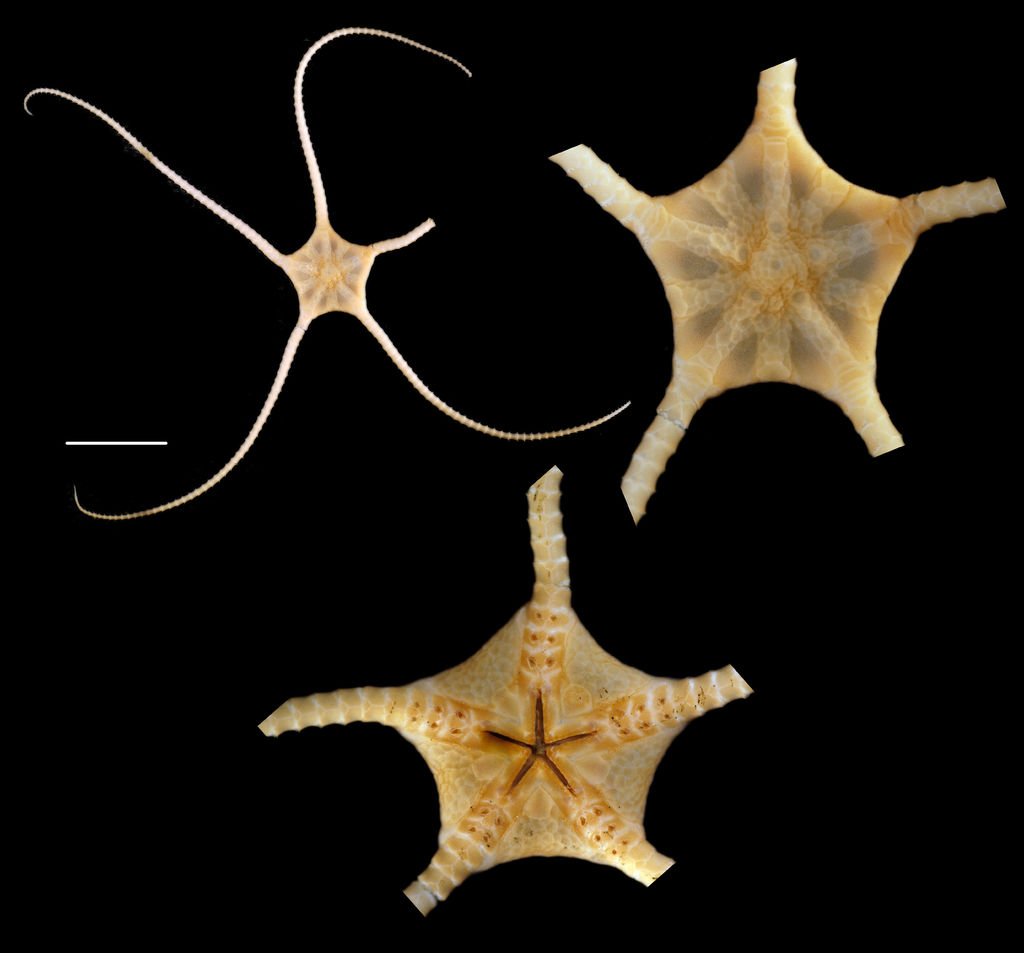
Ophiosphalma cf. glabrum after collection. Scale bar is 2 cm. Image attribution: AG Glover, TD Dahlgren & H Wiklund, Natural History Museum, London & Uni Research.

**Figure 61b.**
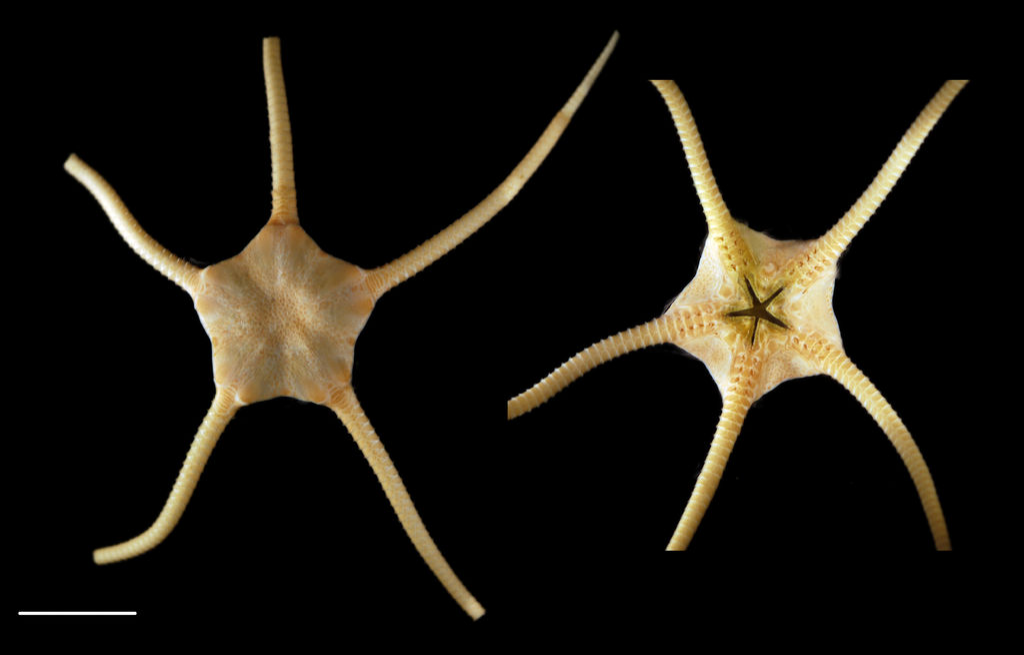
Ophiosphalma cf. glabrum after collection. Scale bar is 2 cm. Image attribution: AG Glover, TD Dahlgren & H Wiklund, Natural History Museum, London & Uni Research.

**Figure 61c.**
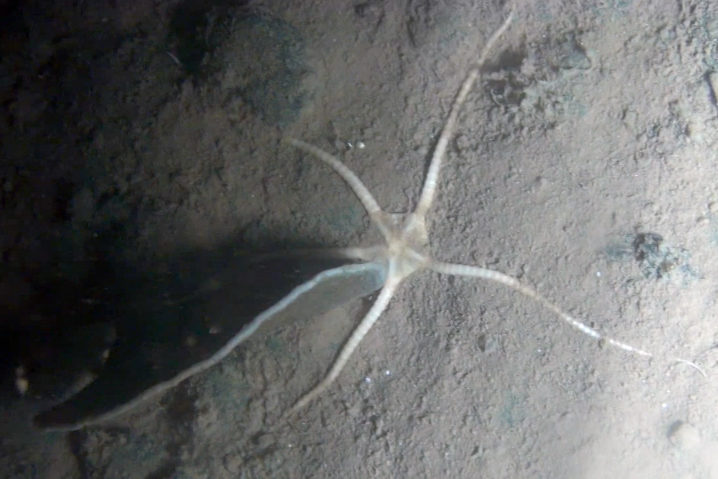
Ophiosphalma cf. glabrum in situ on seafloor. Image attribution: DJ Amon & CR Smith, University of Hawai’i.

**Figure 62.**
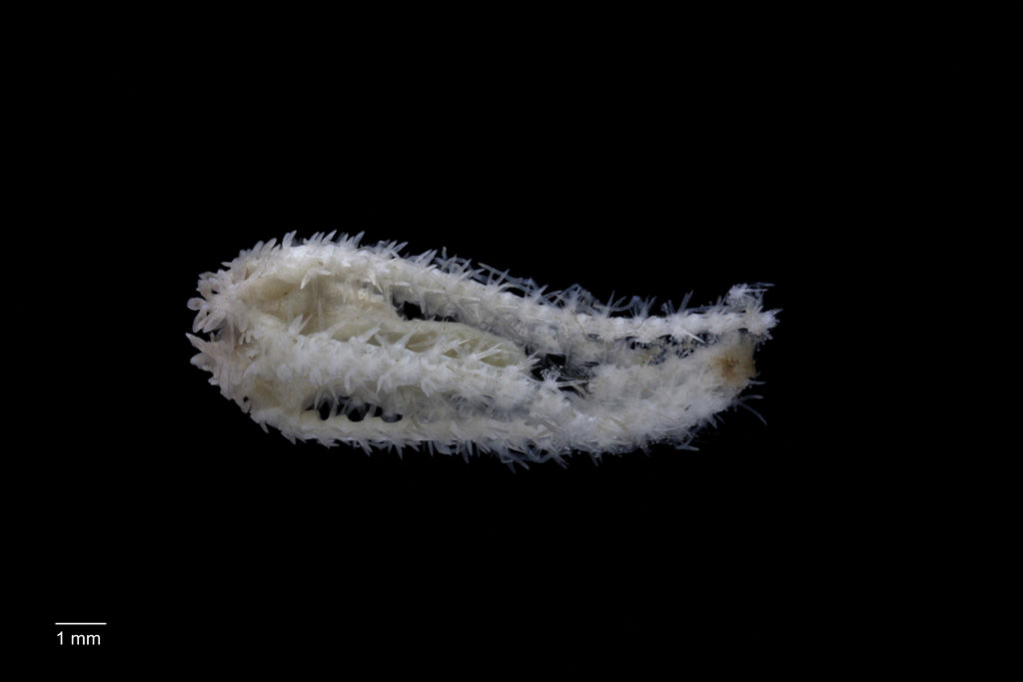
*Ophiotholia* morphospecies after collection from the UKSRL exploration contract area. Scale bar is 1 cm. Image attribution: C Harding, Museums Victoria.

**Figure 63a.**
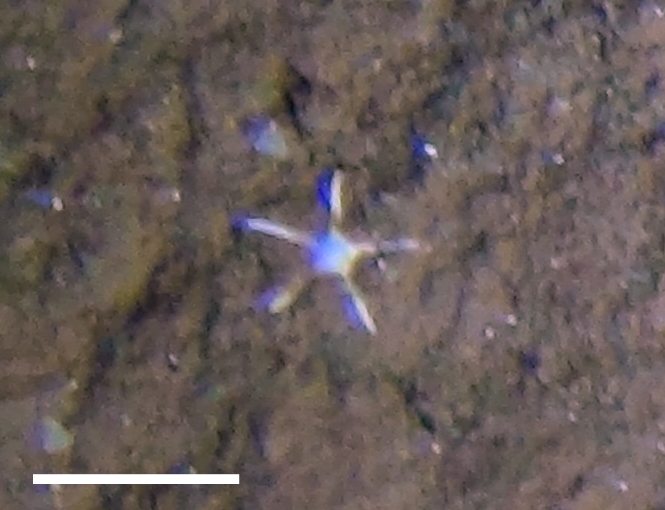
Ophiotypa cf. simplex in situ on seafloor. Scale bar is 2 cm. Image attribution: DJ Amon & CR Smith, University of Hawai’i.

**Figure 63b.**
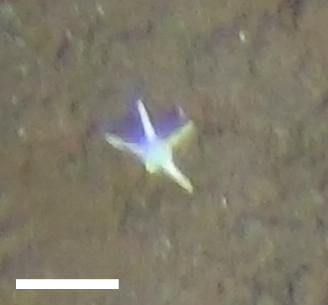
Ophiotypa cf. simplex in situ on seafloor. Scale bar is 2 cm. Image attribution: DJ Amon & CR Smith, University of Hawai’i.

**Figure 63c.**
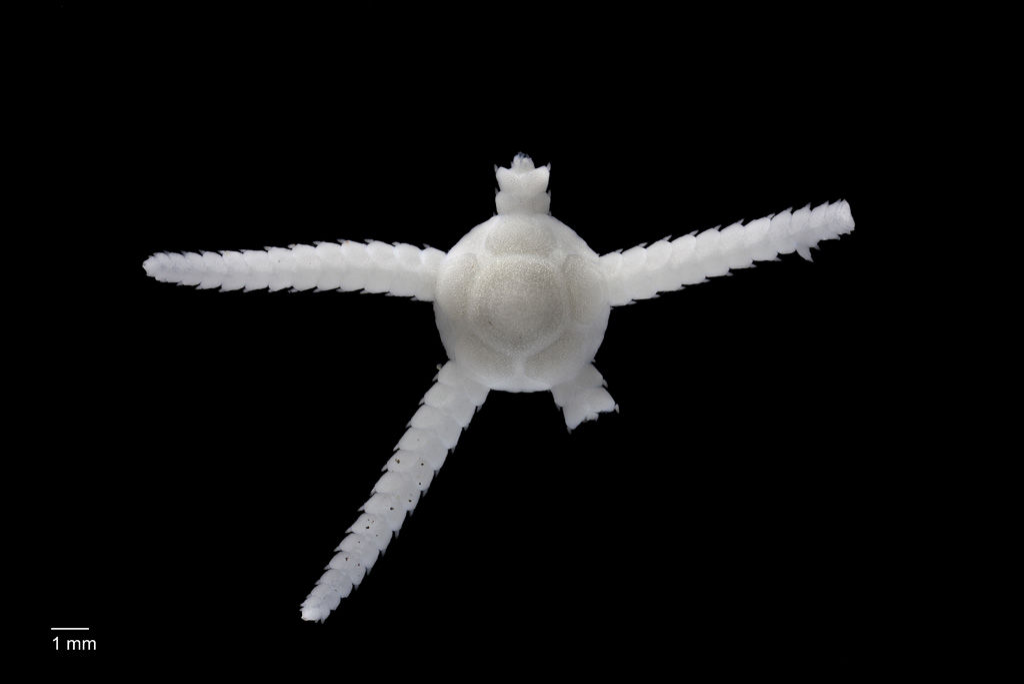
Aboral view of *Ophiotypa simplex* after collection. Scale bar is 1 mm. Image attribution: C Harding, Museums Victoria.

**Figure 63d.**
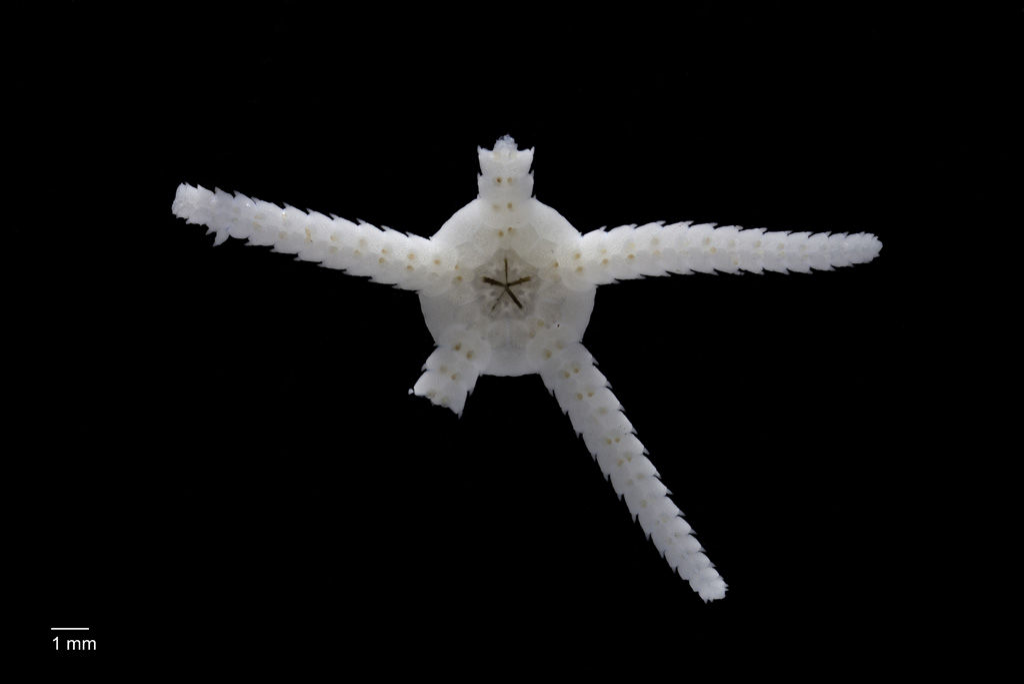
Oral view of *Ophiotypa simplex* after collection. Scale bar is 1 mm. Image attribution: C Harding, Museums Victoria.
